# Identification of Reference Genes for RT-qPCR Data Normalization in *Cannabis sativa* Stem Tissues

**DOI:** 10.3390/ijms17091556

**Published:** 2016-09-15

**Authors:** Lauralie Mangeot-Peter, Sylvain Legay, Jean-Francois Hausman, Sergio Esposito, Gea Guerriero

**Affiliations:** 1 Environmental Research and Innovation (ERIN), Luxembourg Institute of Science and Technology (LIST), L-4362 Esch/Alzette, Luxembourg; lauralie.mangeot@yahoo.fr (L.M.-P.); sylvain.legay@list.lu (S.L.); jean-francois.hausman@list.lu (J.-F.H.); 2 Dipartimento di Biologia, Università di Napoli “Federico II”, Via Cinthia, I-80126 Napoli, Italy; sergio.esposito@unina.it

**Keywords:** gene expression, reference genes, *Cannabis sativa*, cell wall, lignification

## Abstract

Gene expression profiling via quantitative real-time PCR is a robust technique widely used in the life sciences to compare gene expression patterns in, e.g., different tissues, growth conditions, or after specific treatments. In the field of plant science, real-time PCR is the gold standard to study the dynamics of gene expression and is used to validate the results generated with high throughput techniques, e.g., RNA-Seq. An accurate relative quantification of gene expression relies on the identification of appropriate reference genes, that need to be determined for each experimental set-up used and plant tissue studied. Here, we identify suitable reference genes for expression profiling in stems of textile hemp (*Cannabis sativa* L.), whose tissues (isolated bast fibres and core) are characterized by remarkable differences in cell wall composition. We additionally validate the reference genes by analysing the expression of putative candidates involved in the non-oxidative phase of the pentose phosphate pathway and in the first step of the shikimate pathway. The goal is to describe the possible regulation pattern of some genes involved in the provision of the precursors needed for lignin biosynthesis in the different hemp stem tissues. The results here shown are useful to design future studies focused on gene expression analyses in hemp.

## 1. Introduction

The identification of appropriate reference genes used for normalization and whose expression is not affected by the experimental factors is a crucial step in gene expression studies performed using real-time PCR (a.k.a. quantitative PCR, or RT-qPCR). The choice of unsuitable reference genes can compromise the results and may lead to wrong interpretations of biological phenomena.

The literature is constellated by studies focusing on the selection of reference genes in different plant species, tissues, conditions [1,2,3,4]; a list of potential candidates whose orthologs can be tested in the plant/tissue of choice is usually provided. Several types of software are available to assess the stability of a set of candidate reference genes [5,6,7,8] and a ranking is provided to show those most stable. The comparison of expression patterns in plants subjected to a combination of abiotic stresses [9], or tissues showing strong differences in terms of composition and developmental stage, poses a challenge when data have to be normalized. In these circumstances, if a tissue maximization design is used [10], then reference genes suitable for all the conditions/tissues studied need to be identified. This might require the screening of several candidates, given the heterogeneity of the experimental conditions tested.

**Figure 1:**
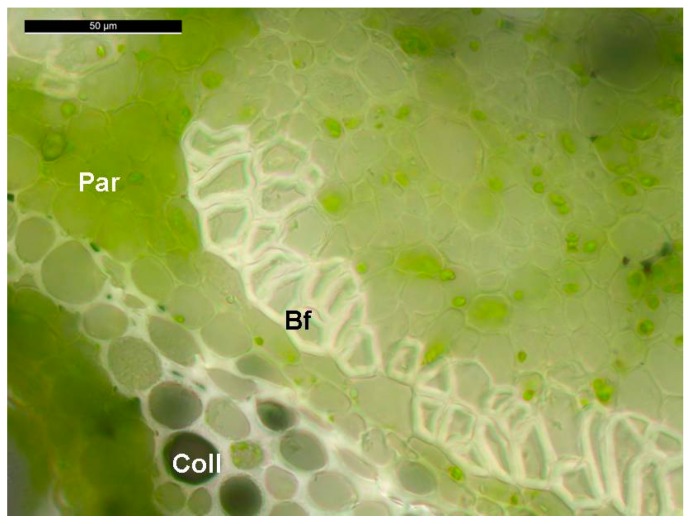
Representative cross section of the stem of an adult hemp plant (1.5 months) showing the bast fibres with the typical G-layer. Bf: bast fibres; Par: parenchyma; Coll: collenchyma.

One example of experimental heterogeneity is the study of gene expression in the stems of fibre crops, like textile hemp (*Cannabis sativa* L.), since they are characterized by tissues differing dramatically in their composition [11]. The stems of hemp are composed of cortical tissues harbouring cellulose-rich sclerenchyma fibres (which mechanically support the phloem, a.k.a. bast fibres) and a woody core (often referred to as hurd or shiv). These tissues can be easily isolated, since the cortex can be peeled off and subsequently processed to separate the bast fibres from the epidermis/parenchyma/collenchyma. While the core fibres are lignified and characterized by the typical secondary cell wall layers S1-S2-S3, bast fibres possess a gelatinous layer (G-layer; Figure 1) composed of crystalline cellulose which is similar to that found in tension wood [12]. The G-layer of bast fibres, however, does not exert the same contractile function as in tension wood [13]. It should be noted that hemp stems, unlike the other fibre crop flax (*Linum usitatissimum*), possess also secondary bast fibres which are shorter and more lignified than primary fibres and originate from the cambium [11].

Besides the differences in tissue composition, hemp stems additionally show a basipetal lignification gradient: younger internodes at the top are indeed rapidly elongating and the bast fibres show a relatively thin cell wall, while older internodes at the base of the stem cease elongation, synthesize a thick secondary cell wall and are more lignified. The transition from elongation to cell wall thickening is marked by an empirically-determined point, called the “snap point” [14].

The heterogeneous lignification of its stem tissues makes hemp very interesting as a model to study cell wall-related processes: the inner/outer tissues of the same internode, as well as those collected from younger/older internodes along the stem, can be studied to carry out high-throughput gene expression profiling. Such studies allow addressing molecular questions on, e.g., the regulation of bast fibre cell wall formation and maturation.

Some studies are available on the identification of reference genes in other fibre crops, notably flax (*Linum usitatissimum*) [15], kenaf (*Hibiscus cannabinus*) [16] and jute (*Corchorus capsularis*) [17]. These studies on one hand show that care should be taken when choosing the reference genes, as their stability might change depending on the tissue analysed and the treatment studied, and on the other hand they highlight that the different algorithms available for the evaluation of reference genes may not generate an identical ranking list (see Section 2.2).

No specific study focused on reference genes for RT-qPCR data normalization in hemp is yet available to our knowledge. Given the importance of hemp as a multi-purpose crop for different industrial applications, it is desirable to identify candidate reference genes to perform gene expression analyses. In this work we fill this gap by providing a list of candidates suitable for RT-qPCR data normalization in hemp stem tissues (i.e., bast fibres and shivs). Additionally, we validate them by performing an expression profiling of genes involved in the provision of precursors for lignin biosynthesis. More specifically, we chose genes coding for transaldolase isoforms of the pentose phosphate pathway, *TRA1* and *TRA2*, which synthesize erythrose 4-phosphate, and 3-deoxy-d-arabino-heptulosonate 7-phosphate synthase (*DHS*) *1* and *2*, diverting C-skeletons towards the shikimate pathway.

Given the importance of hemp woody and bast fibres in the textile and biocomposite sectors [18], our study is a useful guide for future molecular studies centred on this economically important fibre crop.

## 2. Results and Discussion

### 2.1. Stability of Candidate Reference Genes in C. sativa Stem Tissues

In this study the stability of 12 potential reference genes from textile hemp was studied for RT-qPCR data normalization (Table 1).

These candidates comprise both well-known, widely-used reference genes (e.g., tubulin, actin, glyceraldehyde 3-phosphate dehydrogenase *GAPDH*, the translation elongation factor *EF2*, the eukaryotic translation initiation factor *eTIF4E*, cyclophilin *Cyclo*, the tonoplast intrinsic protein *TIP41*) and less frequent ones (notably the clathrin adaptor complex, histone3, the small GTP-binding protein *RAN*, the calcium-dependent protein kinase *CDPK* and the *F-box* gene). An analysis of the stability of the 12 candidate reference genes in hemp fibres and shivs was performed with different methods, namely geNorm^PLUS^ [5], NormFinder [6], BestKeeper [7], RefFinder [8] and the comparative delta-Ct method [19]. As can be seen in Table 2, the ranking of the candidate reference genes varied among the methods, which is dependent upon the different algorithms used.

To identify the most appropriate subset of genes for RT-qPCR data normalization in hemp stem tissues, the rankings generated by the five methods were compared and we looked for genes classified among the four most stable by at least three different computational techniques. As can be seen in Table 2, *CDPK* ranked among the four most stable genes with all the methods used. *TIP41* had among the lowest values according to NormFinder, Bestkeeper, the comparative delta-Ct method and RefFinder. *F-box* ranked among the most stable genes by geNorm^PLUS^, the comparative delta-Ct method and RefFinder; likewise, *eTIF4E* ranked as one of the most stable according to NormFinder, the comparative delta-Ct method and RefFinder.

**Figure 2:**
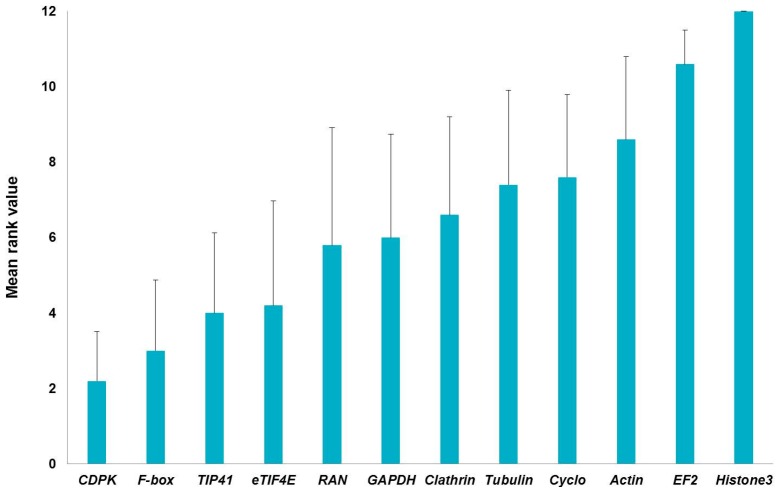
Global ranking of the reference genes relative to hemp hurds and bast fibres generated by assigning a number (from 1 to 12) to the stability coefficients (shown in Table 2) and by averaging them. Error bars refer to the standard deviation.

Interestingly, all the methods used to determine the stability of the 12 candidate reference genes agreed in ranking histone3 and *EF2* among the four least stable genes; actin and *Cyclo* were likewise assigned higher scores by three independent methods (Table 2). *EF2* was found to be amongst the least stable genes in the stems of another fibre crop, i.e., flax [15]: this might indicate that great variability in its expression is present in the different stem tissues (bast fibres and shivs) of fibre crops. We generated a graph (Figure 2) showing the global stability values by assigning a number (from 1 to 12, where 1 is the most stable and 12 the least stable) to the stability coefficients of Table 2 and by averaging them. As can be seen in Figure 2, the higher stabilities of *CDPK*, *F-box*, *TIP41*, *eTIF4E* are confirmed, when both stem tissues are considered together.

On the basis of the above-mentioned stability rankings, it is here proposed to include *CDPK*, *TIP41*, *F-box* and *eTIF4E* in the screening of reference genes for expression studies focused on hemp stem tissues. Clearly, their suitability in a given experimental set-up has to be checked prior to performing data normalization.

### 2.2. Optimal Number of Reference Genes in C. sativa Stem Tissues

**Figure 3:**
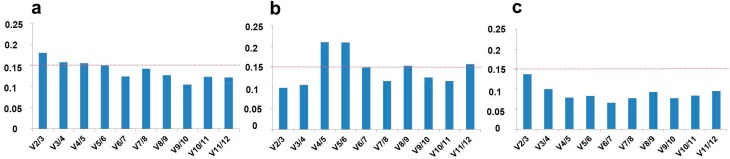
Optimal number of reference genes in hemp stem tissues as computed by geNorm^PLUS^. Pairwise variation (*Vn*/*Vn*+1) for (**a**) stem tissues; (**b**) bast fibres only; and (**c**) core only. The red dotted line represents the threshold (0.15).

Hemp stem tissues are characterized by evident differences in composition: the core is woody, while the bast fibres are cellulosic and poor in lignin [11]. It should be underlined that, particularly in expression studies on different tissues, it is necessary to determine the appropriate number of reference genes for a correct normalization. The software geNorm^PLUS^ estimates the optimal number of reference genes to use in a specific experimental set-up: this is quite important in studies focused on hemp stem tissues, in the light of their heterogeneity of cell types and of cell wall composition. We therefore used geNorm^PLUS^ to calculate the pairwise variation (*Vn*/*Vn*+1) between two consecutive normalization factors (*NFn* and *NFn*+1) and thus determine the optimal number (and best combination) of reference genes for the normalization of data obtained from hemp stem tissues. The software geNorm^PLUS^ recommended the use of five reference genes, namely tubulin, *CDPK*, *RAN*, clathrin and *F-box* for normalization (Figure 3a). The V value falls indeed below the cut-off threshold of 0.15 when all the five reference genes are taken together. These five genes showed the highest expression stability in the different hemp stem tissues (Figure S1).

The high heterogeneity of cell types (core with a parenchymatic pith, xylem vessels and fibres; cortex with extraxylary phloem fibres) and cell wall composition most likely explains the need of using five reference genes to normalize the RT-qPCR data. To verify this, the pairwise variation for the two tissues was calculated separately. As can be seen in Figure 3b,c, in both cases two reference genes (*EF2*/actin for the bast fibres and *CDPK*/tubulin for the shivs) were sufficient to normalize the data. It should be noted that *EF2*, actin and tubulin ranked among the least stable genes in the global ranking (Figure 2). In this respect it is not surprising that less stable genes in total stem tissues perform better when a specific tissue type is considered. For example a study on flax revealed that five Eukaryotic translation initiation factor genes were less stable when several tissues were considered together (stems, leaves, roots, flowers), but ranked among the most stable when specific tissue types were taken into account [15]. These results and ours highlight that, when working with heterogeneous biological samples (as is the case of fibre crop stems), it may be necessary to select different reference genes.

### 2.3. Validation of the Reference Genes in Hemp Stem Tissues

In order to validate the hemp stem tissues reference genes, as suggested by geNorm^PLUS^, the expression of four genes belonging to pathways providing metabolic precursors for the biosynthesis of aromatic amino acids and, more comprehensively, for important plant secondary metabolites, was analysed. These genes are transaldolase 1 and 2 (*TRA1*, *TRA2*, hemp orthologs of *At1g12230* and *At5g13420*) and 3-deoxy-d-arabino-heptulosonate 7-phosphate synthase 1 and 2 (*DHS1*, *DHS2*, hemp orthologs of *At4g45420* and *At4g33510*). These genes have been chosen because of their connection with lignin deposition and their reported roles in regulatory aspects of the lignification process (a STRING network analysis, showing the putative interactions in biological processes, is shown in Figure S2).

TRA1 and TRA2 are involved in the regenerative phase of the pentose phosphate pathway: they catalyse the formation of erythrose 4-phosphate, a central metabolite in its turn shunted to the production of aromatic amino acids via the shikimate pathway [20].

In *Arabidopsis thaliana* two genes coding for TRA are present, *TRA1*-*At1g12230* and *TRA2*-*At5g13420*; in *C. sativa* two orthologs are present as well (contig numbers in Table S1). Although located quite early in the metabolic branch leading to the provision of C-skeletons for aromatic acid biosynthesis, TRA2 was shown to have a specific role in lignification [21]: the stems of *Arabidopsis tra2* mutants had indeed lower lignin content and showed a higher S/G ratio.

DHS catalyses the first step of the shikimate pathway, which is crucial in shunting a relevant portion of fixed C towards the synthesis of different important metabolites [22]. The enzyme performs a condensation of phospho*enol*pyruvate with erythrose 4-phosphate to produce 3-deoxy-d-arabino-heptulosonate 7-phosphate. Three isoforms are present in *Arabidopsis* (*DHS1*-*At4g39980*, *DHS2*-*At4g33510* and *DHS3*-*At1g22410*), however in hemp only two contigs were retrieved (Table S1). Notably, two isoforms were also found in jute (*Corchorus capsularis*), one of which (called *CcDAHPS2*) was upregulated at early growth stages in the bast fibres of a developmental mutant (deficient lignified phloem fibre, *dlpf*) [23]. In *A. thaliana*, *DHS1* was shown to be induced by wounding and *Pseudomonas syringae* attack [24], which are processes involving changes in lignin deposition (“defence lignin” synthesis for example) [25].

**Figure 4:**
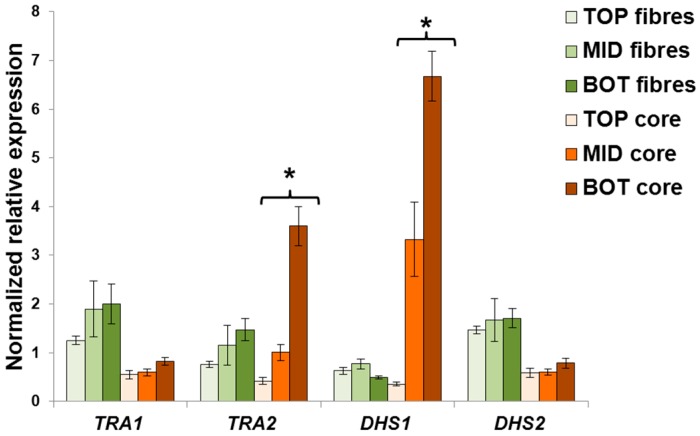
Expression analysis of *TRA1*, *TRA2*, *DHS1* and *DHS2* in the different stem tissues of *C. sativa*. Error bars indicate the standard error of the mean (*n* = 4). Stars indicate statistically significant values at the one-way ANOVA test (* *p* < 0.05).

In *C. sativa* a differential expression of *TRA2* and *DHS1* was observed in the core tissue collected at the top, middle and bottom of the stems (Figure 4), while no statistically relevant changes in these genes were obtained in the bast fibres. *TRA1* and *DHS2* show a more constitutive-level of expression among the different stem heights for each tissue considered, although their expression in bast fibres was higher as compared to the core (Figure 3; the difference between the bottom region of the fibres and the core was however not significant for *TRA1*). Intriguingly, *TRA2* and *DHS1* genes were strongly upregulated in the bottom region of the stem. This is expected, since a higher shunt of C-skeletons for the synthesis of secondary metabolites linked to lignification is required in the older regions of the hemp stem. Therefore, these results show that two different clusters of genes involved in stem tissue development exist in hemp and that they are differentially regulated in order to play different functions.

It is possible to propose a role in lignification for *TRA2* and *DHS1* and a potential bast fibre-related function for *TRA1* and *DHS2* (their expression is higher in the bast fibres as compared to the hurds, Figure 4). This hypothesis is supported by previous studies in which the maize TRA1 ortholog was shown to sustain basal metabolism and starch synthesis [26], but the total occurrence and presence of TRA activity is related to plant defence mechanisms involving the synthesis of secondary metabolites. It is worth noting that one of the fastest responses to pathogen attack in plants is programmed cell death and lignification of wounded tissues [27].

## 3. Materials and Methods

### 3.1. Plant Material and Growth Conditions

A fibre-variety of hemp (*Cannabis sativa* cv. Santhica 27) was studied in this work. Plants were grown as described in [25]. Four biological replicates, each composed of a pool of 13 plants, were used in this study. After six weeks of growth, samples were taken along three stem regions localized at different heights with respect to the snap point. The “TOP” segment corresponds to the internode right below the apex of the plants, the “MID” (middle) segment is the internode which contains the snap point and the “BOT” (bottom) segment is located two internodes below the “MID” sample. A segment of 2.5 cm was collected from the middle of each internode to avoid too much variation in gene expression, due to the varying developmental stages of the cell types. Fibres were separated from the parenchyma/collenchyma cortical tissues by gently pressing the collected stem peels in 80% ethanol with a pestle, as described in [28]. The fibres were then quickly blotted dry using autoclaved wipers (WypAll, Kimberley-Clark, Muller & Wegener, Luxembourg, Grand Duchy of Luxembourg) and immediately frozen in liquid nitrogen. The core of the stem segments was directly plunged in liquid nitrogen.

### 3.2. Gene Identification and Primer Design

The twelve reference genes were retrieved at the Medicinal Plant Genome Resource (http://medicinalplantgenomics.msu.edu/index.shtml; Table S1) by blasting the reported nucleotide sequences in other plant species. Specific gene primers were designed using Primer3Plus (http://www.bioinformatics.nl/cgi-bin/primer3plus/primer3plus.cgi) and checked using the OligoAnalyzer 3.1 tool from Integrated DNA technologies (http://eu.idtdna.com/calc/analyzer). Primer efficiencies were calculated by RT-qPCR using six serial dilutions of cDNA (25, 5, 1, 0.2, 0.04, 0.008 ng/μL). The primer sequences, their corresponding amplicon length and Tm, the amplification efficiencies and regression coefficients are indicated in Table 1. The alignment of the selected reference genes (Figure S3) with the orthologs from hop (*Humulus lupulus*) [29], marijuana (Purple Kush) [30] and flax (*L. usitatissimum*) was carried out using ClustalOmega (http://www.ebi.ac.uk/Tools/msa/clustalo/). The hop sequences were retrieved by blasting (BLASTN) the hemp nucleotide sequences at http://hopbase.cgrb.oregonstate.edu/blast. The flax sequences were obtained by blasting (BLASTX) the hemp nucleotide genes at https://phytozome.jgi.doe.gov/pz/portal.html. The *C. sativa* (Purple Kush, marijuana) sequences were obtained by carrying out a BLAT analysis at http://genome.ccbr.utoronto.ca/cgi-bin/hgBlat?command=start&org=C.+sativa& db=canSat3&hgsid=78813.

### 3.3. RNA Extraction, cDNA Synthesis and RT-qPCR

Total RNA was extracted using a modified CTAB extraction protocol combined with the RNeasy Plant Mini Kit (Qiagen, Leusden, The Netherlands) [28] according to the manufacturer’s instructions (including digestion with DNase). The RNA concentration and quality were measured for each sample by using a Nanodrop ND-1000 (Thermo Scientific, Villebon-sur-Yvette, France) and a 2100 Bioalyzer (Agilent, Santa Clara, CA, USA), respectively. In case of 230 nm contamination, samples were cleaned with a precipitation using ammonium acetate (NH_4_OAc) and a subsequent wash in ethanol (1/10 volume of NH_4_OAc in 2.5 volumes of 100% cold ethanol, incubation 60 min at −20 °C, centrifugation at 12,000× *g* for 20 min at 4 °C, wash with 1.5 mL 75% cold ethanol, centrifugation at 12,000× *g* for 5 min at 4 °C, air-drying and re-suspension of the pellet in 20 μL of RNase-free water).

The extracted RNA was retrotranscribed into cDNA using the ProtoScript II reverse transcriptase (New England Biolabs, Leiden, The Netherlands) and random primers, according to the manufacturer’s instructions. The synthesized cDNA was diluted to 2 ng/μL and used for the RT-qPCR analysis in 384-well plates. An automated liquid handling robot (epMotion 5073, Eppendorf, Hamburg, Germany) was used to prepare the 384-well plates. The RT-qPCR reactions were set up and run according to [31]. To check the specificity of the amplified products, a melt curve analysis was performed. The expression of the *TRA1*, *TRA2*, *DHS1*, *DHS2* genes was calculated using qBase^PLUS^ [10] (version 2.5, Biogazelle, Ghent, Belgium) by using the reference genes indicated by the geNorm^PLUS^ analysis. Statistics were performed using a one-way ANOVA, as implemented in qBase^PLUS^.

## 4. Conclusions

In this study, 12 candidate reference genes were analysed to test their stability and we validate their use in data normalization on hemp stems. The data shown highlight that it may be necessary to select different reference genes when heterogeneous biological samples are studied, as is the case of the contrasting hemp stem tissues. The studied reference genes can not only be used on textile hemp varieties, but also represent candidates to test on oil and drug *Cannabis* varieties. Additionally, the 12 reference genes here reported can eventually be tested in expression studies focused on close plant species, as for example the Cannabaceae member *H. lupulus*, given the overall sequence homology (Figure S3). The expression analysis of genes involved in the non-oxidative phase of the pentose phosphate pathway and the shikimate pathway, notably *TRA1* and *TRA2*, *DHS1* and *DHS2* have also been studied. It was possible to identify isoforms potentially involved in lignification. Further investigations are required to define the roles of other genes playing in the different cell wall-related processes of the hemp stem: lignification requires a complex network of metabolic pathways and signals, different to those required for bast fibre synthesis. It will be interesting in the future to study them functionally. For example, our study opens up the way to future studies centred on the pentose phosphate pathway, which is a central metabolic pathway for both the primary and secondary plant metabolism. The role of glucose-6-phosphate dehydrogenase, a central player in the pathway [32,33,34,35], can be addressed to understand its contribution to the shunt of precursors needed for lignin biosynthesis.

## Figures and Tables

**Table 1:** List of candidate reference genes used in the study. The details concerning the primer sequences, amplicon length and Tm, PCR efficiency and regression coefficient are given.

Name	Sequence (5′→3′)	Amplicon Length (bp)	Amplicon Tm (°C)	PCR Efficiency (%)	Regression Coefficient (R^2^)
ActinFwd	TTGCTGGTCGTGATCTTACTG	148	83	90.8	0.993
ActinRev	GTCTCCATCTCCTGCTCAAAG				
eTIF4EFwd	AGTGGGAAGATCCTGAATGTGC	150	81	95.1	0.997
eTIF4ERev	TTGCGACCACACCACAAATC				
EF2Fwd	ACGCAACAGCTATCAGGAAC	113	80.3	92.3	0.998
EF2Rev	TGCAAAGACACGACCAAAGG				
GAPDHFwd	AATCGCAACCCAAACTCTGC	123	81.1	99.4	0.995
GAPDHRev	AGTGGCCGTTGCTTTAATGG				
CycloFwd	ACAACATGTCGAACCCCAAG	106	81.4	93.8	0.998
CycloRev	TCAGCGGTTTTTGGCGTAAC				
RANFwd	TTTGGAGACTTCAGCACTGG	129	81.8	97.8	0.998
RANRev	GCAGGGTTACCATTTCCTTG				
F-boxFwd	TATCGGCGGAGAGATTTGAG	77	78.4	99.5	0.975
F-boxRev	TAAGCCCTTCCCTTGATTCC				
ClathrinFwd	TGTCAGTTTTGTGCCACCAG	139	80.3	98.3	0.998
ClathrinRev	TCCATGCGTGTTCTACCAAG				
Histone3Fwd	TGAAGAAGCCTCATCGGTTC	127	82.9	96.1	0.998
Histone3Rev	TCTTGAGCGATTTCCCTGAC				
TIP41Fwd	TGAACAGTGGGGAGAAAAGC	144	80.3	100.2	0.989
TIP41Rev	GCTTCCTGTTTCCATCCAAG				
TubulinFwd	ATAACTGTACTGGGCTTCAAGG	110	84	97.5	0.999
TubulinRev	CCTGTGGAGATGGGTAAACTG				
CDPKFwd	GGTGGCTTTGCTTCTCTTTG	86	78.7	97	0.986
CDPKRev	GTCAAACCCCTTTTCACACC				
TA1Fwd	TTCGAGAAGTTCCCTCCAAC	118	81.5	92.8	0.999
TA1Rev	AGCCATATCCACAGCATTCC				
TA2Fwd	CTAGCAACCCAGCGATTTTC	126	81.3	97	0.998
TA2Rev	ACCACAAGCTCCCAATATGC				
DHS1Fwd	TGAGACTTTCCCTCCGATTG	144	84.6	96.8	0.998
DHS1Rev	TCAGCACAATCTCCACCTTG				
DHS2Fwd	TATCAAGGCTGTTCGTGGAG	129	81.8	103	0.997
DHS2Rev	AGGTGCTTTGATGGTGTTCC				

**Table 2:** Ranking of the 12 candidate reference genes according to the different methods used.

GeNorm^PLUS^		NormFinder		BestKeeper		Comparative delta-*C*t		RefFinder	
Gene	Stability Coeff.	Gene	Stability Coeff.	Gene	Stability Coeff.	Gene	Stability Coeff.	Gene	Stability Coeff.
*Histone3*	1.22	*Histone3*	0.812	*Histone3*	1.58	*Histone3*	1.678	*Histone3*	12
*EF2*	1.135	*EF2*	0.743	*RAN*	1.085	*EF2*	1.549	*EF2*	10.462
*Actin*	1.061	*Tubulin*	0.579	*Tubulin*	1.059	*Actin*	1.343	*Actin*	8.409
*Cyclo*	1.028	*Cyclo*	0.523	*EF2*	0.926	*GAPDH*	1.335	*Cyclo*	6.701
*GAPDH*	0.959	*Actin*	0.518	*Clathrin*	0.926	*Clathrin*	1.33	*Clathrin*	6.447
*eTIF4E*	0.857	*Clathrin*	0.504	*eTIF4E*	0.888	*Cyclo*	1.319	*Tubulin*	5.948
*TIP41*	0.798	*TIP41*	0.502	*F-box*	0.885	*RAN*	1.302	*GAPDH*	5.635
*Tubulin*	0.703	*GAPDH*	0.462	*Actin*	0.87	*Tubulin*	1.299	*RAN*	4.461
*CDPK*	0.599	*RAN*	0.453	*Cyclo*	0.718	*TIP41*	1.241	*eTIF4E*	3.742
*RAN*	0.503	*Fbox*	0.452	*CDPK*	0.699	*F-box*	1.234	*TIP41*	2.913
*Clathrin*	0.47	*CDPK*	0.272	*GAPDH*	0.681	*eTIF4E*	1.155	*F-box*	2.913
*F-box*	0.45	*eTIF4E*	0.257	*TIP41*	0.601	*CDPK*	1.091	*CDPK*	1.861

## Supplementary Materials

Supplementary materials can be found at www.mdpi.com/1422-0067/17/9/1556/s1.

## Abbreviations

RT-qPCR	Real-Time PCR
G-layer	Gelatinous layer
