# Supplementary material for: Identification of Reference Genes for RT-qPCR Data Normalization in Cannabis sativa Stem Tissues
Source: Int J Mol Sci. 2016 Sep 15;17(9):1556. doi: 10.3390/ijms17091556 (PMC5037828; doi:10.3390/ijms17091556)
Supplement: Supplementary file 1 [file ijms-17-01556-s001.pdf]

Supplementary Materials: Identification of Reference Genes for RT-qPCR Data Normalization in *Cannabis sativa* Stem Tissues

Lauralie Mangeot-Peter, Sylvain Legay, Jean-Francois Hausman, Sergio Esposito and Gea Guerriero

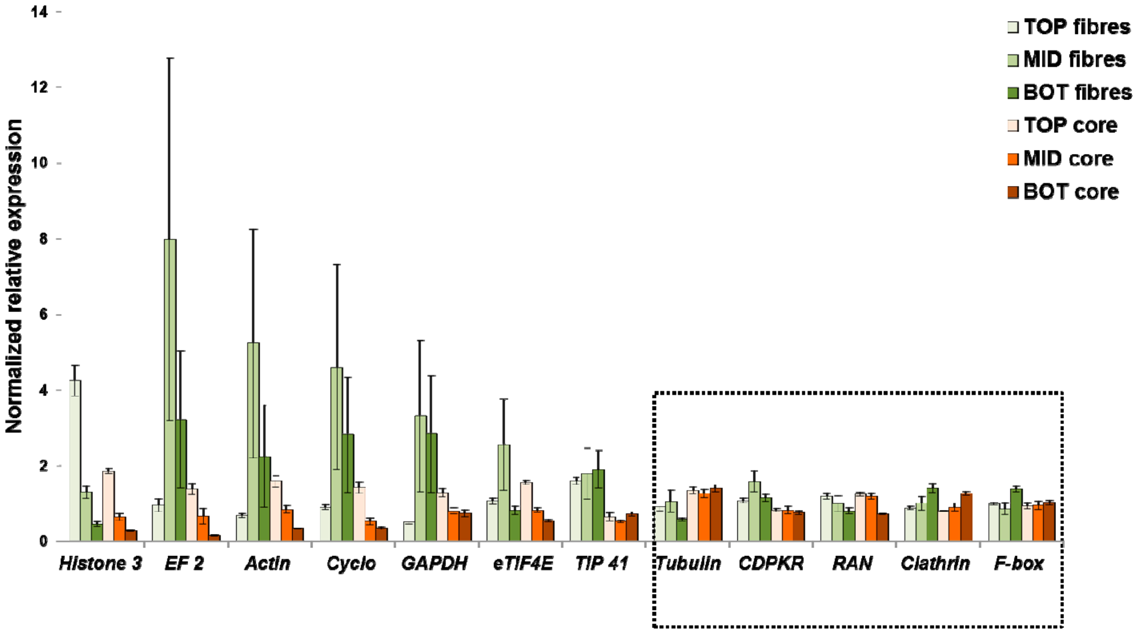

Figure S1. Expression of the different reference genes in hemp stem tissues. Error bars indicate the standard error of the mean (*n* = 4). The boxed genes correspond to those necessary for normalization when both types of hemp stem tissues are considered, according to geNorm^{PLUS}.

Table S1. Hemp reference and target genes analyzed in this study with relative accession numbers at the Medicinal Plant Genome Resource.

Gene Name	Locus Number
Histone3	csa_locus_1853_iso_4_len_1208_ver_2
EF2	csa_locus_674_iso_1_len_2081_ver_2
Actin	csa_locus_2263_iso_5_len_1488_ver_2
Cyclo	csa_locus_6842_iso_2_len_854_ver_2
GAPDH	csa_locus_1460_iso_2_len_1872_ver_2
eTIF4E	csa_locus_9035_iso_2_len_909_ver_2
TIP41	csa_locus_3380_iso_1_len_1164_ver_2
Tubulin	csa_locus_11261_iso_1_len_1086_ver_2
CDPK	csa_locus_8286_iso_2_len_2019_ver_2
RAN	csa_locus_1455_iso_4_len_1015_ver_2
Clathrin	csa_locus_5464_iso_6_len_1880_ver_2
F-box	csa_locus_16122_iso_2_len_2075_ver_2
TRA1	csa_locus_4950_iso_5_len_1428_ver_2
TRA2	csa_locus_5284_iso_4_len_790_ver_2
DHS1	csa_locus_3880_iso_9_len_1916_ver_2
DHS2	csa_locus_1565_iso_10_len_932_ver_2

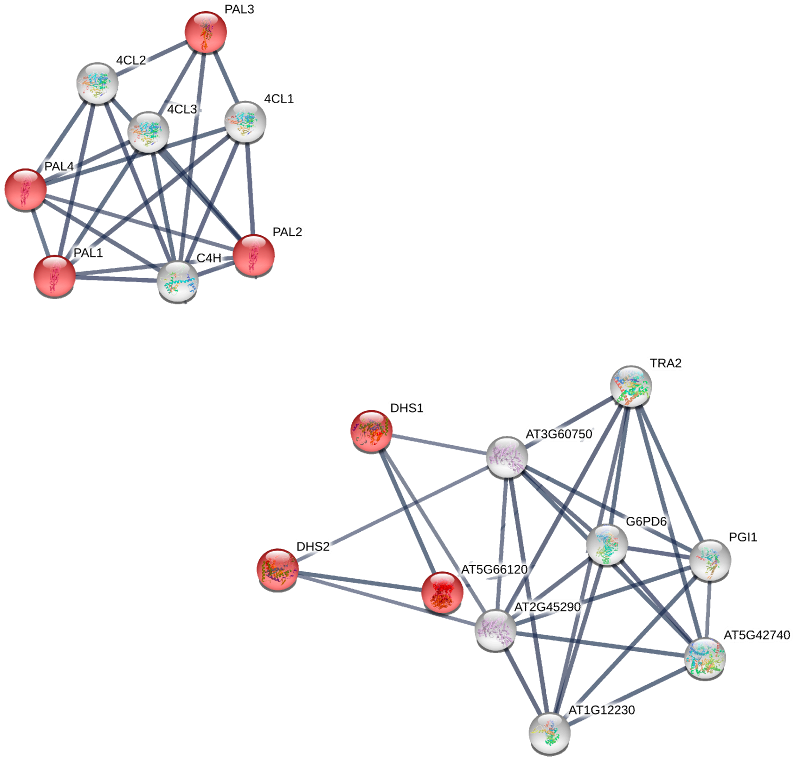

Figure S2. STRING network (http://string-db.org/) with medium confidence interaction score (0.400). The aromatic amino acid metabolic process is highlighted in red. The thicker lines correspond to an edge confidence >0.900, the thinner lines are >0.400. PAL: phenylalanine ammonia lyase; 4CL: 4-coumarate: 4-coumarate-CoA ligase; C4H: cinnamate 4-hydroxylase; PGI and AT5G42740: glucose-6-phosphate isomerases; G6PD: glucose-6-phosphate dehydrogenase; AT3G60750 and AT2G45290: transketolases.

HISTONE3

```
Marijuana-C32096195                               ------------------------------------------------------------
L.usitatissimum-LUS10013948                       ATGAAGGATCCTTCTGTCCACCTCATCCATCCGCTCCATTACCAATTCCCACCGTTGATT
Textile-hemp-csa_locus_1853_iso_4_len_1208_ver_2  ------------------------------------------------------------
H.lupulus-HL.SW.V1.0.G020502.1                    ------------------------------------------------------------

Marijuana-C32096195                               ------------------------------------------------------------
L.usitatissimum-LUS10013948                       ATCCCTCCACCATCCAACCGCCACAATCTCTTCCTCTACCGCAATAAAATCCCACCATTT
Textile-hemp-csa_locus_1853_iso_4_len_1208_ver_2  ----------------------------------------------------------TT
H.lupulus-HL.SW.V1.0.G020502.1                    ------------------------------------------------------------

Marijuana-C32096195                               ------------------------------------------------------------
L.usitatissimum-LUS10013948                       TCTTCACCGTTCATCATCATCAGTACCAAACATCAGCTCAGAATCCCAAATCTACCGATT
Textile-hemp-csa_locus_1853_iso_4_len_1208_ver_2  TCCTC---AA---GCTTCTCAAGAACAAAACAAA----CAGA---------CAAACACAG
H.lupulus-HL.SW.V1.0.G020502.1                    ------------------------------------------------------------

Marijuana-C32096195                               ------------------------------------------------------------
L.usitatissimum-LUS10013948                       CCCTACTCCTCCCTCTCTCATTCAATGGCTCGCACCAAGCAAACAGCTCGCAAGTCCACC
Textile-hemp-csa_locus_1853_iso_4_len_1208_ver_2  CAAAGAACTAAGCTTTCTCAATCAATGGCTCGTACCAAGCAGACTGCTCGCAAATCGACC
H.lupulus-HL.SW.V1.0.G020502.1                    ------------------------ATGGCTCGTACCAAGCAAACTGCTCGCAAGTCGACC

Marijuana-C32096195                               ------------------------------------------------------------
L.usitatissimum-LUS10013948                       GGAGGCAAGGCACCAAGGAAGCAGCTGGCCACCAAGGCAGCAAGGAAGTCAGCTCCGGCC
Textile-hemp-csa_locus_1853_iso_4_len_1208_ver_2  GGAGGCAAGGCCCCAAGGAAGCAGCTGGCGACTAAGGCGGCAAGGAAGTCGGCTCCAGCG
H.lupulus-HL.SW.V1.0.G020502.1                    GGAGGCAAGGCCCCAAGGAAGCAACTGGCAACCAAGGCCGCAAGGAAGTCTGCGCCGGCC

Marijuana-C32096195                               ------------------------------------------------------------
L.usitatissimum-LUS10013948                       ACCGGAGGAGTGAAGAAGCCCCACAGATTCAGGCCGGGAACCGTCGCTCTCCGTGAGATA
Textile-hemp-csa_locus_1853_iso_4_len_1208_ver_2  ACAGGAGGTGTGAAGAAGCCTCATCGGTTCAGGCCAGGAACTGTTGCTCTGAGAGAGATC
H.lupulus-HL.SW.V1.0.G020502.1                    ACCGGTGGTGTGAAGAAGCCTCATCGCTTCAGGCCTGGAACTGTGGCTCTGAGGGAGATC

Marijuana-C32096195                               ------------------------------------------------------------
L.usitatissimum-LUS10013948                       CGCAAGTACCAGAAGAGCACCGAGCTTCTGATCCGAAAGCTTCCCTTTCAGCGCCTCGTT
Textile-hemp-csa_locus_1853_iso_4_len_1208_ver_2  AGAAAGTACCAGAAGAGCACTGAGCTTCTCATCAGGAAGTTGCCTTTCCAGAGGCTTGTC
H.lupulus-HL.SW.V1.0.G020502.1                    AGGAAGTATCAGAAGAGCACTGAGCTTCTGATCAGGAAGTTGCCTTTCCAGAGGCTTGTG

Marijuana-C32096195                               ------------------------------------------------------------
L.usitatissimum-LUS10013948                       CGTGAGATCGCCCAGGATTTCAAGACAGATCTCAGGTTCCAGAGCTCCGCAGTGTCTGCT
Textile-hemp-csa_locus_1853_iso_4_len_1208_ver_2  AGGGAAATCGCTCAAGATTTCAAGACTGATCTTCGTTTCCAGAGCAGCGCCGTCTCCGCT
H.lupulus-HL.SW.V1.0.G020502.1                    AGGGAGATCGCTCAGGACTTCAAGACTGATCTTCGTTTCCAGAGCAGCGCCGTCTCGGCT

Marijuana-C32096195                               ------------------------------------------------------------
L.usitatissimum-LUS10013948                       CTACAGGAGGCAGCCGAGGCGTATCTCGTCGGACTGTTCGAGGATACTAACCTCTGCGCC
Textile-hemp-csa_locus_1853_iso_4_len_1208_ver_2  CTTCAAGAGGCCGCGGAGGCCTACTTGGTGGGTGTGTTTGAGGACACTAACCTCTGTGCC
H.lupulus-HL.SW.V1.0.G020502.1                    CTTCAGGAGGCGGCCGAGGCCTACTTGGTCGGACTCTTCGAGGACACCAACCTCTGCGCC

Marijuana-C32096195                               ---------------------------------------CAGCTAGCTCGAAGAATCAGG
L.usitatissimum-LUS10013948                       ATCCACGCCAAGAGGGTTACTATCATGCCCAAGGACATCCAGCTCGCAAGACGGATCAGA
Textile-hemp-csa_locus_1853_iso_4_len_1208_ver_2  ATTCATGCCAAGAGAGTCACTATCATGCCTAAAGATATCCAGCTAGCTCGAAGAATCAGG
H.lupulus-HL.SW.V1.0.G020502.1                    ATTCATGCCAAGAGGGTCACCATCATGCCTAAAGATATTCAGCTTGCTCGAAGAATCAGG
                                                                                         ***** **:.**.*.*****.

Marijuana-C32096195                               GGAGAGAGAGCTTAAGAAATGTAAACCTTTCTTTATTTAATTAGGATCTTTACCAAATAC
L.usitatissimum-LUS10013948                       GGCGAGCGCGCTTAG---------------------------------------------
Textile-hemp-csa_locus_1853_iso_4_len_1208_ver_2  GGAGAGAGAGCTTAAGAAATGTAAACCTTTCTTTATTTAATTAGGATCTTTAACAAATAC
H.lupulus-HL.SW.V1.0.G020502.1                    GGCGAGAGAGCTTAA---------------------------------------------
                                                  **.***.*.*****.

Marijuana-C32096195                               TT---------------------AGTAATCCAAGTAATATCTGTTGAAAACGAGTTTATA
L.usitatissimum-LUS10013948                       ------------------------------------------------------------
Textile-hemp-csa_locus_1853_iso_4_len_1208_ver_2  TTAAAATCATAGGCTTTGTGGAAAGTAATCCAAGTAATATATGTTGAAAACGAGTTTATA
H.lupulus-HL.SW.V1.0.G020502.1                    ------------------------------------------------------------

Marijuana-C32096195                               GTGATACATTAGGTTAGTAAAATGAGTCGAGTTCATTGTGAGAGGTTTCTCTCTAAATCT
L.usitatissimum-LUS10013948                       ------------------------------------------------------------
Textile-hemp-csa_locus_1853_iso_4_len_1208_ver_2  GTGATACATCAAGTTAGTAAAATGAGTCGAGTTCATTGTGAGAGGTTTCTCTCTAAATCT
H.lupulus-HL.SW.V1.0.G020502.1                    ------------------------------------------------------------

Marijuana-C32096195                               ATCTTGTGTTTCTTCAACAAATCTATAGAATCATGGTACGCACCGATCAAGGTTTATGTC
L.usitatissimum-LUS10013948                       ------------------------------------------------------------
Textile-hemp-csa_locus_1853_iso_4_len_1208_ver_2  ATCTTGTGTTCCTTCAACAAATCTATAGAATCATGGTACGCACTGATCAAGGTTTATGTT
H.lupulus-HL.SW.V1.0.G020502.1                    ------------------------------------------------------------

Marijuana-C32096195                               GAAATCACTGAAATTAAATTGTCAAACTACCGATGTTTCGCCGCTGAGATCGGGAAAGTT
L.usitatissimum-LUS10013948                       ------------------------------------------------------------
Textile-hemp-csa_locus_1853_iso_4_len_1208_ver_2  GAAATTACTGAAATTAAATTGCCAAACCACTGATGTTTCGCCGCTGAGATCGGGAAAGTT
H.lupulus-HL.SW.V1.0.G020502.1                    ------------------------------------------------------------

Marijuana-C32096195                               ACCGTCCGAATTAGTGAGGGTGAGACCCCATTGGGAAGAATGTGGATATTCTGAATCTCA
L.usitatissimum-LUS10013948                       ------------------------------------------------------------
Textile-hemp-csa_locus_1853_iso_4_len_1208_ver_2  ACCGTCCGAATTAGTGAGGGTGAGACCCCATTGGGAAGAATGTGGATATTCTGAATCTCA
H.lupulus-HL.SW.V1.0.G020502.1                    ------------------------------------------------------------

Marijuana-C32096195                               TCCAATT--------------------GTGACGGTAACTTTCCTGATCTTGGCGGCGAAA
L.usitatissimum-LUS10013948                       ------------------------------------------------------------
Textile-hemp-csa_locus_1853_iso_4_len_1208_ver_2  TCCAATTGGGTCTCACCCTCACTGGTTGTGACGGTAACTTTTCTGATCTCGGCGGCGAAA
H.lupulus-HL.SW.V1.0.G020502.1                    ------------------------------------------------------------
```

```
Marijuana-C32096195                              CATCGGTGGTTTGAAAATTTAATTTCAGTGATTTCGATATAAACTATGATCGGTGTCCAC
L.usitatissimum-LUS10013948                      ------------------------------------------------------------
Textile-hemp-csa_locus_1853_iso_4_len_1208_ver_2 CATCGGTGGTTTGAAAATTTAATTTCAGTAATTTTGATATAAACTATGATCGGTGTCTAC
H.lupulus-HL.SW.V1.0.G020502.1                   ------------------------------------------------------------

Marijuana-C32096195                              ATGATTCTATAGATTTGTTGAAGAAATAGGGGATAGATTTCGAGATAAACCTCCCCCACG
L.usitatissimum-LUS10013948                      ------------------------------------------------------------
Textile-hemp-csa_locus_1853_iso_4_len_1208_ver_2 ATGATTCTATAGATTTGTTGAAGAAATAGGAGATAGATTTCGAGATAAACCTCCCCCACG
H.lupulus-HL.SW.V1.0.G020502.1                   ------------------------------------------------------------

Marijuana-C32096195                              GAATCAACTTGACTCATTTTGTTAACTTGATGTATCACTATAGGCTCGTTTTCAACAGCA
L.usitatissimum-LUS10013948                      ------------------------------------------------------------
Textile-hemp-csa_locus_1853_iso_4_len_1208_ver_2 GAATCAACTTGGCTCATTTTGTTAACTTGATGTATCACTATAGGCTCGTTTTCAACAGCA
H.lupulus-HL.SW.V1.0.G020502.1                   ------------------------------------------------------------

Marijuana-C32096195                              ATATTACATGATTATTTTTTACAGCGCCTATGATTTTGGGTATTTGGTCGGTGTCTTGTT
L.usitatissimum-LUS10013948                      ------------------------------------------------------------
Textile-hemp-csa_locus_1853_iso_4_len_1208_ver_2 ATATTACATGATT-------ATTGCGCCTATGATTTTGGGTATTTGGTCGGTGTCTTGTT
H.lupulus-HL.SW.V1.0.G020502.1                   ------------------------------------------------------------

Marijuana-C32096195                              CTGTTTTTGTATCCCTTTGTGGGCAATAGTTGTTCATATCTGTTTAAAATCTAATGAGAA
L.usitatissimum-LUS10013948                      ------------------------------------------------------------
Textile-hemp-csa_locus_1853_iso_4_len_1208_ver_2 CTGTTTTTGTATCCCTTTGTGGGCAATAGTTGTTCATATCTGTTAAAAATCTAATGAGAA
H.lupulus-HL.SW.V1.0.G020502.1                   ------------------------------------------------------------

Marijuana-C32096195                              TGTTAGTTATAGCTATTAAGCTTCAAATCATATCTTGTTTGGTTAGCAGCTCTACTTAAA
L.usitatissimum-LUS10013948                      ------------------------------------------------------------
Textile-hemp-csa_locus_1853_iso_4_len_1208_ver_2 TGTTAGTTATAGCTATTAAGCTTCAAAAAAAA----------------------------
H.lupulus-HL.SW.V1.0.G020502.1                   ------------------------------------------------------------

Marijuana-C32096195                              CAAATACATTTTAAACCTAATATTTGTTGGTAATCAAACGACAATTGAGCTTTTATGAGA
L.usitatissimum-LUS10013948                      ------------------------------------------------------------
Textile-hemp-csa_locus_1853_iso_4_len_1208_ver_2 ------------------------------------------------------------
H.lupulus-HL.SW.V1.0.G020502.1                   ------------------------------------------------------------

Marijuana-C32096195                              TTCTGGC
L.usitatissimum-LUS10013948                      -------
Textile-hemp-csa_locus_1853_iso_4_len_1208_ver_2 -------
H.lupulus-HL.SW.V1.0.G020502.1                   -------
```

EF2

```
Textile-hemp-csa_locus_674_iso_1_len_2081_ver_2      AAAGATATAAACGATTACTCTATGAATGAAGTTAAAGAAAATATCTTTATGTTATTGTAA
Marijuana-C32108059                                  ------------------------------------------------------------
L.usitatissimum-Lus10031201                          ------------------------------------------------------------
H.lupulus-HL.SW.v1.0.G039978.1                       ------------------------------------------------------------

Textile-hemp-csa_locus_674_iso_1_len_2081_ver_2      CCATAATTAGAAAATCTGTAAGAACACCCCCAACAACAATAACAACAACAATTAGCATAA
Marijuana-C32108059                                  ------------------------------------------------------------
L.usitatissimum-Lus10031201                          ------------------------------------------------------------
H.lupulus-HL.SW.v1.0.G039978.1                       ------------------------------------------------------------

Textile-hemp-csa_locus_674_iso_1_len_2081_ver_2      GTTATTGACCTTTTTGGGAGTTTTCATCTTGAGACTGAGGTTGAGAAAGATGAGTGGTGT
Marijuana-C32108059                                  ------------------------------------------------------------
L.usitatissimum-Lus10031201                          ------------------------------------------------------------
H.lupulus-HL.SW.v1.0.G039978.1                       ------------------------------------------------------------

Textile-hemp-csa_locus_674_iso_1_len_2081_ver_2      AGCTTTTGATTCTCAAGGAGATGAGAAGACTTGAGAGAATGCCTAGGAGAGGTAAAACGT
Marijuana-C32108059                                  ------------------------------------------------------------
L.usitatissimum-Lus10031201                          ------------------------------------------------------------
H.lupulus-HL.SW.v1.0.G039978.1                       ------------------------------------------------------------

Textile-hemp-csa_locus_674_iso_1_len_2081_ver_2      AAGACCAAATAGAACTAGCTTTGTTGGCGGAGGCAACGGCTGCACCGGTGAGGCTGGCAA
Marijuana-C32108059                                  ------------------------------------------------------------
L.usitatissimum-Lus10031201                          ------------------------------------------------------------
H.lupulus-HL.SW.v1.0.G039978.1                       ------------------------------------------------------------

Textile-hemp-csa_locus_674_iso_1_len_2081_ver_2      TGGATGTGTTCTGCAAAATCATAGGAACACATCCAATCACAGTAGGCAGCGAAAAATCAA
Marijuana-C32108059                                  ------------------------------------------------------------
L.usitatissimum-Lus10031201                          ------------------------------------------------------------
H.lupulus-HL.SW.v1.0.G039978.1                       ------------------------------------------------------------

Textile-hemp-csa_locus_674_iso_1_len_2081_ver_2      CAAAGAAAGACTATGAAATGCAAAGAGAAAATCGGCAAAAAGCAAAGGGAAAACAAGATG
Marijuana-C32108059                                  ------------------------------------------------------------
L.usitatissimum-Lus10031201                          ------------------------------------------------------------
H.lupulus-HL.SW.v1.0.G039978.1                       ------------------------------------------------------------

Textile-hemp-csa_locus_674_iso_1_len_2081_ver_2      CTGAGCCTTCTAACTCTAAGCTTACTCTTTTTCTCTCTTTTGAGCCTTCCTTCTCAGGTG
Marijuana-C32108059                                  ------------------------------------------------------------
L.usitatissimum-Lus10031201                          ------------------------------------------------------------
H.lupulus-HL.SW.v1.0.G039978.1                       ------------------------------------------------------------

Textile-hemp-csa_locus_674_iso_1_len_2081_ver_2      TAATACTTTCGTTGGTGATACTCAAAAACTAGTCAAGATGGTGAAATTCACAGCAGAGGA
Marijuana-C32108059                                  ------------------------------------------------------------
L.usitatissimum-Lus10031201                          -------------------------------------ATGGTGAAGTTCACAGCTGAAGA
H.lupulus-HL.SW.v1.0.G039978.1                       ------------------------------------------------------------

Textile-hemp-csa_locus_674_iso_1_len_2081_ver_2      ATTGAGAAGAATTATGGACAACAAGCACAATATACGGAATATGTCCGTTATTGCCCATGT
Marijuana-C32108059                                  ------------------------------------------------------------
L.usitatissimum-Lus10031201                          GCTTCGTCGTATTATGGACTACAAGCACAACATCCGTAATATGTCCGTCATTGCCCATGT
H.lupulus-HL.SW.v1.0.G039978.1                       ------------------------------------------------------------

Textile-hemp-csa_locus_674_iso_1_len_2081_ver_2      TGATCATGGAAAATCAACTCTTACCGATTCTCTGGTGGCTGCTGCTGGTATCATTGCTCA
Marijuana-C32108059                                  --------GAAAATCAACTCTTACCGATTCTCTGGTGGCTGCTGCTGGTATCATTGCTCA
L.usitatissimum-Lus10031201                          TGATCACGGGAAATCTACACTTACTGATTCTCTAGTGGCTGCTGCGGGTATCATTGCCCA
H.lupulus-HL.SW.v1.0.G039978.1                       ------------------------------------------------------------

Textile-hemp-csa_locus_674_iso_1_len_2081_ver_2      AGAAGTTGCTGGTGATGTCCGGATGACTGATACTCGTCAGGATGAGGCAGAACGTGGCAT
Marijuana-C32108059                                  AGAAGTTGCTGGTGATGTCCGGATGACTGATACTCGTCAGGATGAGGCAGAACGTGGCAT
L.usitatissimum-Lus10031201                          AGAGGTTGCTGGGGATGTGCGGATGACTGATACCCGTGCTGATGAAGCAGAGCGTGGTAT
H.lupulus-HL.SW.v1.0.G039978.1                       ----------------------------------------------------------AT
                                                                                                               **

Textile-hemp-csa_locus_674_iso_1_len_2081_ver_2      TACAATTAAGTCTACTGGTATCTCTCTCTATTATGAGATGTCTGATGAATCTCTGAAGAG
Marijuana-C32108059                                  TACAATTAAGTCTACTGGTATCTCTCTCTATTATGAGATGTCTGATGAATCTCTGAAGAG
L.usitatissimum-Lus10031201                          TACAATCAAGTCTACTGGAATCTCTCTTTTCTATCAGATGACTGATGAGAGCATCAAGAG
H.lupulus-HL.SW.v1.0.G039978.1                       TACAATTAAGTCCACGGGTATCTCTCTCTATTATGAGATGTCTGATGAATCTCTCAAGAG
                                                     ****** ***** ** **:******** *: *** *****:*******.:  .* *****

Textile-hemp-csa_locus_674_iso_1_len_2081_ver_2      TTACGAAGGAAAGCGACAAGGGAATGAGTACCTCATCAATCTCATTGACTCACCTGGGCA
Marijuana-C32108059                                  TTACGAAGGAAAGCGACAAGGGAATGAGTACCTCATCAATCTCATTGACTCACCTGGGCA
L.usitatissimum-Lus10031201                          TTACACGGGAAAGAGGGACGGGAGTGAGTACCTTATCAATCTCATCGACTCCCCTGGGCA
H.lupulus-HL.SW.v1.0.G039978.1                       TTATAAAGGAGAGAGACAAGGGAATGAGTACCTCATCAATCTCATTGATTCACCTGGGCA
                                                     *** ...***.**.*. *.****.********* *********** ** **.********

Textile-hemp-csa_locus_674_iso_1_len_2081_ver_2      TGTTGACTTTTCATCAGAAGTCACTGCTGCTCTTCGCATCACTGATGGTGCACTTGTGGT
Marijuana-C32108059                                  TGTTGACTTTTCATCAGAAGTCACTGCTGCTCTTCGtATCACTGATGGTGCACTTGTGGT
L.usitatissimum-Lus10031201                          CGTTGACTTCTCATCTGAAGTCACTGCTGCTCTCCGTATCACTGATGGTGCTCTTGTGGT
H.lupulus-HL.SW.v1.0.G039978.1                       TGTTGACTTCTCATCAGAAGTCACTGCTGCTCTTCGTATCACTGATGGTGCACTTGTTGT
                                                      ******** *****:***************** ** **************:***** **

Textile-hemp-csa_locus_674_iso_1_len_2081_ver_2      TGTTGATTGTATTGAGGGTGTCTGTGTCCAAACAGAGACTGTGCTTCGTCAAGCCCTTGG
Marijuana-C32108059                                  TGTTGATTGTATTGAGGGTGTCTGTGTCCAAACAGAGACTGTGCTTCGTCAAGCCCTTGG
L.usitatissimum-Lus10031201                          CGTGGATTGTGTTGAGGGGGTCTGGGTGCAGACTGAGACTGTTCTTCGTCAAGCTTTGGG
H.lupulus-HL.SW.v1.0.G039978.1                       GGTTGATTGTATTGAGGGTGTTTGTGTCCAAACAGAAACTGTGCTCCGTCAAGCCCTTGG
                                                      ** ******.******* ** ** ** **.**:**.***** ** ********  * **

Textile-hemp-csa_locus_674_iso_1_len_2081_ver_2      TGAAAGGATTAGGCCTGTGTTAACTGTTAACAAGATGGACAGGTGTTTTCTTGAGCTGCA
Marijuana-C32108059                                  TGAAAGGATTAGGCCTGTGTTAACTGTTAACAAGATGGACAGGTGTTTTCTTGAGCTGCA
L.usitatissimum-Lus10031201                          TGAAAGGATCAGGCCTGTGCTCACTGTGAACAAGATGGACAGGTGTTTCCTTGAGCTCCA
H.lupulus-HL.SW.v1.0.G039978.1                       TGAAAGGATTAGGCCTGTGTTGACTGTTAACAAGATGGACAGGTGTTTCCTTGAGCTGCA
                                                     ********* ********* * ***** ******************** ******** **
```

```
Textile-hemp-csa_locus_674_iso_1_len_2081_ver_2    AGTTGATGGGGAAGAAGCTTACCAAACTTTCCAGAGAGTTATTGAGAATGCTAATGTGAT
Marijuana-C32108059                                AGTTGATGGGGAAGAAGCTTACCAAACTTTCCAGAGAGTTATTGAGAATGCTAATGTGAT
L.usitatissimum-Lus10031201                        AGTGGATGGAGAGGAAGCCTATCAGACGTTCCAGAGGGTTATTGAGAATGCTAATGTCAT
H.lupulus-HL.SW.v1.0.G039978.1                     GGTTGATGGGGAGGAAGCTTACCAAACTTTCCAGAGAGTCATTGAGAATGCTAATGTGAT
                                                   .** *****.**.***** ** **.** ********.** ***************** **

Textile-hemp-csa_locus_674_iso_1_len_2081_ver_2    TATGGCTACCTATGAAGATCCTCTTCTTGGTGATGTTCAGGTCTATCCTGAGAAAGGCAC
Marijuana-C32108059                                TATGGCTACCTATGAAGATCCTCTTCTTGGTGATGTTCAGGTCTATCCTGAGAAAGGCAC
L.usitatissimum-Lus10031201                        CATGGCAACCTATGAAGATCCTCTTCTCGGTGATGTCCAGGTGTACCCGGAGAAAGGAAC
H.lupulus-HL.SW.v1.0.G039978.1                     TATGGCTACCTATGAAGATCCTCTTCTTGGTGATGTTCAGGTCTATCCTGAGAAAGGAAC
                                                    *****:******************** ******** ***** ** ** ********.**

Textile-hemp-csa_locus_674_iso_1_len_2081_ver_2    TGTAGCTTTCTCTGCTGGTTTGCATGGTTGGGCTTTTACTCTGACTAACTTTGCCAAGAT
Marijuana-C32108059                                TGTAGCTTTCTCTGCTGGTTTGCATGGTTGGGCTTTTACTCTGACTAACTTTGCCAAGAT
L.usitatissimum-Lus10031201                        TGTTGCCTTTTCCGCTGGTCTGCATGGATGGGCTTTTACGTTGACCAACTTTGCCAAGAT
H.lupulus-HL.SW.v1.0.G039978.1                     AGTAGCTTTCTCTGCTGGTTTGCATGGTTGGGCGTTTACTCTGACTAACTTTGCCAAGAT
                                                   :**:** ** ** ****** *******:***** *****  **** **************

Textile-hemp-csa_locus_674_iso_1_len_2081_ver_2    GTATGCCTCCAAGTTTGGTGTTGATGAGTCAAAGATGATGGAGCGTCTCTGGGGTGAAAA
Marijuana-C32108059                                GTATGCCTCCAAGTTTGGTGTTGATGAGTCAAAGATGATGGAGCGTCTCTGGGGTGAAAA
L.usitatissimum-Lus10031201                        GTATGCTTCTAAGTTTGGAGTGGACGAGGCCAAGATGATGGAAAGACTGTGGGGTGAGAA
H.lupulus-HL.SW.v1.0.G039978.1                     GTATGCCTCCAAGTTTGGTGTCGATGAGTCAAAGATGATGGAGCGTCTCTGGGGTGAAAA
                                                   ****** ** ********:** ** *** *.***********..*:** ********.**

Textile-hemp-csa_locus_674_iso_1_len_2081_ver_2    CTTCTTTGATCCTGCTACTAAAAAATGGACCACCAAGAATACTGGATCTCCTACTTGCAA
Marijuana-C32108059                                CTTCTTTGATCCTGCTACTAAAAAATGGACCACCAAGAATACTGGATCTCCTACTTGCAA
L.usitatissimum-Lus10031201                        TTTCTTTGACCCTGCCACTAAGAAATGGACCACCAAGAACACTGGCTCTGCTACTTGCAA
H.lupulus-HL.SW.v1.0.G039978.1                     CTTCTTTGATCCTGCTACCAAGAAATGGACCAGCAAGAACACTGGATCTCCCAGCTGCAA
                                                    ******** ***** ** **.********** ****** *****.*** * *  *****

Textile-hemp-csa_locus_674_iso_1_len_2081_ver_2    GCGTGGTTTTGTTTTGTTCTGTTATGAACCTATCAAGCAGATTATTAGCTCTTGTATGAA
Marijuana-C32108059                                GCGTGGTTTTGTTTTGTTCTGTTATGAACCTATCAAGCAGATTATTAGCTCTTGTATGAA
L.usitatissimum-Lus10031201                        GCGTGGGTTTGTGCAGTTCTGTTACGAGCCGATCAAGCAGATCATCAACACCTGTATGAC
H.lupulus-HL.SW.v1.0.G039978.1                     GCGTGGTTTTGTTATGTTCTGTTATGAACCTATCAAGCAGATTATTAATACTTGTATGAA
                                                   ****** *****  :********* **.** *********** ** *. :* *******.

Textile-hemp-csa_locus_674_iso_1_len_2081_ver_2    TGACCAAAAGGATAAGTTGTGGCCAATGCTACAGAAGCTTAATGTCACCATGAAGTCTGA
Marijuana-C32108059                                TGACCAAAAGGATAAGTTGTGGCCAATGCTACAGAAGCTTAATGTCACCATGAAGTCTGA
L.usitatissimum-Lus10031201                        CGACCAGAAGGACAAACTGTGGCCCATGTTGAAAAAACTCAATGTTACAATGAAGGGTGA
H.lupulus-HL.SW.v1.0.G039978.1                     TGACCAAAAGAATAAGCTGTGGCCTATGCTACAGAAGCTAGGTGTCACCATGAAGTCTGA
                                                    *****.***.* **. ******* *** *..*.**.** ..*** **.******  ***

Textile-hemp-csa_locus_674_iso_1_len_2081_ver_2    TGAGAAAGAACTGTTGGGAAAGGCATTGATGAAGCGTGTCATGCAAACATGGCTTCCTGC
Marijuana-C32108059                                TGAGAAAGAACTGTTGGGAAAGGCATTGATGAAGCGTGTCATGCAAACATGGCTTCCTGC
L.usitatissimum-Lus10031201                        AGAGAAGGACTTAATGGGCAAGGCTTTGATGAAGCGTGTCATGCAAACCTGGCTTCCAGC
H.lupulus-HL.SW.v1.0.G039978.1                     TGAGAAAGAACTGATGGGAAAGGCATTGATGAAGCGAGTCATGCAAACATGGCTTCCTGC
                                                   :*****.**. *.:****.*****:***********:***********.********:**

Textile-hemp-csa_locus_674_iso_1_len_2081_ver_2    CAGTGCTGCTCTCTTGGAAATGATGATCTTTCATCTTCCTTCCCCTGCAACGGCTCAGAA
Marijuana-C32108059                                CAGTGCTGCTCTCTTGGAAATGATGATCTTTCATCTTCCTTCCCCTGCAACGGCTCAGAA
L.usitatissimum-Lus10031201                        TGCAGATGCTCTCCTGGAAATGATGATCTTTCACCTTCCGTCTCCTGCTACTGCCCAGAA
H.lupulus-HL.SW.v1.0.G039978.1                     TAGTACTGCTCTCTTGGAAATGATGATCTTTCATCTTCCTTCCCCTGCAACGGCTCAGAA
                                                    . :..******* ******************* ***** ** *****:** ** *****

Textile-hemp-csa_locus_674_iso_1_len_2081_ver_2    ATATCGTGTTGAGAACCTGTATGAAGGTCCTCTTGATGATGTTTACGCAACAGCTATCAG
Marijuana-C32108059                                ATATCGTGTTGAGAACCTGTATGAAGGTCCTCTTGATGATGTTTACGCAACAGCTATCAG
L.usitatissimum-Lus10031201                        GTACCGTGTGGAGAACTTGTATGAGGGCCCACTTGATGATCAGTATGCTAACGCCATCAG
H.lupulus-HL.SW.v1.0.G039978.1                     ATATCGTGTGGAGAACTTGTATGAAGGTCCTCTTGATGATATATACGCAACTGCTATCAG
                                                   .** ***** ****** *******.** **:********* : ** **:*. ** *****

Textile-hemp-csa_locus_674_iso_1_len_2081_ver_2    GAACTGTGATCCAAATGGACCCCTTATGCTCTATGTATCAAAGATGATTCCAGCATCTGA
Marijuana-C32108059                                GAACTGTGATCCAAATGGACCCCTTATGCTCTATGTATCAAAGATGATTCCAGCATCTGA
L.usitatissimum-Lus10031201                        GAACTGTGACCCTGATGGCCCGCTCATGCTTTATGTATCCAAAATGATTCCCGCTTCTGA
H.lupulus-HL.SW.v1.0.G039978.1                     GAACTGTGATCCTAATGGACCCCTTATGCTCTATGTATCAAAGATGATTCCTGCATCCGA
                                                   ********* **:.****.** ** ***** ********.**.******** **:** **

Textile-hemp-csa_locus_674_iso_1_len_2081_ver_2    TAAGGGTAGGTTCTTTGCCTTTGGTCGTGTCTTTGCAGGCAAGGTCTCTACTGGTATGAA
Marijuana-C32108059                                TAAGGGTAGGTTCTTTGCCTTTGGTCGTGTCTTTGCAGGCAAGGTCTCTACTGGTATGAA
L.usitatissimum-Lus10031201                        CAAGGGTAGGTTCTTTGCCTTTGGTCGTGTCTTTGCTGGTAAGGTATCTACTGGTGTGAA
H.lupulus-HL.SW.v1.0.G039978.1                     TAAGGGCAGGTTCTTTGCCTTTGGTCGTGTCTTTGCAGGCAAGGTCGATACTGGTATGAA
                                                    ***** *****************************:** *****. .*******.****

Textile-hemp-csa_locus_674_iso_1_len_2081_ver_2    GGTTAGGATTATGGGTCCAAACTATGTTCCTGGAGAGAAGAAAGATTTGTATGTGAAGAG
Marijuana-C32108059                                GGTTAGGATTATGGGTCCAAACTATGTTCCTGGAGAGAAGAAAGATTTGTATGTGAAGAG
L.usitatissimum-Lus10031201                        GGTGAGGATTATGGGTCCCAACTACGTCCCTGGTCAGAAGAAGGATCTGTATGTGAAGAA
H.lupulus-HL.SW.v1.0.G039978.1                     GGTTAGGATTATGGGTCCAAACTTTGTTCCTGGAGAGAAGAAAGATTTGTATGTGAAGAG
                                                   *** **************.****: ** *****: *******.*** ************.

Textile-hemp-csa_locus_674_iso_1_len_2081_ver_2    TGTTCAGAGAACTGTCATTTGGATGGGAAAGAGACAAGAAACAGTTGAGGATGTTCCTTG
Marijuana-C32108059                                TGTTCAGAGAACTGTCATTTGGATGGGAAAGAGACAAGAAACAGTTGAGGATGTTCCTTG
L.usitatissimum-Lus10031201                        TGTCCAGAGAACTGTTATCTGGATGGGTAAGAGGCAGGAGACTGTGGAGGATGTTCCTTG
H.lupulus-HL.SW.v1.0.G039978.1                     TGTTAAGAGAACTGTCATTTGGATGGGAAAGAGACAAGAAACAGTTGAGGATGTTCCATG
                                                   *** .********** ** ********:*****.**.**.**:** ***********:**

Textile-hemp-csa_locus_674_iso_1_len_2081_ver_2    TGGTAATACAGTTGCTATGGTTGGTTTGGATCAGTTCATTACCAAGAATGCAACTTTGAC
Marijuana-C32108059                                TGGTAATACAGTTGCTATGGTTGGTTTGGATCAGTTCATTACCAAGAATGCAACTTTGAC
L.usitatissimum-Lus10031201                        TGGTAACACCGTTGCTTTGGTTGGTTTGGATCAATTCATCACAAAGAATGCCACTTTGAC
H.lupulus-HL.SW.v1.0.G039978.1                     TGGTAATACAGTTGCTATGGTTGGTTTGGATCAGTTCATTACCAAGAATGCAACTTTGAC
                                                   ****** **.******:****************.***** **.********.********

Textile-hemp-csa_locus_674_iso_1_len_2081_ver_2    AAATGAAAAGGAAGTTGATGCTCATCCTATCCGAGCCATGAAATTTTCAGTCTCCCCTGT
Marijuana-C32108059                                AAATGAAAAGGAAGTTGATGCTCATCCTATCCGAGCCATGAAATTTTCAGTCTCCCCTGT
L.usitatissimum-Lus10031201                        CAACGAGAAGGAAGTTGATGCTCACCCAATCCGTGCAATGAAGTTCTCAGTATCCCCTGT
H.lupulus-HL.SW.v1.0.G039978.1                     AAATGAAAAGGAAGTCGATGCTCATCCTATCCGAGCTATGAAGTTTTCGGTCTCCCCTGT
                                                   .** **.******** ******** **:*****:** *****.** **.**.********

Textile-hemp-csa_locus_674_iso_1_len_2081_ver_2    TGTGCGTGTGGCCGTGCAATGCAAAGTTGCATCTGACCTTCCCAAACTTGTTGAAGGTCT
Marijuana-C32108059                                TGTGCGTGTGGCCGTGCAATGCAAAGTTGCATCTGACCTTCCCAAACTTGTTGAAGGTCT
L.usitatissimum-Lus10031201                        TGTTCGTGTTGCTGTTCAGTGCAAGGTTGCTTCTGATCTGCCGAAGTTGGTGGAAGGTTT
H.lupulus-HL.SW.v1.0.G039978.1                     TGTGCGTGTGGCCGTGCAATGCAAAGTTGCATCCGACCTTCCCAAACTTGTTGAAGGTCT
                                                   *** ***** ** ** **.*****.*****:** ** ** ** **. * ** ****** *

Textile-hemp-csa_locus_674_iso_1_len_2081_ver_2    GAAACGTCTGGCCAAGTCAGATCCTATGGTGGTATGTTCTA-------------------
Marijuana-C32108059                                GAAACGTCTGGCCAAGTCAGATCCTATGGTGGTATGTTCTA-------------------
L.usitatissimum-Lus10031201                        GAAACGTCTGGCCAAGTCTGACCCCATGGTTGTGTGTAGTATTGAAGAGTCTGGTGAGCA
```

```
H.lupulus-HL.SW.v1.0.G039978.1                GAAACGTTTAGCTAAGTCAGATCCTATGGTGGTGTGTTCTA-------------------
                                              ******* *.** *****:** ** ***** **.***: **

Textile-hemp-csa_locus_674_iso_1_len_2081_ver_2  ------------------------------------------------------------
Marijuana-C32108059                              ------------------------------------------------------------
L.usitatissimum-Lus10031201                      CATCATTGCTGGTGCTGGAGAGCTTCACCTTGAAATCTGTTTGAAGGATCTTGTGGATGA
H.lupulus-HL.SW.v1.0.G039978.1                   ------------------------------------------------------------

Textile-hemp-csa_locus_674_iso_1_len_2081_ver_2  ------------------------------------------------------------
Marijuana-C32108059                              ------------------------------------------------------------
L.usitatissimum-Lus10031201                      CTTCATGGGTGGTGCTGAGATCACGAAATCCGACCCAGTTGTGTCCTTCCGTGAAACCGT
H.lupulus-HL.SW.v1.0.G039978.1                   ------------------------------------------------------------

Textile-hemp-csa_locus_674_iso_1_len_2081_ver_2  ------------------------------------------------------------
Marijuana-C32108059                              ------------------------------------------------------------
L.usitatissimum-Lus10031201                      CCTTGAGAAATCAAGCAGAGTCGTCATGAGCAAGTCCCCCAACAAGCATAACCGTCTCTA
H.lupulus-HL.SW.v1.0.G039978.1                   ------------------------------------------------------------

Textile-hemp-csa_locus_674_iso_1_len_2081_ver_2  ------------------------------------------------------------
Marijuana-C32108059                              ------------------------------------------------------------
L.usitatissimum-Lus10031201                      CATGGAGGCCAGACCATTGGAGGAAGGATTAGCTGAGGCAATCGATGATGGCCGCATTGG
H.lupulus-HL.SW.v1.0.G039978.1                   ------------------------------------------------------------

Textile-hemp-csa_locus_674_iso_1_len_2081_ver_2  ------------------------------------------------------------
Marijuana-C32108059                              ------------------------------------------------------------
L.usitatissimum-Lus10031201                      CCCAAGGGATGACCCCAAGGTCCGTTCCAAGATCCTCTCTGAGGAGTTCGGCTGGGACAA
H.lupulus-HL.SW.v1.0.G039978.1                   ------------------------------------------------------------

Textile-hemp-csa_locus_674_iso_1_len_2081_ver_2  ------------------------------------------------------------
Marijuana-C32108059                              ------------------------------------------------------------
L.usitatissimum-Lus10031201                      GGATCTCGCCAAGAAGATCTGGTGCTTCGGTCCCGAGACCACCGGCCCTAACATGGTAGT
H.lupulus-HL.SW.v1.0.G039978.1                   ------------------------------------------------------------

Textile-hemp-csa_locus_674_iso_1_len_2081_ver_2  ------------------------------------------------------------
Marijuana-C32108059                              ------------------------------------------------------------
L.usitatissimum-Lus10031201                      GGACATGTGTAAGGGAGTTCAGTACCTGAATGAAATCAAGGATTCAGTCGTGGCTGGGTT
H.lupulus-HL.SW.v1.0.G039978.1                   ------------------------------------------------------------

Textile-hemp-csa_locus_674_iso_1_len_2081_ver_2  ------------------------------------------------------------
Marijuana-C32108059                              ------------------------------------------------------------
L.usitatissimum-Lus10031201                      CCAGTGGGCATCAAAGGAAGGTGCGCTTGCAGAAGAAAACATGAGAGGAATCTGCTTCGA
H.lupulus-HL.SW.v1.0.G039978.1                   ------------------------------------------------------------

Textile-hemp-csa_locus_674_iso_1_len_2081_ver_2  ------------------------------------------------------------
Marijuana-C32108059                              ------------------------------------------------------------
L.usitatissimum-Lus10031201                      AGTCTGTGATGTGGTTCTCCACGCCGATGCTATCCACAGAGGTGGTGGTCAAGTCATCCC
H.lupulus-HL.SW.v1.0.G039978.1                   ------------------------------------------------------------

Textile-hemp-csa_locus_674_iso_1_len_2081_ver_2  ------------------------------------------------------------
Marijuana-C32108059                              ------------------------------------------------------------
L.usitatissimum-Lus10031201                      AACAGCCAGGAGGGTCATCTACGCTTCCCCACTGTCTGCCCAGCCAAGGCTGGTCGAGCC
H.lupulus-HL.SW.v1.0.G039978.1                   ------------------------------------------------------------

Textile-hemp-csa_locus_674_iso_1_len_2081_ver_2  ------------------------------------------------------------
Marijuana-C32108059                              ------------------------------------------------------------
L.usitatissimum-Lus10031201                      TTGCTACCCTCGCGGGNNNNNNNNNNNNNNNNNNNNNNNNNNNNNNNNNNNNNNNNNNNN
H.lupulus-HL.SW.v1.0.G039978.1                   ------------------------------------------------------------

Textile-hemp-csa_locus_674_iso_1_len_2081_ver_2  ------------------------------------------------------------
Marijuana-C32108059                              ------------------------------------------------------------
L.usitatissimum-Lus10031201                      NNNNNNNNNNNNNNNNNNNNNNNNNNNNNNNNNNNNNNNNNNNNNNNNNNNNNNNNNNNN
H.lupulus-HL.SW.v1.0.G039978.1                   ------------------------------------------------------------

Textile-hemp-csa_locus_674_iso_1_len_2081_ver_2  ------------------------------------------------------------
Marijuana-C32108059                              ------------------------------------------------------------
L.usitatissimum-Lus10031201                      NNNNNNNNNNNNNNNNNAAGAGGAAGGGTTTGAAGGAGCAGATGACCCCCCTCTCCGATT
H.lupulus-HL.SW.v1.0.G039978.1                   ------------------------------------------------------------

Textile-hemp-csa_locus_674_iso_1_len_2081_ver_2  ------------------------------------------------------------
Marijuana-C32108059                              ------------------------------------------------------------
L.usitatissimum-Lus10031201                      TCGAGGACAAGCTGTAAGAGCATCTTTTGGTCTTTGCTGCAGGAGATGGATGATCCTAGT
H.lupulus-HL.SW.v1.0.G039978.1                   ------------------------------------------------------------

Textile-hemp-csa_locus_674_iso_1_len_2081_ver_2  -------------------------------------------------------
Marijuana-C32108059                              -------------------------------------------------------
L.usitatissimum-Lus10031201                      TATGGTTTTTTTTATACTGATGTTGTGTCGTTGTGGTTTGAGTCCTATTATGTGA
H.lupulus-HL.SW.v1.0.G039978.1                   -------------------------------------------------------
```

Actin

```
L.usitatissimum-Lus10001694                           ------------------------------------------------------------
Textile-hemp-csa_locus_2263_iso_5_len_1488_ver_2      ---------CTTCTTCTCTCTCAAACCTCTA---AACTCTAAACCATTCATTCTTTCATT
Marijuana-scaffold122787                              TGTTGCATTCTTAGTATCATTGT-ATCGCTATATGACGATCTACCGCTTTT--ATTTATT
H.lupulus-HL.SW.v1.0.G017631.1                        ------------------------------------------------------------

L.usitatissimum-Lus10001694                           ------------------------------------------------------------
Textile-hemp-csa_locus_2263_iso_5_len_1488_ver_2      CATTTACCACAAACCCTCTAAACTTCCATCTTGCTTTTCGCATTTCAACAGGTAAGTGAA
Marijuana-scaffold122787                              ATTTTATGAA-----AGATGTTCTTACAGCATAG-TTTGGTGAACAAATAGGTAAGTGAA
H.lupulus-HL.SW.v1.0.G017631.1                        ------------------------------------------------------------

L.usitatissimum-Lus10001694                           -ATGGCAGAAGGTGAGGATATCCAGCCCCTCGTCTGCGACAATGGAACAGGAATGGTCA-
Textile-hemp-csa_locus_2263_iso_5_len_1488_ver_2      GATGGCAGATGCAGAGGATATTCAGCCACTTGTCTGCGATAATGGAACTGGAATGGTCA-
Marijuana-scaffold122787                              GATGGCAGATGCAGAGGATATTCAGCCACTTGTCTGCGATAATGGAACTGGAATGGTCAA
H.lupulus-HL.SW.v1.0.G017631.1                        ------------------------------------------------------------

L.usitatissimum-Lus10001694                           ------------------------------------------------------------
Textile-hemp-csa_locus_2263_iso_5_len_1488_ver_2      ------------------------------------------------------------
Marijuana-scaffold122787                              GGTATTAATGTTATTTGTCATTGTTTAAAAGTTTCCCATTGAATCATTAATCTTCTGTGC
H.lupulus-HL.SW.v1.0.G017631.1                        ------------------------------------------------------------

L.usitatissimum-Lus10001694                           ------------------------------------------------------------
Textile-hemp-csa_locus_2263_iso_5_len_1488_ver_2      ------------------------------------------------------------
Marijuana-scaffold122787                              AAAAGCTAGCTTAATGTTACTCGTGGGTGTGTAGAATTTGCCTCTTGACCATGAATTATT
H.lupulus-HL.SW.v1.0.G017631.1                        ------------------------------------------------------------

L.usitatissimum-Lus10001694                           ---------------AGGCTGGATTTGCTGGAGATGATGCCCCTCGTGCTGTGTTCCCCA
Textile-hemp-csa_locus_2263_iso_5_len_1488_ver_2      ---------------AGGCTGGGTTTGCTGGAGATGATGCTCCACGAGCTGTGTTCCCCA
Marijuana-scaffold122787                              GTGATCTTACCTATTAGGCTGGGTTTGCTGGAGATGATGCTCCACGAGCTGTGTTCCCCA
H.lupulus-HL.SW.v1.0.G017631.1                        ------------------------------------------------------------

L.usitatissimum-Lus10001694                           GTATTGTTGGTCGTCCACGTCACACTGGCGTGATGGTTGGAATGGGTCAGAAAGATGCCT
Textile-hemp-csa_locus_2263_iso_5_len_1488_ver_2      GTATCGTGGGTCGTCCTCGTCACACTGGTGTAATGGTTGGAATGGGCCAGAAAGACGCAT
Marijuana-scaffold122787                              GTATCGTGGGTCGTCCTCGTCACACTGGTGTAATGGTTGGAATGGGCCAGAAAGACGCAT
H.lupulus-HL.SW.v1.0.G017631.1                        ------------------------------------------------------------

L.usitatissimum-Lus10001694                           ATGTGGGTGATGAAGCCCAGTCCAAGAGAGGTATTTTGACATTGAAATACCCAATTGAAC
Textile-hemp-csa_locus_2263_iso_5_len_1488_ver_2      ATGTGGGTGATGAGGCACAATCCAAGCGAGGTATCTTAACTCTGAAGTACCCAATTGAGC
Marijuana-scaffold122787                              ACGTGGGTGATGAAGCACAATCCAAGCGAGGTATCTTAACTCTGAAGTACCCAATTGAGC
H.lupulus-HL.SW.v1.0.G017631.1                        ------------------------------------------------------------

L.usitatissimum-Lus10001694                           ATGGTATTGTGAACAATTGGGATGACATGGAAAAGATTTGGCATCACACCTTCTACAACG
Textile-hemp-csa_locus_2263_iso_5_len_1488_ver_2      ATGGTATTGTCAGCAACTGGGATGACATGGAAAAGATCTGGCATCACACTTTTTACAATG
Marijuana-scaffold122787                              ATGGTATTGTCAGCAACTGGGATGACATGGAAAAGATCTGGCATCACACTTTTTACAATG
H.lupulus-HL.SW.v1.0.G017631.1                        ------------------------------------------------------------

L.usitatissimum-Lus10001694                           AGCTTCGTGTTGCTCCGGAAGAGCACCCTGTTCTTCTGACTGAGGCTCCTCTGAACCCCA
Textile-hemp-csa_locus_2263_iso_5_len_1488_ver_2      AGCTTCGTGTTGCCCCTGAGGAACACCCCGTTCTTCTAACCGAAGCTCCACTTAACCCCA
Marijuana-scaffold122787                              AGCTTCGTGTTGCCCCTGAGGAACACCCCGTTCTTCTAACCGAAGCTCCACTTAACCCCA
H.lupulus-HL.SW.v1.0.G017631.1                        ------------------------------------------------------------

L.usitatissimum-Lus10001694                           AGGCAAATCGTGAGAAGATGACCCAGATCATGTTTGAGACCTTCAATACCCCTGCCATGT
Textile-hemp-csa_locus_2263_iso_5_len_1488_ver_2      AGGCCAATCGTGAAAAAATGACCCAGATCATGTTTGAGACCTTTAACACCCCTGCTATGT
Marijuana-scaffold122787                              AGGCCAATCGTGAAAAAATGACCCAGATCATGTTTGAGACCTTTAACACTCCTGCTATGT
H.lupulus-HL.SW.v1.0.G017631.1                        ------------------------------------------------------------

L.usitatissimum-Lus10001694                           ATGTTGCTATTCAGGCTGTATTGTCACTGTATGCCAGTGGTCGTACAACTGGT-------
Textile-hemp-csa_locus_2263_iso_5_len_1488_ver_2      ATGTTGCCATTCAAGCCGTTCTTTCTCTGTATGCCAGTGGTCGTACTACCGGT-------
Marijuana-scaffold122787                              ATGTTGCCATTCAAGTCGTTCTTTCTCTGTATGCCAGTGGTCGTACTACCGGTGAGTACC
H.lupulus-HL.SW.v1.0.G017631.1                        ------------------------------------------------------------

L.usitatissimum-Lus10001694                           ------------------------------------------------------------
Textile-hemp-csa_locus_2263_iso_5_len_1488_ver_2      ------------------------------------------------------------
Marijuana-scaffold122787                              AAGTACTTTCTTTTCTTCTTTTCATTGTCGTAGCATGATTTCTTGTTAGAGTATCCTATA
H.lupulus-HL.SW.v1.0.G017631.1                        ------------------------------------------------------------

L.usitatissimum-Lus10001694                           ------------------------------------------------------------
Textile-hemp-csa_locus_2263_iso_5_len_1488_ver_2      ------------------------------------------------------------
Marijuana-scaffold122787                              GATACTGAAATGAGTCTTTAAATATGGTAAGAACTGAGATTTTACTAATACCAATCTCAT
H.lupulus-HL.SW.v1.0.G017631.1                        ------------------------------------------------------------

L.usitatissimum-Lus10001694                           ------------------------------------------------------------
Textile-hemp-csa_locus_2263_iso_5_len_1488_ver_2      ------------------------------------------------------------
Marijuana-scaffold122787                              TCTGTTTACCAGCTACTTGCATCATGATATGATAGTTTCTTACCTTGTCAAATATTGTAG
H.lupulus-HL.SW.v1.0.G017631.1                        ------------------------------------------------------------

L.usitatissimum-Lus10001694                           ------------------------------------------------------------
Textile-hemp-csa_locus_2263_iso_5_len_1488_ver_2      ------------------------------------------------------------
Marijuana-scaffold122787                              TGTGGCTTTGGTTTTGGAAATTATATTTTTCTTGAAGTTTAAACCTTGACCCTCATTTTC
H.lupulus-HL.SW.v1.0.G017631.1                        ------------------------------------------------------------

L.usitatissimum-Lus10001694                           ------------------------------------------------------------
Textile-hemp-csa_locus_2263_iso_5_len_1488_ver_2      ------------------------------------------------------------
Marijuana-scaffold122787                              TAATACTATTATGTAGCTTTTATATTGCCCTGTCTTAAAATAATTTAGCTAGAAGTTTTG
H.lupulus-HL.SW.v1.0.G017631.1                        ------------------------------------------------------------
```

```
L.usitatissimum-Lus10001694                           ----------------ATCGTGCTAGATTCTGGTGATGGTGTTAGCCACACAGTCCCCAT
Textile-hemp-csa_locus_2263_iso_5_len_1488_ver_2      ----------------ATTGTGCTGGATTCTGGTGATGGTGTAAGCCATACTGTCCCCAT
Marijuana-scaffold122787                              CCTTAGTTTTGCAGGTATTGTGCTGGATTCTGGTGATGGTGTAAGCCATACTGTCCCCAT
H.lupulus-HL.SW.v1.0.G017631.1                        -------------GGTATTGTGTTGGACTCTGGTGATGGTGTCAGCCATACTGTCCCTAT
                                                                      ** *** *.** ************** ***** **:***** **

L.usitatissimum-Lus10001694                           CTACGAGGGTTACGCTCTCCCTCATGCCATCCTTCGTCTCGACCTTGCTGGGCGTGACCT
Textile-hemp-csa_locus_2263_iso_5_len_1488_ver_2      ATACGAGGGATATGCTCTTCCTCATGCCATTCTACGTCTTGATCTTGCTGGTCGTGATCT
Marijuana-scaffold122787                              ATACGAGGGGTATGCTCTTCCTCATGCCATTCTACGTCTTGATCTTGCTGGTCGTGATCT
H.lupulus-HL.SW.v1.0.G017631.1                        ATACGAGGGATATGCCCTCCCTCATGCCATTCTCCGTCTTGACCTTGCTGGTCGTGATCT
                                                      .******** ** ** ** *********** ** ***** ** ******** ***** **

L.usitatissimum-Lus10001694                           TACAGATAGCTTAATGAAGATCCTCACCGAGCGGGGTTATTCTTTTACCACTACAGCAGA
Textile-hemp-csa_locus_2263_iso_5_len_1488_ver_2      TACTGATCACCTAATGAAGATTCTCACCGAGCGTGGATACTCCTTCACCACCACTGCTGA
Marijuana-scaffold122787                              TACTGATCACCTAATGAAGATTCTCACCGAGCGTGGATACTCCTTCACCACCACTGCTGA
H.lupulus-HL.SW.v1.0.G017631.1                        TACTGATCACTTAATGAAGATTCTTACTGAGCGTGGGTACTCCTTCACCACTACTGCTGA
                                                      ***:***..* ********** ** ** ***** ** ** ** ** ***** **:**:**

L.usitatissimum-Lus10001694                           ACGGGAAATTGTGAGGGACATGAAAGAAAAGCTAGCTTACATCGCTCTCGACTTTGAGCA
Textile-hemp-csa_locus_2263_iso_5_len_1488_ver_2      ACGGGAAATTGTGAGGGACATGAAGGAGAAGCTTGCCTACATAGCTCTTGACTTTGAGCA
Marijuana-scaffold122787                              ACGGGAAATTGTGAGGGACATGAAGGAGAAGCTTGCCTACATAGCTCTTGACTTTGAGCA
H.lupulus-HL.SW.v1.0.G017631.1                        ACGGGAAATTGTGAGGGACATGAAGGAGAAACTTGCTTACATTGCTCTTGACTTTGAGCA
                                                      ************************.**.**.**:** ***** ***** ***********

L.usitatissimum-Lus10001694                           GGAAATGGAAACAGCCAAAACTAGCTCATCTGTCGAGAAGAGTTACGAATTGCCTGATGG
Textile-hemp-csa_locus_2263_iso_5_len_1488_ver_2      GGAGATGGAGACAGCCAAGACTAGTTCTGCTGTTGAGAAGAGTTATGAGTTGCCAGACGG
Marijuana-scaffold122787                              GGAGATGGAGACAGCCAAGACTAGTTCTGCTGTTGAGAAGAGTTATGAGTTGCCAGACGG
H.lupulus-HL.SW.v1.0.G017631.1                        GGAGATGGAGACGGCCAAGACTAGTTCTGCTGTTGAGAAGAGTTATGAATTGCCAGATGG
                                                      ***.*****.**.*****.***** **: **** *********** **.*****:** **

L.usitatissimum-Lus10001694                           ACAGGTCATCACCATAGGTGCTGAGCGTTTCCGTTGCCCGGAAGTCCTGTTCCAACCATC
Textile-hemp-csa_locus_2263_iso_5_len_1488_ver_2      GCAAGTCATCACCATTGGCGCCGAGCGATTCCGATGTCCTGAAGTTCTTTTCCAACCTTC
Marijuana-scaffold122787                              GCAAGTCATCACCATTGGTGCCGAACGATTCCGATGTCCTGAAGTTCTTTTCCAACCTTC
H.lupulus-HL.SW.v1.0.G017631.1                        GCAAGTCATCACCATTGGTGCTGAGCGATTCCGATGCCCTGAAGTTCTTTTCCAACCCTC
                                                      .**.***********:** ** **.**:*****:** ** ***** ** ******** **

L.usitatissimum-Lus10001694                           CATGATAGGAATGGAAGCTGCAGGGATCCACGAGACAACTTACAATTCCATCATGAAGTG
Textile-hemp-csa_locus_2263_iso_5_len_1488_ver_2      ATTGGTTGGAATGGAAGCAGCAGGCATTCACGAAACTACTTACAACTCCATCATGAAGTG
Marijuana-scaffold122787                              ATTGGTTGGAATGGAAGCAGCAGGCATTCACGAAACTACTTACAACTCCATCATGAAGTG
H.lupulus-HL.SW.v1.0.G017631.1                        ATTGATTGGAATGGAAGCAGCAGGCATTCACGAAACTACTTACAACTCAATCATGAAGTG
                                                      .:**.*:***********:***** ** *****.**:******** **.***********

L.usitatissimum-Lus10001694                           CGACGTGGATATTAGGAAGGACTTGTACGGTAACATTGTGCTCAGTGGTGGTTCAACTAT
Textile-hemp-csa_locus_2263_iso_5_len_1488_ver_2      CGATGTTGATATCAGAAAGGATCTTTACGGTAACATTGTCCTGTCTGGTGGTTCAACCAT
Marijuana-scaffold122787                              CGATGTTGATATCAGAAAGGATCTTTACGGTAACATTGTCCTGTCTGGTGGTTCGACCAT
H.lupulus-HL.SW.v1.0.G017631.1                        CGATGTTGATATCAGAAAGGATCTTTACGGTAACATTGTCCTGTCTGGTGGTTCGACCAT
                                                      *** ** ***** **.*****  * ************** ** : *********.** **

L.usitatissimum-Lus10001694                           GTTCCCTGGTATTGCTGATAGAATGAGCAAAGAGATCTCTGCCCTTGCTCCAAGCAGCAT
Textile-hemp-csa_locus_2263_iso_5_len_1488_ver_2      GTTCCCAGGCATTGCTGATAGGATGAGCAAGGAAATCTCTGCTCTTGCACCAAGCTCTAT
Marijuana-scaffold122787                              GTTCCCAGGCATTGCTGATAGGATGAGCAAGGAAATCTCTGCTCTTGCACCAAGCTCTAT
H.lupulus-HL.SW.v1.0.G017631.1                        GTTCCCAGGCATTGCTGATAGAATGAGCAAGGAAATCTCTGCTCTTGCACCAAGCTCGAT
                                                      ******:** ***********.********.**.******** *****:******:  **

L.usitatissimum-Lus10001694                           GAAGATCAAGGTTGTTGCACCACCAGAAAGGAAATACAGTGTTTGGATTGGCGGTTCCAT
Textile-hemp-csa_locus_2263_iso_5_len_1488_ver_2      GAAGATTAAGGTGGTCGCACCACCAGAGAGGAAATACAGTGTTTGGATTGGTGGCTCTAT
Marijuana-scaffold122787                              GAAGATTAAGGTGGTCGCACCACCAGAGAGGAAATACAGTGTCTGGATTGGAGGCTCCAT
H.lupulus-HL.SW.v1.0.G017631.1                        GAAGATCAAGGTTGTCGCACCACCCGAGAGGAAATACAGTGTCTGGATTGGAGGCTCCAT
                                                      ****** ***** ** ********.**.************** ******** ** ** **

L.usitatissimum-Lus10001694                           CTTAGCTTCGCTTAGCACCTTCCAGCAG--------------------------------
Textile-hemp-csa_locus_2263_iso_5_len_1488_ver_2      TTTGGCTTCACTCAGCACCTTCCAGCAG--------------------------------
Marijuana-scaffold122787                              CTTGGCCTCTCTCAGCACCTTCCAGCAGGTTTATATTTTTTTCATATCTAAGTTTGGCAT
H.lupulus-HL.SW.v1.0.G017631.1                        TTTGGCCTCTCTCAGCACCTTCCAGCAG--------------------------------
                                                       **.** ** ** ***************

L.usitatissimum-Lus10001694                           ------------------------------------------------------------
Textile-hemp-csa_locus_2263_iso_5_len_1488_ver_2      ------------------------------------------------------------
Marijuana-scaffold122787                              GATGATCTTAATATGATCATTGAATAATTACTGATATCTTATTTTATCTTTTCCTTATGC
H.lupulus-HL.SW.v1.0.G017631.1                        ------------------------------------------------------------


L.usitatissimum-Lus10001694                           --ATGTGGATCGCAAAGGCAGAGTACGATGAGTCTGGACCGTCAATTGTTCACAGGAAGT
Textile-hemp-csa_locus_2263_iso_5_len_1488_ver_2      --ATGTGGATTGCAAAAGCAGAGTACGATGAGTCTGGGCCATCAATTGTTCATAGGAAAT
Marijuana-scaffold122787                              AGATGTGGATTGCAAAAGCAGAGTACGACGAGTCTGGGCCATCAATTGTTCATAGGAAAT
H.lupulus-HL.SW.v1.0.G017631.1                        ------------------------------------------------------------


L.usitatissimum-Lus10001694                           GCTTCTGA----------------------------------------------------
Textile-hemp-csa_locus_2263_iso_5_len_1488_ver_2      GCTTCTAAGCATGTGAGAATTAGAACTACTGCAGAGAGAAAAGCAAGTACTGTTGTTTTG
Marijuana-scaffold122787                              GCTTCTAAGCATGTGAGAATTAGAACTACTGCAGAGAGAAAAGCAAGTACTGTTGTTTTT
H.lupulus-HL.SW.v1.0.G017631.1                        ------------------------------------------------------------


L.usitatissimum-Lus10001694                           ------------------------------------------------------------
Textile-hemp-csa_locus_2263_iso_5_len_1488_ver_2      ATTATGTTGGAGAAAAGTGTTGTTTGCAGAAGGTTCATTCTAGCTCGTTACCTTTTTTTT
Marijuana-scaffold122787                              ATTATCTTGGAGAAAAGTGTTGTTTGCAGAAGGTTCATTCTAGCTCGTTACCTTTTTTTT
H.lupulus-HL.SW.v1.0.G017631.1                        ------------------------------------------------------------


L.usitatissimum-Lus10001694                           ------------------------------------------------------------
Textile-hemp-csa_locus_2263_iso_5_len_1488_ver_2      ATTCTCAACTTGAAGTGACTTCGGAAGGCCAGGAGTTAATTTATCCAATTTGTTTCTTTT
Marijuana-scaffold122787                              ATTCTCAACTTGAAGTGACTTCGGAAGGCCAGGAGTTAATTTATCCAATTTGTTTCTTTT
H.lupulus-HL.SW.v1.0.G017631.1                        ------------------------------------------------------------


L.usitatissimum-Lus10001694                           ------------------------------------------------------------
Textile-hemp-csa_locus_2263_iso_5_len_1488_ver_2      AAATTTTATTTTATTTTTGTTATTACGACTTCTTTTATTATCATTATTATTATTTTTTGG
Marijuana-scaffold122787                              AAATTTTATTTTATTTTTGTTATTACAACTTCTTTGATTATCATTATTATTATTTTTTGG
H.lupulus-HL.SW.v1.0.G017631.1                        ------------------------------------------------------------


L.usitatissimum-Lus10001694                           -------------
Textile-hemp-csa_locus_2263_iso_5_len_1488_ver_2      TCAAAAAAAAAAA
Marijuana-scaffold122787                              TCA----------
```

```
H.lupulus-HL.SW.v1.0.G017631.1                      -------------
```

Cyclo

```
L.usitatissimum-Lus10042553                     ------------------------------------------------------------
Marijuana-scaffold3694                          AAAAAAGTTTTTAGCTTAAATAAACTTTGAAAGGAATATGAGTTCTCAATCTAATTAACT
Textile-hemp-csa_locus_6842_iso_2_len_854_ver_2 ------------------------------------------------------------
H.lupulus-HL.SW.v1.0.G028342.1                  ------------------------------------------------------------

L.usitatissimum-Lus10042553                     ------------------------------------------------------------
Marijuana-scaffold3694                          GACTTTCTCTTTCAAATGATGTAGCTTTTATTATTATTATTAAGGATCTCTGAGTTGTTT
Textile-hemp-csa_locus_6842_iso_2_len_854_ver_2 ----------------------------------------TAAGGATCTCTGAGTTGTTT
H.lupulus-HL.SW.v1.0.G028342.1                  ----------------------------------------TATGGATCAATGAGTTGTTT

L.usitatissimum-Lus10042553                     ------------------------------------------------------------
Marijuana-scaffold3694                          TGTGAGGATTAT-ATGGATTATCTTTTAGTTGACATTCTTTGATTTTGAACAGGAACTTT
Textile-hemp-csa_locus_6842_iso_2_len_854_ver_2 TGTGAGGATTAT-ATGGATTATCTTTTAGTTGACATTCTTTGATTTTGAACAGGAACTTT
H.lupulus-HL.SW.v1.0.G028342.1                  TCTGGGGATTATATTGGATTAGCTTTTAGTTAACATTCTTTGGTTTTGAACAGGAACTTG

L.usitatissimum-Lus10042553                     ----------------------------------------------ATGGCGAAGAATCC
Marijuana-scaffold3694                          TTCTTCTGAAAACCA---------AAATATCACAAATTGGTTTTACAACATGTCGAACCC
Textile-hemp-csa_locus_6842_iso_2_len_854_ver_2 TTCTTCTGAAAACC---------AAAATATCACAAATTGGTTTTACAACATGTCGAACCC
H.lupulus-HL.SW.v1.0.G028342.1                  TTCTTTTGGAAGTTAAGTTAATAAAAAACTTTCAAATTGGATTGACAAAATGTCGAACCC
                                                                                              *: . *:.*** **

L.usitatissimum-Lus10042553                     AAAGGTGTTCTTTGACATTGTGATAGGCAAGGTGAAAACTGGCAGGGTGGTGATGGAGCT
Marijuana-scaffold3694                          CAAGGTCTTCTTTGACATCCTTATTGGAAAGATGAAGGCTGGCCGAGTTGTAATGGAATT
Textile-hemp-csa_locus_6842_iso_2_len_854_ver_2 CAAGGTCTTCTTTGACATCCTTATTGGAAAGATGAAGGCTGGCCGAGTTGTAATGGAATT
H.lupulus-HL.SW.v1.0.G028342.1                  CAAGGTCTTCTTTGACATCCTTATTGGAAAAATGAAGGCTGGCCGAGTTGTAATGGAGTT
                                                .***** ***********  * **:**.**..****..*****.*.** **.*****. *

L.usitatissimum-Lus10042553                     GTTTGCAGATACAGTCCCAAAAACAGCTGAGAATTTCCGAGCTCTATGCACGGGAGAGAA
Marijuana-scaffold3694                          GTTTGCCGATGTTACGCCAAAAACCGCTGAAAACTTCCGAGCTCTATGCACCGGAGAAAA
Textile-hemp-csa_locus_6842_iso_2_len_854_ver_2 GTTTGCCGATGTTACGCCAAAAACCGCTGAAAACTTCCGAGCTCTATGCACCGGAGAAAA
H.lupulus-HL.SW.v1.0.G028342.1                  GTTTGCTGATGCTACGCCGAAAACCGCAGAAAACTTCCGAGCCCTCTGCACTGGAGAGAA
                                                ****** ***. :.  **.*****.**:**.** ******** **.***** *****.**

L.usitatissimum-Lus10042553                     AGGGATTGGGAAAGCAGGGAAGCCTTTACATTACAAGGGCTCGGCTTTCCACCGTATAAT
Marijuana-scaffold3694                          AGGTCTTGGGGTATCAGGGAAGGCACTGCATTACAAAGGCTCAGCCTTTCACAGGATCAT
Textile-hemp-csa_locus_6842_iso_2_len_854_ver_2 AGGTCTTGGGGTATCAGGGAAGGCACTGCATTACAAAGGCTCAGCCTTTCACAGGATCAT
H.lupulus-HL.SW.v1.0.G028342.1                  AGGTCTTGGGGTATCAGGGAAGGCACTGCATTACAAAGGCTCAACCTTTCACCGGATCAT
                                                *** .*****.:* ******** *: *.********.*****..* ** ***.* **.**

L.usitatissimum-Lus10042553                     CCCGAATTTCATGTGTCAAGGTGGAGACTTTACCAGAGGAAATGGAACTGGTGGCGAATC
Marijuana-scaffold3694                          ACCAAACTTCATGTGTCAAGGTGGGGACTTTACCAGAGGTAATGGACCGGTGGAGAGTC
Textile-hemp-csa_locus_6842_iso_2_len_854_ver_2 ACCAAACTTCATGTGTCAAGGTGGGGACTTTACCAGAGGTAATGGACCGGTGGAGAGTC
H.lupulus-HL.SW.v1.0.G028342.1                  CCCAAATTTCATGTGTCAAGGTGGAGACTTCACCAGAGGTAATGGAACTGGAGGAGAGTC
                                                .**.** *****************.***** ********:*****.** **:**.**.**

L.usitatissimum-Lus10042553                     AATCTATGGGGAGAAGTTTGCAGATGAGAACTTCAAGCTGAAGCACACGGGTCCTGGGAT
Marijuana-scaffold3694                          AATTTATGGTATGAAGTTTGAGGATGAGAACTTCAAGTTGAAGCACACTGGCCCTGGAAT
Textile-hemp-csa_locus_6842_iso_2_len_854_ver_2 AATTTATGGTATGAAGTTTGAGGATGAGAACTTCAAGTTGAAGCACACTGGCCCTGGAAT
H.lupulus-HL.SW.v1.0.G028342.1                  TATTTATGGTATGAAGTTTGAGGATGAGAACTTCAAGCTGAAGCACACTGGCCCTGGTAT
                                                :** ***** .:********..*************** ********** ** ***** **

L.usitatissimum-Lus10042553                     CCTGTCCATGGCTAATGCGGGAAGGAACACTAACGGATCTCAGTTCTTTATTTGCACAGA
Marijuana-scaffold3694                          TCTTTCAATGGCTAATTCGGGAAGAAACACCAATGGCTCTCAGTTCTTTATATGCACTGA
Textile-hemp-csa_locus_6842_iso_2_len_854_ver_2 TCTTTCAATGGCTAATTCGGGAAGAAACACCAATGGCTCTCAGTTCTTTATATGCACCGA
H.lupulus-HL.SW.v1.0.G028342.1                  TCTTTCAATGGCGAATTCTGGAAGAAACACCAATGGCTCTCAGTTCTTTATTTGCACCGA
                                                 ** **.***** *** * *****.***** ** **.**************:***** **

L.usitatissimum-Lus10042553                     TAAGACAGCTTGGCTAGATGGGAAGCACGTTGTGTTTGGGAAAGTTGTTGATGGGTATGG
Marijuana-scaffold3694                          CAAGACCGACTGGCTTGATGGGAAACATGTTGTTTTTGGGAAAGTTGTTGATGGATATGG
Textile-hemp-csa_locus_6842_iso_2_len_854_ver_2 CAAGACCGACTGGCTTGATGGGAAACATGTTGTTTTTGGGAAAGTTGTTGATGGATATGG
H.lupulus-HL.SW.v1.0.G028342.1                  CAAGACCGAGTGGCTTGATGGGAAGCATGTAGTGTTTGGGAAAATTGTTGATGGATATGG
                                                 *****.*. *****:********.** **:** *********.**********.*****

L.usitatissimum-Lus10042553                     ATTTGTGAAGGAGATGGAGAAGGTTGGTTCAGATAGCGGAATTACTTCTCAGTCTGTTGT
Marijuana-scaffold3694                          CATAGTCAAAGAAATGGAGAAGGTTGGTTCAGGTAGTGGAATGACTTCTCAACCTGTTAT
Textile-hemp-csa_locus_6842_iso_2_len_854_ver_2 CATAGTCAAAGAAATGGAGAAGGTTGGTTCAGGTAGTGGAATGACTTCTCAACCTGTTAT
H.lupulus-HL.SW.v1.0.G028342.1                  TATAGTCAAAGAAATGGAGAAGGTTGGTTCAGGTAGTGGAATGACTTCTCAACCTGTTAT
                                                 :*:** **.**.*******************.*** ***** ********. *****.*

L.usitatissimum-Lus10042553                     TATTGAGGACTGTGGCCAGATTGATGATGAGCAACAGTGA--------------------
Marijuana-scaffold3694                          CATTGAAGATTGTGGACAGTTGACTGAGAACTAATCTTGTAGAAATTGTTAAATTAAGAT
Textile-hemp-csa_locus_6842_iso_2_len_854_ver_2 CATTGAAGATTGTGGACAGTTGACTGAGAACTAATCTTGTAGAAATTGTTAAATTAAGAT
H.lupulus-HL.SW.v1.0.G028342.1                  CATTGAAGATTGTGGACAGTTGACTGAGAACTAATAGTGTAGAAATGGTGAAATGAAGAT
                                                 *****.** *****.***:* ..*** .*  ** . **:

L.usitatissimum-Lus10042553                     ------------------------------------------------------------
Marijuana-scaffold3694                          TCCAAGCAACTTGTATTGTTTTTTGTTGCTTTTGCTTGAGGCCTCTCGGGGAAATAATTT
Textile-hemp-csa_locus_6842_iso_2_len_854_ver_2 TCCAAGCAACTTGTATTGTTTTTTGTTGCTTTTGCTTGAGGCCTCTCGGGGAAATAATTT
H.lupulus-HL.SW.v1.0.G028342.1                  TCCAAGCAAACTTT------TTTTTTGCCTTTTGCTTGAGGCCTTTCAAGGAAATAAGAA

L.usitatissimum-Lus10042553                     ------------------------------------------------------------
Marijuana-scaffold3694                          ATGTACTAAATACTTATGCAAAGCCTGAATTACAGGCTGGGG-------TACTCCAATCT
Textile-hemp-csa_locus_6842_iso_2_len_854_ver_2 ATGTACTAAATACTAATGCAAAGCCTGAATTACAGGCTGGG--------TACTCCAATCT
H.lupulus-HL.SW.v1.0.G028342.1                  ATGTTCA-------AATGCAAAGGGTGAATCCCATGCTGTATTTAGTTTAACTCAATTTT

L.usitatissimum-Lus10042553                     ------------------------------------------------------------
Marijuana-scaffold3694                          CTTAATATTTGACTCTGTATATTTTATGCTTTGATGATACTAAAATAAAATTTCTTTATT
Textile-hemp-csa_locus_6842_iso_2_len_854_ver_2 CTTAATATTTGACTCTGTATATTTTATGCTTTGATGATACTAAAATAAAATTTCTTTATT
H.lupulus-HL.SW.v1.0.G028342.1                  CATAATATTTGACTCTGTATATTTTGTGGTTTGA---TACTTTAATATAATTTCT-TATT

L.usitatissimum-Lus10042553                     ------------------------------------------------------------
Marijuana-scaffold3694                          CATGAAATGTGGGTTCTTTTTCCTGGAAAATATATTAATACCATTTATCATCCTGGAAAT
Textile-hemp-csa_locus_6842_iso_2_len_854_ver_2 CATGAAAAAAAA------------------------------------------------
H.lupulus-HL.SW.v1.0.G028342.1                  CATGAAA-----------------------------------------------------
```

```
L.usitatissimum-Lus10042553                    -----------------------------------------------
Marijuana-scaffold3694                         GACTTTTTTTTTAACATAACAATACTGCGAGCATTTGATGATTCAGA
Textile-hemp-csa_locus_6842_iso_2_len_854_ver_2 -----------------------------------------------
H.lupulus-HL.SW.v1.0.G028342.1                 -----------------------------------------------
```

GAPDH

```
L.usitatissimum-Lus10000872                        ------------------------------------------------------------
Marijuana-scaffold25527                            AAAAAAAAGAAAAAGAAAATTTGCATTTCTGACTTGTGCCGGTTTCGAAGAGGCACATAA
Textile-hemp-csa_locus_1460_iso_2_len_1872_ver_2   AAAAAAAAGAAAAAGAAAATTTGCATTTCTGACTTGTGCCGGTTTCGAAGAGGCACATAA
H.lupulus-HL.SW.v1.0.G008610.1                     ------------------------------------------------------------

L.usitatissimum-Lus10000872                        ------------------------------------------------------------
Marijuana-scaffold25527                            AGGCAGGAAGTTTTTTTTAGGGTTGTTGAGAAAGAAGA---AGAAGAGAAAAGAGAAAGC
Textile-hemp-csa_locus_1460_iso_2_len_1872_ver_2   AGGCAGGAAGTTTTTTTTAGGGTTGTTGAGAAAGAAGAAGAAGAAGAGAAAAGAGAAAGC
H.lupulus-HL.SW.v1.0.G008610.1                     ------------------------------------------------------------

L.usitatissimum-Lus10000872                        -------------------ATGGCCTTCTCCAGCCTCCTCAGATCTACCGCCGCCGCTGC
Marijuana-scaffold25527                            TCCGTACACACAGTCAACAATGGCGTTCTCTTCTCTGCTCAGATCTACCACCGGAGCTGT
Textile-hemp-csa_locus_1460_iso_2_len_1872_ver_2   TCCGTACACACAGTCAACAATGGCGTTCTCTTCTCTGCTCAGATCTACCACCGGAGCTGT
H.lupulus-HL.SW.v1.0.G008610.1                     ------------------------------------------------------------

L.usitatissimum-Lus10000872                        TCCCCTCGCCGACGCAGCTTCTCGATCCGCG------------CTCACTCCTTCCCTCCC
Marijuana-scaffold25527                            TCTGATTG------AAGCTTCTAGACCTGAAATCTTTGCGCCTCTTGCTTCTTCCAATCC
Textile-hemp-csa_locus_1460_iso_2_len_1872_ver_2   TCTGATTG------AAGCTTCTAGACCTGAAATCTTTGCGCCTCTTGCTTCTTCCAATCC
H.lupulus-HL.SW.v1.0.G008610.1                     ------------------------------------------------------------

L.usitatissimum-Lus10000872                        TTGCGATCGCTTCAAGGTTTCGAGCGTTGGCTTCTCTTCAAAGGCCGTCCCTATCTCAAC
Marijuana-scaffold25527                            T---------TCTAAGGCATCGAGCATTTGTTTCAATC------GCAACCCAAACTCTGC
Textile-hemp-csa_locus_1460_iso_2_len_1872_ver_2   T---------TCTAAGGCATCGAGCATTTGTTTCAATC------GCAACCCAAACTCTGC
H.lupulus-HL.SW.v1.0.G008610.1                     ------------------------------------------------------------

L.usitatissimum-Lus10000872                        AT---------------------------------------CGTCTTATTCCTTCCAGAC
Marijuana-scaffold25527                            AAGATTTCAATCAAGTTTATTTGGCACTGCTGTCCCAAGTGGATCATCTGTTTTACAGGT
Textile-hemp-csa_locus_1460_iso_2_len_1872_ver_2   AAGATTTCAATCAAGTTTATTTGGCACTGCTGTCCCAAGTGGATCATCTGTTTTACAGGT
H.lupulus-HL.SW.v1.0.G008610.1                     ------------------------------------------------------------

L.usitatissimum-Lus10000872                        ACGCAGTGGGAGAAGTGTCCAGCCCATCAAGGCCACCGCTACTGAAGTTCCTCCAACTGT
Marijuana-scaffold25527                            ATGCAATGTCAAGAGTGTCCAACCCATTAAAGCAACGGCCACTGAAGTACCACCAACTGT
Textile-hemp-csa_locus_1460_iso_2_len_1872_ver_2   ATGCAATGTCAAGAGTGTCCAACCCATTAAAGCAACGGCCACTGAAGTACCACCAACTGT
H.lupulus-HL.SW.v1.0.G008610.1                     ------------------------------------------------------------

L.usitatissimum-Lus10000872                        TCTGAGATCGAGGACTGCTGGGAAGACTAAGGTTGGGATCAACGGGTTTGGTCGTATTGG
Marijuana-scaffold25527                            TCAGAAATTGAAGACCAGTGGGAAgACAAAGGTTGGGATCAATGGTTTTGGTCGGATTGG
Textile-hemp-csa_locus_1460_iso_2_len_1872_ver_2   TCAGAAATTGAAGACCAGTGGGAACACAAAGGTTGGGATCAATGGTTTTGGTCGGATTGG
H.lupulus-HL.SW.v1.0.G008610.1                     ------------------------------------------------------------

L.usitatissimum-Lus10000872                        AAGATTGGTGCTCCGTATTGCTACATCTAGGGATGATATCGATGTGGTGGCAGTCAATGA
Marijuana-scaffold25527                            AAGATTGGTTTTACGAGTAGCAACATTCAGGGATGATATTGACGTAGTGGCAGTGAATGA
Textile-hemp-csa_locus_1460_iso_2_len_1872_ver_2   AAGATTGGTTTTACGAGTAGCAACATTCAGGGATGATATTGACGTAGTGGCAGTGAATGA
H.lupulus-HL.SW.v1.0.G008610.1                     ------------------------------------------------------------

L.usitatissimum-Lus10000872                        TCCCTTTATTGATGCTAAATACATGGCTTACATGTTGAAGTATGATTCTACTCATGGGAA
Marijuana-scaffold25527                            TCCTTTCATTGATGCTAAATACATGGCCTACATGTTCAAATATGATTCTACTCATGGATT
Textile-hemp-csa_locus_1460_iso_2_len_1872_ver_2   TCCTTTCATTGATGCTAAATACATGGCCTACATGTTCAAATATGATTCTACTCATGGATT
H.lupulus-HL.SW.v1.0.G008610.1                     ------------------------------------------------------------

L.usitatissimum-Lus10000872                        TTTCAACGGGACCATCACTGTTTTGGATGAGTCTACATTGGAAATCAACGGCAAACAAAT
Marijuana-scaffold25527                            ATTCAAAGGAAGCATTAGGGTTGTAGATGATTCAACCTTAGAAATCAATGGGAAGCAGAT
Textile-hemp-csa_locus_1460_iso_2_len_1872_ver_2   ATTCAAAGGAAGCATTAGGGTTGTAGATGATTCAACCTTAGAAATCAATGGGAAGCAGAT
H.lupulus-HL.SW.v1.0.G008610.1                     ------------------------------------------------------------

L.usitatissimum-Lus10000872                        CAAAATTTCCAGCAAGAGGGACCCTGCAGATATTCCATGGGGTGATTATGGAGCAGAATA
Marijuana-scaffold25527                            AAAGGTTCTGAGCAAAAGGGACCCTGCTGAGATTCCTTGGGGTGATTATGGAGCTGAGTA
Textile-hemp-csa_locus_1460_iso_2_len_1872_ver_2   AAAGGTTCTGAGCAAAAGGGACCCTGCTGAGATTCCTTGGGGTGATTATGGAGCTGAGTA
H.lupulus-HL.SW.v1.0.G008610.1                     ------------------------------------------------------------

L.usitatissimum-Lus10000872                        TGTTGTTGAATCTTCTGGAGTCTTCACAACTATCGACAAGGCTGCAGCTCACAAAAAGGG
Marijuana-scaffold25527                            TGTCGTTGAATCTTCCGGTGTTTTCACAACAATTGACAAGGCTTCAGCACATAAAAAGGG
Textile-hemp-csa_locus_1460_iso_2_len_1872_ver_2   TGTCGTTGAATCTTCCGGTGTTTTCACAACAATTGACAAGGCTTCAGCACATAAAAAGGG
H.lupulus-HL.SW.v1.0.G008610.1                     --------------------------------------------------------AGGG
                                                                                                           ****

L.usitatissimum-Lus10000872                        AGGTGCTACAAAGGTGGTCATATCAGCCCCCTCTGCTGATGCACCAATGTTTGTTGTTGG
Marijuana-scaffold25527                            TGGAGCCAAGAAAGTAGTTATATCAGCTCCATCAGCTGATGCACCTATGTTCGTGGTTGG
Textile-hemp-csa_locus_1460_iso_2_len_1872_ver_2   TGGAGCCAAGAAAGTAGTTATATCAGCTCCATCAGCTGATGCACCTATGTTCGTGGTTGG
H.lupulus-HL.SW.v1.0.G008610.1                     TGGAGCCAAGAAAGTAGTTTTATCAGCTCCATCAGCTGATGCTCCCATGTTTGTGGTTGG
                                                   :**:** *..**.**.** :******* **.**:********:** ***** ** *****

L.usitatissimum-Lus10000872                        AGTTAATGAGCAGACATACCAGCCAAACATGGATGTTGTCTCTAATGCAAGCTGCACGAC
Marijuana-scaffold25527                            AGTAAATGAGCAGACATACAAGCCAAACATGGACATTGTTTCCAATGCAAGCTGTACTAC
Textile-hemp-csa_locus_1460_iso_2_len_1872_ver_2   AGTAAATGAGCAGACATACAAGCCAAACATGGACATTGTTTCCAATGCAAGCTGTACTAC
H.lupulus-HL.SW.v1.0.G008610.1                     AGTAAATGAGCAGACATACAAGCCAAACATGGACATTGTTTCAAATGCAAGCTGTACTAC
                                                   ***:***************.************* .**** ** ********** ** **

L.usitatissimum-Lus10000872                        AAATTGTCTTGCTCCCCTTGCCAAGGTGGTTCATGAAGAATTTGGCATTCTTGAAGGGTT
Marijuana-scaffold25527                            TAACTGTCTTGCTCCTCTAGCTAAGGTGGTTCATGAGGAATTTGGtATTCTTGAAGGTTT
Textile-hemp-csa_locus_1460_iso_2_len_1872_ver_2   TAACTGTCTTGCTCCTCTAGCTAAGGTGGTTCATGAGGAATTTGGCATTCTTGAAGGTTT
H.lupulus-HL.SW.v1.0.G008610.1                     CAACTGTCTTGCTCCTCTTGCTAAGGTCGGTT----------------------------
                                                    ** *********** **:** ***** * *

L.usitatissimum-Lus10000872                        GATGACAACAGTTCATGCAACTACAGCTACCCAAAAGACTGTCGATGGCCCATCAATGAA
Marijuana-scaffold25527                            AATGACAACGGTTCATGCAACTACAGCAACACAGAAGACCGTTGATGGCCCATCAATGAA
Textile-hemp-csa_locus_1460_iso_2_len_1872_ver_2   AATGACAACGGTTCATGCAACTACAGCAACACAGAAGACCGTTGATGGCCCATCAATGAA
H.lupulus-HL.SW.v1.0.G008610.1                     ------------------------------------------------------------
```

```
L.usitatissimum-Lus10000872                          AGATTGGCGTGGTGGTCGTGGAGCTAGTCAGAATATCATTCCAAGTTCTACCGGTGCAGC
Marijuana-scaffold25527                              GGATTGGAGAGGAGGCCGTGGAGCTGGACAAAATATtATTCCTAGTTCTACTGGTGCAGC
Textile-hemp-csa_locus_1460_iso_2_len_1872_ver_2     GGATTGGAGAGGAGGCCGTGGAGCTGGACAAAATATCATTCCTAGTTCTACTGGTGCAGC
H.lupulus-HL.SW.v1.0.G008610.1                       ------------------------------------------------------------

L.usitatissimum-Lus10000872                          GAAGGCTGTTGGAAAGGTACTTCCAGAGCTGAACGGAAAACTTACTGGCATGGCTTTCCG
Marijuana-scaffold25527                              AAAGGCTGTTGGTAAGGTTCTCCCAGAACTGAATGGAAAGCTTACTGGAATGGCCTTCCG
Textile-hemp-csa_locus_1460_iso_2_len_1872_ver_2     AAAGGCTGTTGGTAAGGTTCTCCCAGAACTGAATGGAAAGCTTACTGGAATGGCCTTCCG
H.lupulus-HL.SW.v1.0.G008610.1                       ------------------------------------------------------------

L.usitatissimum-Lus10000872                          TGTCCCAACGCCTAATGTTTCTGTTGTGGACTTAACTTGTCGACTTGAGAAGAGTGCATC
Marijuana-scaffold25527                              TGTTCCaACTCCTAATGTCTCAGTGGTgGACTTAACTTGTCGACTTGAGAAGAGTGCTTC
Textile-hemp-csa_locus_1460_iso_2_len_1872_ver_2     TGTTCCTACTCCTAATGTCTCAGTGGTAGACTTAACTTGTCGACTTGAGAAGAGTGCTTC
H.lupulus-HL.SW.v1.0.G008610.1                       ------------------------------------------------------------

L.usitatissimum-Lus10000872                          ATACGATGATGTTAAAGCTGCTATTAAGTATGCATCTGAGGGACCATTGAAGGGCATTCT
Marijuana-scaffold25527                              TTATGAA-----------------------------------------------------
Textile-hemp-csa_locus_1460_iso_2_len_1872_ver_2     TTATGAAGATGTCAAGGCAGCCATTAAGTATGCGTCAGAGGGACCACTTAAAGGCATTCT
H.lupulus-HL.SW.v1.0.G008610.1                       ------------------------------------------------------------

L.usitatissimum-Lus10000872                          TGGATACACTGATGAGGATGTCGTTTCTAATGATTTCATCGGCGACTCAAGGTCAAGCAT
Marijuana-scaffold25527                              ------------------------------------------------------------
Textile-hemp-csa_locus_1460_iso_2_len_1872_ver_2     TGGGTACACAGATGAAGATGTTGTCTCCAATGATTT-GTTGGTGACACAAGGTCAAGTAT
H.lupulus-HL.SW.v1.0.G008610.1                       ------------------------------------------------------------

L.usitatissimum-Lus10000872                          ATTCGATGCAAAGGCCGGGATAGGGTTGAGCAGTTCCTTCATGAAGCTCGTGTCGTGGTA
Marijuana-scaffold25527                              ------------------------------------------------------------
Textile-hemp-csa_locus_1460_iso_2_len_1872_ver_2     TTTCGATGCCAAGGCTGGAATAGGGCTTAGCACATCCTTCATGAAGCTTGTGTCATGGTA
H.lupulus-HL.SW.v1.0.G008610.1                       ------------------------------------------------------------

L.usitatissimum-Lus10000872                          CGACAATGAGTGGGGTTACAGCAACCGAGTTCTGGACCTCATCGAGCACATGGCGTTGGT
Marijuana-scaffold25527                              ------------------------------------------------------------
Textile-hemp-csa_locus_1460_iso_2_len_1872_ver_2     CGATAACGAATGGGGATACAGCAACCGTGTTTTGGACCTTATTGAGCACATGGCGTTGGT
H.lupulus-HL.SW.v1.0.G008610.1                       ------------------------------------------------------------

L.usitatissimum-Lus10000872                          AGCTTCCCT--GAAGTGA------------------------------------------
Marijuana-scaffold25527                              ------------------------------------------------------------
Textile-hemp-csa_locus_1460_iso_2_len_1872_ver_2     AGCGGCTACCTACTAAGTATTGTTGCTGCTGCAACAACAATTTTTTTAGAGCTCTTGCT
H.lupulus-HL.SW.v1.0.G008610.1                       ------------------------------------------------------------

L.usitatissimum-Lus10000872                          ------------------------------------------------------------
Marijuana-scaffold25527                              ------------------------------------------------------------
Textile-hemp-csa_locus_1460_iso_2_len_1872_ver_2     ATGAGTTTGGTTGGAGAGTAGTTAATACATTTTGATAGGGTTTCGGGTTTTTCCTCATCT
H.lupulus-HL.SW.v1.0.G008610.1                       ------------------------------------------------------------

L.usitatissimum-Lus10000872                          ------------------------------------------------------------
Marijuana-scaffold25527                              ------------------------------------------------------------
Textile-hemp-csa_locus_1460_iso_2_len_1872_ver_2     CATTTGGTGCGATTTAAAGATTTCGCATGGTTCAGTTTGAAAATAATGAAAATTCTGAGA
H.lupulus-HL.SW.v1.0.G008610.1                       ------------------------------------------------------------

L.usitatissimum-Lus10000872                          ------------------------------------------------------------
Marijuana-scaffold25527                              ------------------------------------------------------------
Textile-hemp-csa_locus_1460_iso_2_len_1872_ver_2     TGTATGGGCTTTGATAATCATTGTCTCATTGGAGTTGATGCCATTAATATACATATTGAG
H.lupulus-HL.SW.v1.0.G008610.1                       ------------------------------------------------------------

L.usitatissimum-Lus10000872                          ------------------------------------------------------------
Marijuana-scaffold25527                              ------------------------------------------------------------
Textile-hemp-csa_locus_1460_iso_2_len_1872_ver_2     AAGGGTTTCTTCTGCTTCTCTTGTATGGCTTTTCTTTCTAGTAACTTTTGACATGAATCT
H.lupulus-HL.SW.v1.0.G008610.1                       ------------------------------------------------------------

L.usitatissimum-Lus10000872                          ------------------------------------------------------------
Marijuana-scaffold25527                              ------------------------------------------------------------
Textile-hemp-csa_locus_1460_iso_2_len_1872_ver_2     TTCATCTCTTGATAAAAATGGTTTCATTTCCTATGTTGATCAATGCTAGTATCAACTACC
H.lupulus-HL.SW.v1.0.G008610.1                       ------------------------------------------------------------

L.usitatissimum-Lus10000872                          ------------------------------------------------------------
Marijuana-scaffold25527                              ------------------------------------------------------------
Textile-hemp-csa_locus_1460_iso_2_len_1872_ver_2     AAATACATCTGAATATGGGTACAGCTCAAGTTATAGGCATAATAAATAATTTTTAACCAC
H.lupulus-HL.SW.v1.0.G008610.1                       ------------------------------------------------------------

L.usitatissimum-Lus10000872                          ----------------------------------
Marijuana-scaffold25527                              ----------------------------------
Textile-hemp-csa_locus_1460_iso_2_len_1872_ver_2     ATTATAAATGCAAATGGCATCAAATTAATTGTCT
H.lupulus-HL.SW.v1.0.G008610.1                       ----------------------------------
```

eTIF4E

```
L.usitatissimum-Lus10023733                       -----------------------------------------------------------A
Marijuana-scaffold35425                           ---------------GAGAGATACACTTTCATTTCAAAG--CTCCAACGAATCCTAACCA
Textile-hemp-csa_locus_9035_iso_2_len_909_ver_2   CCGCTCTTCCGATCTGAGAGATACACTTTCATTTCAAAG--CTCCAACGAATCCTAACCA
H.lupulus-HL.SW.v1.0.G000377.1                    -------------------GATTCACATTCATCTCACTTCCATACATCGAATCGGAACCA
                                                                                                             *

L.usitatissimum-Lus10023733                       TGGCGACCAACGACACTGCGGCGACCGAAGTAAGCGACGCATCGGTGGTTACGTCCGCCG
Marijuana-scaffold35425                           TGGCGACTGAGATGGCAGCGGCCACCGAGGCCAACATTTTAGCAGAGCCCGAGGCTGCAG
Textile-hemp-csa_locus_9035_iso_2_len_909_ver_2   TGGCGACTGAGATAGCAGCGGCCACCGAGGCCAACATTTTAGCAGAGCCCGAGGCTGCAG
H.lupulus-HL.SW.v1.0.G000377.1                    TGGCGACTGACATAGCAGCAGGCACGGAGGCGAACAGTGTAGCAGAGCCCGAGATTGCAG
                                                  ******* .* .: .*:**.* **  **.*  *.*.    * *.*:*   ..*   **.*

L.usitatissimum-Lus10023733                       ACGCAATGGCG---GA------GACGTCCCCTCAGTCTCACAAGCTCCACAGAAAGTGGA
Marijuana-scaffold35425                           CGGCTATTGTGGCCGAGAACAAGACGCAAGGCGAGCCACACAAGTTGGATAAGAAATGGA
Textile-hemp-csa_locus_9035_iso_2_len_909_ver_2   CGGCTATTGTGGCCGAGAACAAGACGCAAGGCGAGCCACACAAGTTGGATAAGAAATGGA
H.lupulus-HL.SW.v1.0.G000377.1                    CGGCAATGGTGGCCGAAAGCAAGACACAGACCGAGCCGCACAAGTTGGATAAGAAGTGGA
                                                  . **:** * *   **      ***. .     ** * ****** *  * *..**.****

L.usitatissimum-Lus10023733                       CCTTCTGGTTCGACAACCAATCCAAACCCAAGCAGGGCGCCGCTTGGGGCACCTCTCTTC
Marijuana-scaffold35425                           CCTTTTGGTTCGATAACCAATCTAAGCCTAAGCAAGGCGCCGCCTGGGGCACTTCTCTTC
Textile-hemp-csa_locus_9035_iso_2_len_909_ver_2   CCTTTTGGTTCGATAACCAATCTAAGCCTAAGCAAGGCGCCGCCTGGGGCACTTCTCTTC
H.lupulus-HL.SW.v1.0.G000377.1                    CCTTTTGGTTCGATAACCAGTCCAAGCCCAAGCAAGGCGCTGCCTGGGGAACTTCTCTTC
                                                  **** ******** *****.** **.** *****.***** ** *****.** *******

L.usitatissimum-Lus10023733                       GTAGTGTCTACACCTTCGACACCGTCGAGGAATTCTGGTGTTTGTATGAACAGGTATTTA
Marijuana-scaffold35425                           GCTCTGTCTACACTTTTGACACCGTCGAAGAATTTTGGTGTTTGTATGACCAGGTATTCA
Textile-hemp-csa_locus_9035_iso_2_len_909_ver_2   GCTCTGTCTACACTTTTGACACCGTTGAAGAATTTTGGTGTTTGTATGACCAGGTATTCA
H.lupulus-HL.SW.v1.0.G000377.1                    GCTCTGTCTACACTTTTGACACCGTCGAAGAATTTTGGTG--------------------
                                                  * : ********* ** ******** **.***** *****

L.usitatissimum-Lus10023733                       AGCCTAGCAAGCTACCTGGACAAGCTGAATTCCACCTGTTTAGGGATGGGGTTGAACCCA
Marijuana-scaffold35425                           AGCCTAGCAAGTTGACATCACATGCAGATTTATAATTACTCTA-----------------
Textile-hemp-csa_locus_9035_iso_2_len_909_ver_2   AGCCTAGCAAGTTGACATCACATGCAGATTTCCATTTGTTCAAAGCTGGGGTTGAACCCA
H.lupulus-HL.SW.v1.0.G000377.1                    ------------------------------------------------------------

L.usitatissimum-Lus10023733                       AGTGGGAAGATCCAGAGTGTGCTAATGGGGGAAAGTGGAGTGTTACCTGTAGTAAAAACA
Marijuana-scaffold35425                           -------------------------------CAGGTTTACTGTTATCAGCAGTAGAAAAT
Textile-hemp-csa_locus_9035_iso_2_len_909_ver_2   AGTGGGAAGATCCTGAATGTGCTAATGGAGGAAAGTGGACTGTTATCAGCAGTAGAAAAT
H.lupulus-HL.SW.v1.0.G000377.1                    ------------------------------------------------------------

L.usitatissimum-Lus10023733                       CCACCTTGGATAAGATGTGGCTCGAAACTCTGATGGCTTTGATTGGAGAGCAATTTGATG
Marijuana-scaffold35425                           CTACCCTTGACACTATGTGGTTAGAAACTTTGATGGCTTTGATTGGAGAGCAATTTGATG
Textile-hemp-csa_locus_9035_iso_2_len_909_ver_2   CTACCCTTGACACTATGTGGTTAGAAACTTTGATGGCTTTGATTGGAGAGCAATTTGATG
H.lupulus-HL.SW.v1.0.G000377.1                    ------------------------------------------------------------

L.usitatissimum-Lus10023733                       AATCCGAGGAGATATGTGGCGTGGTTGCCAGTGTGCGTACAAGGCAAGATAAACTTGCTC
Marijuana-scaffold35425                           AGGCTGATGAGATTTGTGGTGTGGTCGCAAGCGTGCGCCAGAGGCAGGACAAACTTGCGC
Textile-hemp-csa_locus_9035_iso_2_len_909_ver_2   AGGCTGATGAGATTTGTGGTGTGGTCGCAAGCGTGCGCCAGAGGCAGGACAAACTTGCGC
H.lupulus-HL.SW.v1.0.G000377.1                    ------------------------------------------------------------

L.usitatissimum-Lus10023733                       TCTGGACTAAGACGGCTGCTAATGAAGCTGTCCAGATGGGCATCGGAAGAAAGTGGAAAG
Marijuana-scaffold35425                           TCTGGACCAAGACAGCTGCCAATGAGGCTGTTCAGATGAGCATTGGAAAGAAGTGGAAGG
Textile-hemp-csa_locus_9035_iso_2_len_909_ver_2   TCTGGACCAAGACAGCTGCCAATGAGGCTGTTCAGATGAGCATTGGAAAGAAGTGGAAGG
H.lupulus-HL.SW.v1.0.G000377.1                    ------------------------------------------------------------

L.usitatissimum-Lus10023733                       ATATCATCGATGTCACTGACAAACTCACCTACAATTTCCATGATGATTCTCGCAGGGAAA
Marijuana-scaffold35425                           AGATACTAGACACAACTGACAAAATCACTTACAGCTTCCATGATGATTCGAAAAGAGAGA
Textile-hemp-csa_locus_9035_iso_2_len_909_ver_2   AGATACTAGACACAACTGACAAAATCACTTACAGCTTCCATGATGATTCGAAGAGAGAGA
H.lupulus-HL.SW.v1.0.G000377.1                    ------------------------------------------------------------

L.usitatissimum-Lus10023733                       GATCTGTGAAGAGCCGATACAGCGTATGA-------------------------------
Marijuana-scaffold35425                           GATCTGCAAAGGGTCGTTACACTATACCATGAACATTACTCGACAATTTGAGGCTTGATC
Textile-hemp-csa_locus_9035_iso_2_len_909_ver_2   GATCTGCGAAGGGTCGTTACACTATACCATGAACATTACTCGACAATTTGAGGCTTGATC
H.lupulus-HL.SW.v1.0.G000377.1                    ------------------------------------------------------------

L.usitatissimum-Lus10023733                       ------------------------------------------------------------
Marijuana-scaffold35425                           CTTTACTTAGTTACTTGCAATTTTACAAAATACTAGCAGCGGCAACTCATTGCAGATTTT
Textile-hemp-csa_locus_9035_iso_2_len_909_ver_2   CTTTACTTAGTTACTTGCAATTTTACAAAATACTAGCAGCGGCAACTCATTGCAGATTTT
H.lupulus-HL.SW.v1.0.G000377.1                    ------------------------------------------------------------

L.usitatissimum-Lus10023733                       ------------------------------------------------------------
Marijuana-scaffold35425                           GATGTTTTCTGTCTATTGAGGAAAACCAATTAGATTAAATATTTTTTTGTGGTGTTTTTT
Textile-hemp-csa_locus_9035_iso_2_len_909_ver_2   GATGT-TTCTGTCTATTGAGGAAAACCAATTAGATCAAATAT-TTTTTGTGGTGTTTTTT
H.lupulus-HL.SW.v1.0.G000377.1                    ------------------------------------------------------------

L.usitatissimum-Lus10023733                       ------------------------------------------------------------
Marijuana-scaffold35425                           GATTGTAACATGACAACCGCTCTTTATTTTTTTTGTATTGCAAATTTTTCATCAAAAA--
Textile-hemp-csa_locus_9035_iso_2_len_909_ver_2   GATTGTAATATGACAACCGCTCTTTATTTT-TTTGTATTGCAAATTTTTTATCAAAAAAA
H.lupulus-HL.SW.v1.0.G000377.1                    ------------------------------------------------------------

L.usitatissimum-Lus10023733                       --------------
Marijuana-scaffold35425                           AATGATTACTTAAA
Textile-hemp-csa_locus_9035_iso_2_len_909_ver_2   AATGATTACTTAAA
H.lupulus-HL.SW.v1.0.G000377.1                    --------------
```

TIP41

```
L.usitatissimum-Lus10026859                         ------------------------------------------------------------
Textile-hemp-csa_locus_3380_iso_1_len_1164_ver_2    ATTATTTTGATTGCCCATTTATAATGAAGTTGATCAAAATTCAAAACTCAAAAGTTTGGC
H.lupulus-HL.Tea.v1.0.G021288.1                     ------------------------------------------------------------
Marijuana-scaffold76878                             ATTATTTTGATTGCCCATTTATAtTGAgGTTGATCAAAATTCAAAACTCAAAAGTTcGGC

L.usitatissimum-Lus10026859                         ------------------------------------------------------------
Textile-hemp-csa_locus_3380_iso_1_len_1164_ver_2    TTGGGCCCAAATTCCAACAGTGAATAATAAGGCAAGGCAA--AGGGACTCAAAAC
H.lupulus-HL.Tea.v1.0.G021288.1                     ------------------------------------------------------------
Marijuana-scaffold76878                             TTGGGCCCAAATTCCAACAGTGAATAATAAGGCAAGGCAAaggtacctttgaggcgcgAC

L.usitatissimum-Lus10026859                         -----------------------------ATGGCCGAGGAGGTAGACGACAAGGACCTGA
Textile-hemp-csa_locus_3380_iso_1_len_1164_ver_2    CGAAGAATCGATCGAACGCGACTGTGGAAATGGAAGTGGAGGTTGACCATAACGAGCTCA
H.lupulus-HL.Tea.v1.0.G021288.1                     ------------------------------------------------------------
Marijuana-scaffold76878                             CGAAGAATCGATCGAACGCGACTGTGGAAATGGAAGTGGAGGTTGACCATAACGAGCTCA

L.usitatissimum-Lus10026859                         AAGCCGCCGGAGCTGAGCTTTTGACCGACGGCCTCCGCGGACTCCGTATTCATGGCTGGG
Textile-hemp-csa_locus_3380_iso_1_len_1164_ver_2    AGGCGGCTGGAGCCGAGCTTCTAACTGAAGGACGTCATGGCATCCGTATCCATGGTTGGG
H.lupulus-HL.Tea.v1.0.G021288.1                     ------------------------------------------------------------
Marijuana-scaffold76878                             AGGCGGCTGGAGCCGAGCTTCTAACTGAAGGACGTCATGGCATCCGTATCCATGGTTGGG

L.usitatissimum-Lus10026859                         AGATCGTCTCCAAGGCTGGCTCTATTCTCAACTCCTCCGCTGTTGAACATGGACGTTTGC
Textile-hemp-csa_locus_3380_iso_1_len_1164_ver_2    AGGTCGAGTCTCGCAATTGCTCCATTCTCACCTCTTCCATCTTTGAAC------------
H.lupulus-HL.Tea.v1.0.G021288.1                     ------------------------------------------------------------
Marijuana-scaffold76878                             AGGTCGAGTCTCGCAATTGCTCCATTCTCACCTCTTCCAcCTTTGAAC------------

L.usitatissimum-Lus10026859                         TGCACGTACACCTCGGAACTCTTTGTTGTCACAAAGTGGTGGTGTTGGCCAAAGTTTGTG
Textile-hemp-csa_locus_3380_iso_1_len_1164_ver_2    ------------------------------------------------------------
H.lupulus-HL.Tea.v1.0.G021288.1                     ------------------------------------------------------------
Marijuana-scaffold76878                             ------------------------------------------------------------

L.usitatissimum-Lus10026859                         CCGGTTGTCCTGTGCCGTACTGGTGTAATATACATGCTCAATCTACTTGCAGGTGGGAGG
Textile-hemp-csa_locus_3380_iso_1_len_1164_ver_2    ---------------------------------------------------AGTGGGGAG
H.lupulus-HL.Tea.v1.0.G021288.1                     ------------------------------------------------------------
Marijuana-scaffold76878                             ---------------------------------------------------AGTGGGGAG

L.usitatissimum-Lus10026859                         AGAAACTGCAGACCTCTCACCTACCCGAGATGGTGTTTGGCGACAGCTGTCTGGTTCTCA
Textile-hemp-csa_locus_3380_iso_1_len_1164_ver_2    AAAAGCTTCAGACATCCCATTTGCCAGAAATGATATTTGGGGACAGTTGTTTGGTTCTTA
H.lupulus-HL.Tea.v1.0.G021288.1                     ------------------------------------------------------------
Marijuana-scaffold76878                             AAAAGCTTCAGACATCCCATTTGCCAGAAATGATATTTGGGGACAGTTGTTTGGTTCTTA

L.usitatissimum-Lus10026859                         AACATATCAAGAGTGGCACCAAAATCCATTTTAATGCATTTGATGCGCTAACCGGTTGGA
Textile-hemp-csa_locus_3380_iso_1_len_1164_ver_2    AGCACATTCCTAGTGGCACTAAAATTCATTTTAATGCGTTTGATGCTCTACTTGGATGGA
H.lupulus-HL.Tea.v1.0.G021288.1                     ------------------------------------------------------------
Marijuana-scaffold76878                             AGCACAaTCCTAGTGGCACTAAAATTCATTTTAATGCGTTTGATGCTCTACTgGGATGGA

L.usitatissimum-Lus10026859                         AGCAGGAAGCTTTACCCCCAGTTGAAGTTCCTGCTGCTGCACAATGGAAATTCAGAAGCA
Textile-hemp-csa_locus_3380_iso_1_len_1164_ver_2    AACAGGAAGCTCTGCCGCCTGTGGAAGTTCCGGCCGCTGAAAAATGGAAATTCCGCAGCA
H.lupulus-HL.Tea.v1.0.G021288.1                     ------------------------------------------------------------
Marijuana-scaffold76878                             AACAGGAAGCTCTGCCGCCTGTGGAAGTTCCGGCCGCTGAAAAATGGAAATTCCGCAGCA

L.usitatissimum-Lus10026859                         AGCCGTTCCAACAGGTGATACTAGATTATGATTATACATTCACGACACCATACATTGGAA
Textile-hemp-csa_locus_3380_iso_1_len_1164_ver_2    AACCATCCCAACAGGTCATTTTGGATTATGATTACACTTTTACAACACCTTATCGTGGAA
H.lupulus-HL.Tea.v1.0.G021288.1                     ------------------------------------------------------------
Marijuana-scaffold76878                             AACCATCCCAACAGGTCATTTTGGATTATGATTACACTTTTACAACACCTTATCGTGGAA

L.usitatissimum-Lus10026859                         GTGAAACTATTGAGA---------CCCAGTGTGAAAGTGGAGAACCCATTTCTGCAAGTG
Textile-hemp-csa_locus_3380_iso_1_len_1164_ver_2    GTGAAATGATTGAGATGGATGCAACCGAGCAAGAAAGAGAGGATTCCTCTGAGGGAACCT
H.lupulus-HL.Tea.v1.0.G021288.1                     ------------------------------------------------------------
Marijuana-scaffold76878                             GTGAAAcaATTGAGATtGATGCAACCGAGCAAGAAAGAGAGGATTCCTCTGAGGGAACCT

L.usitatissimum-Lus10026859                         GCAGGCTTCAATGGGAGGATTGCAAGGAACGAATTGATGTTGCTTCCCTAGCTTCAAAAG
Textile-hemp-csa_locus_3380_iso_1_len_1164_ver_2    ACAATGTCCAGTGGGAGGAGAGCAAGGAGAAAATTGATATGGCTGCACTGGCACCCAAAG
H.lupulus-HL.Tea.v1.0.G021288.1                     ------------------------------------------------------------
Marijuana-scaffold76878                             ACAATcTCCAGTGGGAGGAGAGCAAGGAGAAAATTGATATGGCTGCACTGGCACCCAAAG

L.usitatissimum-Lus10026859                         AGCCTATTCTTTTCTATGACGAGGTTGTTCTTTATGAAGATGAACTGGCTGATAATGGAG
Textile-hemp-csa_locus_3380_iso_1_len_1164_ver_2    AGCCTATTCTCTTCTATGATGAGGTAATATTATATGAAGATGAACTTGCAGATAATGGGG
H.lupulus-HL.Tea.v1.0.G021288.1                     ------------------------------------------------------------
Marijuana-scaffold76878                             AGCCTATTCTCTTCTATGATGAGGTAATATTgTATGAAGATGAACTTGCAGATAATGGGG

L.usitatissimum-Lus10026859                         TGTCACTTTTAACAGTAAAAGTGAGAGTGATGCCAAGTTATTGGTTTCTTCTTTTGCGAT
Textile-hemp-csa_locus_3380_iso_1_len_1164_ver_2    TGTCGCTTCTAACTGTTAAAGTGAGAGTCATGCCAAGTTCTTGGTTTCTTCTATTGCGCT
H.lupulus-HL.Tea.v1.0.G021288.1                     ------------------------------------------------------------
Marijuana-scaffold76878                             TGTCGCTTCTAACTGTTAAAGTGAGAGTCATGCCAAGTTCTTGGTTTCTTCTATTGCGCT

L.usitatissimum-Lus10026859                         TTTGGCTTAGAGTTGATGGAGTCCTGATGAGATTACGGGACACTCGCATCCATTGTGCTT
Textile-hemp-csa_locus_3380_iso_1_len_1164_ver_2    ATTGGCTCAGAGTTGATGGAGTGCTTATGAGACTAAGAGACACTCGAATGCATTGTGTAT
H.lupulus-HL.Tea.v1.0.G021288.1                     ----GCTCAGAGTTGATGGAGTGCTTATGAGAATAAGAGACACTCGTATGCATTGTGTAT
Marijuana-scaffold76878                             ATTGGCTCAGAGTTGATGGAGTGCTTATGAGACTAAGAGACACTCGAATGCATTGTGTAT
                                                        *** ************** ** ****** **.*.******** ** ******* :*

L.usitatissimum-Lus10026859                         TTAGTGACAGTCCCAAGCCTGTCATCCTTCGCGAAAGTTGCTGGAGACAAGCAACCTTCG
Textile-hemp-csa_locus_3380_iso_1_len_1164_ver_2    TTGGTGACAAGTCAAATCCAACTATTCTTCGAGAAAGCTGTTGGAGAGAAGCTACATTTC
H.lupulus-HL.Tea.v1.0.G021288.1                     TTGGTGACAATTCAAATCCAACTATTCTTCGAGAAAGCTGCTGGAGAGAGGCTACATTTC
Marijuana-scaffold76878                             TTGGTGACAAtTCAAATCCAACTATTCTTCGAGAAAGCTGcTGGAGAGAAGCTACATTTC
                                                    **.******.  *.** **:.  ** *****.***** ** ****** *.**:**.**
```

```
L.usitatissimum-Lus10026859                       AGGCTCTGTCTGCTAAAGGG-TACCCCATTGATGACTCTGCTTACAGTGATCCTAACAAC
Textile-hemp-csa_locus_3380_iso_1_len_1164_ver_2  AATCTTTATCTGCAA-AAGGATACCCTTCTGATTCTGCTGCTTACAGTGACCCCAGCTCC
H.lupulus-HL.Tea.v1.0.G021288.1                   AATATTTATCTGCAGAAAGGATACCCTTCTGATTCTGCTGCTTACAGCGACCCTAGCATC
Marijuana-scaffold76878                           AATCTTTATCTGCAA-AAGGATACCCTTCTGATTCTGCTGCTTACAGTGACCCCAGtgCC
                                                  *. .* *.*****:. *.** ***** : **** .  ********** ** ** *.   *

L.usitatissimum-Lus10026859                       ATCAGCCACAGGCTTCCAGTCGTTATGCATAGGACTCAAAAGCTTATAGGTCCCTTGTAA
Textile-hemp-csa_locus_3380_iso_1_len_1164_ver_2  ATCAGCCAGAGACTACCCATAGTCATGCACAAGTCTGAAGTGCTTAAAGT-CCCTGGTAA
H.lupulus-HL.Tea.v1.0.G021288.1                   ATCAGCCAGAGACTTCCCATTGTCATGCACAAGTCTGAAGTGCTTAAAGT-CCCTGGTAA
Marijuana-scaffold76878                           ATCAGCCAGAGACTACCCATtGTCATGCACAAGTCTGAAGTGCTTAAAGT-CCCcGGTAA
                                                  ******** **.**:**..* ** ***** *.*:** **.:*****:**  ***  ****

L.usitatissimum-Lus10026859                       ------------------------------------------------------------
Textile-hemp-csa_locus_3380_iso_1_len_1164_ver_2  CCTCTAGAGCTGTAAAAAATTATATGCACGGATGATTGTTGAATTTAGTCTTTCATTGTG
H.lupulus-HL.Tea.v1.0.G021288.1                   CCTCTAGAGCTGTAAAA-ATTATATGCACGGAAGATTGTTGAATTTAGCCTTTCATTATG
Marijuana-scaffold76878                           CCTCTAGAGCTGTAAAA-ATTATATGCACGGAaGATTGTTGAATTTAGTCTTTCATTGTG

L.usitatissimum-Lus10026859                       ------------------------------------------------------------
Textile-hemp-csa_locus_3380_iso_1_len_1164_ver_2  GTTCTGGTTGATGTTAAACTTATTTATTTACGAAAAGAAAAA-----------TCGACAT
H.lupulus-HL.Tea.v1.0.G021288.1                   GTTCTGGTTGATGTTAAACTTATTTATTTACAAAAAAGGAAAAAAATGGAATAAACGACT
Marijuana-scaffold76878                           GTTCTGGTTGATGTTAAACTTATTTATTTACGAAAAGAAAA---A--------TtGgCAT

L.usitatissimum-Lus10026859                       ------------------------------------------
Textile-hemp-csa_locus_3380_iso_1_len_1164_ver_2  ACATTTTCTTGTAAAAAAATAAGTTTAACATCAACCAGAACC
H.lupulus-HL.Tea.v1.0.G021288.1                   ACATTTTCTTGTAA----------------------------
Marijuana-scaffold76878                           ACATTTTCTTGTAA----------------------------
```

Tubulin

```
L.usitatissimum-Lus10031032                         ------------------------------------------------------------
H.lupulus-HL.SW.v1.0.G004672.1                      ------------------------------------------------------------
Textile-hemp-csa_locus_11261_iso_1_len_1086_ver_2   GCTCAATCATATTTTCTCTTGTAAAAAAAAGTAACCGAAAAACCTAAAACAGAAGAAAAA
Marijuana-scaffold23181                             GCTCAATCATATTTTCTCTTGTAAAAAAAAGTAACCGAAAAACCTAAAACAGAAGAAAAA

L.usitatissimum-Lus10031032                         --------------------------ATGAGGGAATGCATTTCCATCCACATCGGTCAGG
H.lupulus-HL.SW.v1.0.G004672.1                      ------------------------------------------------------------
Textile-hemp-csa_locus_11261_iso_1_len_1086_ver_2   GCAAAACCTAAAACCAGAGAAGAGAAATGAGAGAGTGCATCTCGATCCACATTGGTCAGG
Marijuana-scaffold23181                             GCAAAACCTAAAACCAGAGAAGAGAAATGAGAGAGTGCATCTCGATCCACATTGGTCAGG

L.usitatissimum-Lus10031032                         CCGGTATCCAGGTCGGAAACGCCTGCTGGGAGCTTTACTGCCTCGAGCATGGAATTCAGC
H.lupulus-HL.SW.v1.0.G004672.1                      ------------------------------------------------------------
Textile-hemp-csa_locus_11261_iso_1_len_1086_ver_2   CCGGAATCCAAGTCGGTAACTCCTGTTGGGAGCTCTATTGCCTTGAACATGGCATTCAGC
Marijuana-scaffold23181                             CCGGAATCCAAGTCGGTAACTCCTGTTGGGAGCTCTATTGCCTTGAACATGGCATTCAG-

L.usitatissimum-Lus10031032                         CTGACGGTCAGATGCCGAGTGATAAAACCGTAGGCGGAGGAGACGATGCTTTCAACACCT
H.lupulus-HL.SW.v1.0.G004672.1                      ------------------------------------------------------------
Textile-hemp-csa_locus_11261_iso_1_len_1086_ver_2   CCGATGGTCAAATGCCCAGTGACAAGACCGTTGGAGGTGGAGACGATGCCTTCAACACGT
Marijuana-scaffold23181                             ------------------------------------------------------------

L.usitatissimum-Lus10031032                         TTTTCAGTGAAACTGGTGCAGGGAAGCACGTTCCTCGCGCTGTGTTTGTCGATCTTGAGC
H.lupulus-HL.SW.v1.0.G004672.1                      ------------------------------------------------------------
Textile-hemp-csa_locus_11261_iso_1_len_1086_ver_2   TTTTCAGTGAAACCGGCGCCGGAAAGCATGTTCCCCGTGCTGTGTTTTTGGATCTAGAAC
Marijuana-scaffold23181                             ------------------------------------------------------------

L.usitatissimum-Lus10031032                         CCACTGTCATCGACGAGGTGAGGACTGGAAGCTACCGCCAGCTCTTCCACCCTGAGCAAC
H.lupulus-HL.SW.v1.0.G004672.1                      ------------------------------------------------------------
Textile-hemp-csa_locus_11261_iso_1_len_1086_ver_2   CGACGGTCATCGATGAAGTGAGGACCGGTACTTACCGCCAGCTTTTCCATCCCGAACAGC
Marijuana-scaffold23181                             ------------------------------------------------------------

L.usitatissimum-Lus10031032                         TTATCAGCGGCAAGGAAGACGCTGCCAACAACTTTGCTCGTGGCCACTACACAATTGGTA
H.lupulus-HL.SW.v1.0.G004672.1                      --------------------------------------------------------GGGA
Textile-hemp-csa_locus_11261_iso_1_len_1086_ver_2   TTATTAGCGGGAAGGAAGACGCAGCTAATAACTTCGCTAGAGGCCATTACACAATTGGGA
Marijuana-scaffold23181                             ------------------------------------------------------------

L.usitatissimum-Lus10031032                         AAGAGATTGTTGACCTGTGCTTGGACCGCATCAGGAAGCTCGCTGACAACTGCACTGGTC
H.lupulus-HL.SW.v1.0.G004672.1                      AAGAGATTGTTGACCTCTGTTTGGACCGGATCCGAAAGCTCGCCGATAACTGTACTGGTC
Textile-hemp-csa_locus_11261_iso_1_len_1086_ver_2   AAGAGATTGTAGATCTATGCCTTGATAGAATCCGAAAGCTTGCTGATAACTGTACTGGGC
Marijuana-scaffold23181                             ------------------------ATAGAATCCGAAAGCTTGCTGATAACTGTACTGGGC
                                                                            * .* ***.*.***** ** ** ***** ***** *

L.usitatissimum-Lus10031032                         TCCAAGGTTTCTTGGTGTTCAATGCAGTCGGTGGTGGTACTGGATCTGGTCTTGGATCCC
H.lupulus-HL.SW.v1.0.G004672.1                      TTCAAGGCTTTTTGGTCTTCAATGCCGTCGGTGGTGGTACCGGATCTGGACTTGGATCCC
Textile-hemp-csa_locus_11261_iso_1_len_1086_ver_2   TTCAAGGGTTTCTGGTTTTTCACGCTGTTGGCGGTGGAACTGGATCTGGTCTTGGATCTC
Marijuana-scaffold23181                             TTCAAGGGTTTCTGGTTTTTCACGCTGTTGGCGGTGGAACTGGATCTGGTCTTGGATCTC
                                                    * ***** **  **** ** .* ** ** ** *****:** ********:******** *

L.usitatissimum-Lus10031032                         TCCTCTTGGAGCGTCTCTCTGTGGACTATGGAAAGAAGTCCAAGCTCGGGTTCACCGTTT
H.lupulus-HL.SW.v1.0.G004672.1                      TTCTGCTGGAGCGTCTCTCTGTTGACTACGGAAAGAAATCGAAGCTGGGTTTCACTGTTT
Textile-hemp-csa_locus_11261_iso_1_len_1086_ver_2   TACTTCTTGAGAGACTCTCTGTTGATTATGGAAAAAAATCTAAACTTGGGTTTACAGTTT
Marijuana-scaffold23181                             TACTTCTTGAGAGACTCTCTGTTGATTATGGAAAAAAATCTAAACTTGGGTTTACgGTTT
                                                    * **  * ***.*:******** ** ** *****.**.** **.** ** ** ** ****

L.usitatissimum-Lus10031032                         ATCCATCGCCTCAAGTGTCAACCTCTGTTGTGGAGCCTTACAACAGTGTCCTCTCAACCC
H.lupulus-HL.SW.v1.0.G004672.1                      ACCCTTCTCCACAGGTGTCCACATCGGTGGTGGAGCCTTACAACAGTGTTCTCTCGACTC
Textile-hemp-csa_locus_11261_iso_1_len_1086_ver_2   ACCCATCTCCACAGGTTTCCACCTCTGTGGTTGAACCTTACAACAGTGTTTTGTCTACTC
Marijuana-scaffold23181                             ACCCATCTCCACAGGTTTCCACCTCTGTGGTTGAACCTTACAACAGTGTTTTGTCTACTC
                                                    * **:** **:**.** **.**.** ** ** **.**************  * ** ** *

L.usitatissimum-Lus10031032                         ATTCCCTCCTTGAACACACCGATGTTGCTGTTCTTCTGGACAATGAAGCCATCTATGACA
H.lupulus-HL.SW.v1.0.G004672.1                      ATTCCCTTCTTGAACACACCGACGTGGCTGTGCTTCTCGACAACGAAGCCATCTACGATA
Textile-hemp-csa_locus_11261_iso_1_len_1086_ver_2   ACTCTCTTTTGGAACACACAGATGTGTCTGTTCTTCTTGATAATGAGGCTATCTACGACA
Marijuana-scaffold23181                             ACTCTCTTTTGGAACACACAGATGTGTCTGTTCTTCTTGATAATGAGGCTATCTACGACA
                                                    * ** **  * ********.** **  **** ***** ** ** **.** ***** ** *

L.usitatissimum-Lus10031032                         TCTGCAGGCGCTCTCTTGACATTGAGCGACCCACCTACCACAATTTGAACCGTCTGATCT
H.lupulus-HL.SW.v1.0.G004672.1                      TCTGCCGTCGATCACTCGACATCGAACGTCCCACTTACACCAACCTCAATCGCCTTGTCT
Textile-hemp-csa_locus_11261_iso_1_len_1086_ver_2   TTTGCAGAAAGTCACTTGATATTGAAAGACCCACTTACACTAACCTTAACAGGCTTGTAT
Marijuana-scaffold23181                             TTTGCAGAAAaTCACTTGATATTGAAcGACCCACTTACACTAACCTTAACAGGCTTGTAT
                                                    * ***.*  .. **:** ** ** **..*:***** ***.. **  * ** .* ** .*.*

L.usitatissimum-Lus10031032                         CTCAGGTGATCTCATCCCTCACTGCATCTCTAAGGTTCGATGGTGCTCTCAATGTGGATG
H.lupulus-HL.SW.v1.0.G004672.1                      CCCAGGTGATCTCATCTCTCACAGCCTCTCTCCGGTTCGATGGAGCGCTCAATGTCGATG
Textile-hemp-csa_locus_11261_iso_1_len_1086_ver_2   CTCAGGTTATTTCTTCACTCACTGCTTCTCTTCGATTCGATGGAGCTCTTAATGTGGATG
Marijuana-scaffold23181                             CTCAGGTTATTTCTTCACTCACTGCTTCTCTTCGATTCGATGGAGCTCTTAATGTGGATG
                                                    * ***** ** **:** *****:** *****  .*.********:** ** ***** ****

L.usitatissimum-Lus10031032                         TTACAGAGTTCCAGACCAACTTGGTCCCGTATCCTAGGATCCACTTCATGCTTTCCTCAT
H.lupulus-HL.SW.v1.0.G004672.1                      TCACAGAGTTCCAGACTAACCTGGTTCCATACCCAAGAATCCATTTCATGCTCTCGTCGT
Textile-hemp-csa_locus_11261_iso_1_len_1086_ver_2   TGACTGAGTTTCAAACCAATCTTGTCCCTTACCCCAGAATCCATTTCATGCTTTCGTCAT
Marijuana-scaffold23181                             TGACTGAGTTTCAAACtAATCTTGTCCCTTACCCtAGAATCCATTTCATGCTTTCGTCAT
                                                    * **:***** **.** **  * ** ** ** ** **.***** ******** ** **.*

L.usitatissimum-Lus10031032                         ATGCTCCAGTCATCTCGGCTGAGAAGGCCTACCATGAGCAGCTGTCCGTTGCTGAGATCA
H.lupulus-HL.SW.v1.0.G004672.1                      TTGCCCCTGTGATCTCAGCCGAGAAGGCCTACCACGAGCAACTATCCGTGGCGGAGATCA
Textile-hemp-csa_locus_11261_iso_1_len_1086_ver_2   ATGCACCTGTGATTTCTGCAGAGAAAGCTTACCACGAGCAGCTTTCGGTGTCTGAAATCA
Marijuana-scaffold23181                             ATGCgCCTGTGATTTCTGCAGAGAAAGCTTACCACGAGCAaCTTTCGGTGTCTGAAATCA
                                                    :*** **:** ** ** ** *****.** ***** *****.** ** **  * **.****

L.usitatissimum-Lus10031032                         CCAACAGTGCCTTCGAGCCCTCTTCTATGATGGCCAAGTGTGACCCTCGCCATGGCAAGT
H.lupulus-HL.SW.v1.0.G004672.1                      CCAACAGCGCTTTCGAGCCATCTTCCATGATGGCCAAGTGCGACCCCCGCCATGGGAAAT
Textile-hemp-csa_locus_11261_iso_1_len_1086_ver_2   CAAACAGCGCTTTTGAACCATCTTCAATGATGGCCAAATGTGATCCTCGACATGGAAAGT
Marijuana-scaffold23181                             CAAACAGCGCTTTTGAACCA----------------------------------------
                                                    *.***** ** ** **.**.
```

```
L.usitatissimum-Lus10031032                          ACATGGCTTGTTGTCTGATGTACCGTGGTGACGTGGTTCCCAAAGATGTCAACGCAGCTG
H.lupulus-HL.SW.v1.0.G004672.1                       ACATGGCCTGCTGTTTGATGTACCGGGGTGACGTCGTTCCCAAAGATGTAAACGCCGCCG
Textile-hemp-csa_locus_11261_iso_1_len_1086_ver_2    ACATGGCTTGTTGTTTGATGTACAGGGGAGATGTTGTACCAAAGGATGTGAACGCAGCAG
Marijuana-scaffold23181                              ------------------------------------------------------------

L.usitatissimum-Lus10031032                          TGGCGACCATCAAGACAAAGAGGACCATCCAGTTCGTGGACTGGTGCCCAACTGGATTCA
H.lupulus-HL.SW.v1.0.G004672.1                       TGGCCA------------------------------------------------------
Textile-hemp-csa_locus_11261_iso_1_len_1086_ver_2    TCGCCA------------------------------------------------------
Marijuana-scaffold23181                              ------------------------------------------------------------

L.usitatissimum-Lus10031032                          AGTGTGGTATCAACTACCAGCCACCAACTGTTGTCCCAGGAGGCGACCTTGCCAAGGTGC
H.lupulus-HL.SW.v1.0.G004672.1                       ------------------------------------------------------------
Textile-hemp-csa_locus_11261_iso_1_len_1086_ver_2    ------------------------------------------------------------
Marijuana-scaffold23181                              ------------------------------------------------------------

L.usitatissimum-Lus10031032                          AGAGGGCTGTGTGCATGATCTCCAACTCCACCAGTGTGGCAGAGGTGTTTGGGCGAATTG
H.lupulus-HL.SW.v1.0.G004672.1                       ------------------------------------------------------------
Textile-hemp-csa_locus_11261_iso_1_len_1086_ver_2    ------------------------------------------------------------
Marijuana-scaffold23181                              ------------------------------------------------------------

L.usitatissimum-Lus10031032                          ACCACAAGTTTGATCTCATGTACGCCAAGCGTGCATTCGTCCACTGGTATGTGGGTGAGG
H.lupulus-HL.SW.v1.0.G004672.1                       ------------------------------------------------------------
Textile-hemp-csa_locus_11261_iso_1_len_1086_ver_2    ------------------------------------------------------------
Marijuana-scaffold23181                              ------------------------------------------------------------

L.usitatissimum-Lus10031032                          GTATGGAGGAAGGAGAGTTCTCTGAGGCACGTGAGGATCTGGCTGCTCTTGAGAAGGACT
H.lupulus-HL.SW.v1.0.G004672.1                       ------------------------------------------------------------
Textile-hemp-csa_locus_11261_iso_1_len_1086_ver_2    ------------------------------------------------------------
Marijuana-scaffold23181                              ------------------------------------------------------------

L.usitatissimum-Lus10031032                          ACGAGGAGGTTGGCGCTGAGGCTCCTGAGGATGGAGAAGATGAAGGTGATGAGTACTGA
H.lupulus-HL.SW.v1.0.G004672.1                       -----------------------------------------------------------
Textile-hemp-csa_locus_11261_iso_1_len_1086_ver_2    -----------------------------------------------------------
Marijuana-scaffold23181                              -----------------------------------------------------------
```

CDPK

```
L.usitatissimum-Lus10028862                         ------------------------------------------------------------
Textile-hemp-csa_locus_8286_iso_2_len_2019_ver_2    AAAAAAACCAAAACTGCTCAAGACCCATTTTTTCTCT------------CTCTATTTCTC
H.lupulus-HL.SW.v1.0.G043506.1                      ------------------------------------------------------------
Marijuana-scaffold109107                            AAAAAAACCAAAACTGCTCAAGACCCATTTTTTCTCTCTCtctctctctctATTTCTC

L.usitatissimum-Lus10028862                         ------------------------------------------------------------
Textile-hemp-csa_locus_8286_iso_2_len_2019_ver_2    CATTACCATATACCCTTTTTCTGTTTTCGTTTTTTCTTTTCTTTTATCCATATGGGGTGG
H.lupulus-HL.SW.v1.0.G043506.1                       ------------------------------------------------------------
Marijuana-scaffold109107                            CATTACCATATACCCTTTTTCTGTTTTCGTTTTTTCTTTTCTTTTATCCATATGGGGTGG

L.usitatissimum-Lus10028862                         ------------------------------------------------------------
Textile-hemp-csa_locus_8286_iso_2_len_2019_ver_2    CTTTGCTTCTCTTTGATTTGATCAAGCTTGTTATGGTTCGTCTTGTAAAGT---------
H.lupulus-HL.SW.v1.0.G043506.1                      -----------------------------GTTATAGTTCTTCTTGTCAAGTTTTTGCAAA
Marijuana-scaffold109107                            CTTTGCTTCTtTTTGATTTGATCAAGCTTGTTATGGTTCGTCTTGTAAAG---------T

L.usitatissimum-Lus10028862                         ------------------------------------------------------------
Textile-hemp-csa_locus_8286_iso_2_len_2019_ver_2    TTTGAACTTTGGTGTGAAAAGGGGTTTGACTTGAATATGCTGAAGAGTGGATCGTATGTA
H.lupulus-HL.SW.v1.0.G043506.1                      TTCGAACTGTGGTGTGAAAAGGGGTTTGACTTGAATATGCTGAAGACTGGATCGTATGTA
Marijuana-scaffold109107                            TTTGAACTTTGGTGTGAAAAGGGGTTTGACTTGAATATGCTGAAGAGTGGATCGTATGTA

L.usitatissimum-Lus10028862                         ------------------------------------------------------------
Textile-hemp-csa_locus_8286_iso_2_len_2019_ver_2    CTGTGTAAAGAGTTTAAATTGGATTAGATCTTGGACTT-AAACTAAGATTGTTAAGGAAG
H.lupulus-HL.SW.v1.0.G043506.1                      CTGTGTCAACGGGTTGAATTGGATTAGATCTTGGAGTTAAAACTAAGAGTGTTGAGGAGG
Marijuana-scaffold109107                            CTGTGTAAAGAGTTTAAATTGGATTAGATCTTGGACTT-AAACTAAGAcTGTTAAGGAAG

L.usitatissimum-Lus10028862                         -------------------ATGGGTAATAATTGCGTTGGACCAAGCGCTACCAAGAATGG
Textile-hemp-csa_locus_8286_iso_2_len_2019_ver_2    AACTAAAGAGTTGGATTAAATGGGGAATACTTGTGTTGGTCCTACCATCAACAAGAATGG
H.lupulus-HL.SW.v1.0.G043506.1                      AACT-AAGAGTTGGATTAAATGGGGAATACTTGTGTTGGTCCTACCATCAACAAGAATGG
Marijuana-scaffold109107                            AACTAAAGAGTTGGATTAAATGGGGAATACTTGTGTTGGTCCTACCATCAACAAGAATGG
                                                                       ***** ****.*** *****:**:* *.   *.*********

L.usitatissimum-Lus10028862                         CTTTCTCCAGACAGTTTCCGCTGCAATGTGGAGATCCAGATTGCCAGATGATGCAGCAGC
Textile-hemp-csa_locus_8286_iso_2_len_2019_ver_2    ATTCTTTCAATCCGTTTCAGCTGCAATGTGGCGGAGCAGATCGCCAGATGACTCTGTTTC
H.lupulus-HL.SW.v1.0.G043506.1                      ATTCTTTCAATCCGTTTCAGCTGCAATGTGGCGGTCCAGATCTCCGGATGACTCAATTTC
Marijuana-scaffold109107                            ATTCTTTCAATCCGTTTCAGCTGCAATGTGGCGGAGCAGATCGCCAGATGACTCTGTTTC
                                                    .**   * **.:*.*****.************.*.: *****  **.*****  *:. : *

L.usitatissimum-Lus10028862                         AGCAGCTGCTGCTCAGGGAAATGGTGAAACTGTGACTGATAAAGAAGTGCCTTTCGAGGT
Textile-hemp-csa_locus_8286_iso_2_len_2019_ver_2    AGAACATACT---------AATGGAGAAAGTGT------TCATGATGTGGCATCTAAGGA
H.lupulus-HL.SW.v1.0.G043506.1                      TGAACATACT---------AATGGAGAAAGTGC------CCATGATGTGGCATCTAAGGA
Marijuana-scaffold109107                            AGAACATACT---------AATGGAGAAAGTGT------TCATGATGTGGCATCTAAGGA
                                                    :*.* .*.**         *****:**** **        .*:**:*** *:*  .***:

L.usitatissimum-Lus10028862                         ---------------------TCAGGATAAGCCTCCAGAGCAAGTTACCATCCCTAAACA
Textile-hemp-csa_locus_8286_iso_2_len_2019_ver_2    GCCTGAATCACCTTTGCCAGTCCAAAACAAACCACCAGAACCTGTGATAATTCCAAAACC
H.lupulus-HL.SW.v1.0.G043506.1                      GCCTGAATCACCTATGCCAGTCCAAAACAAACCACCAGAGCCAGTTATAATTCCGAAACC
Marijuana-scaffold109107                            GCCTGAATCACCTTTGCCAGTCCAAAACAAACCACCAGAACCTGTGATAATTCCAAAACC
                                                                            **..* **.**:*****.*.:** * .** ** ****.

L.usitatissimum-Lus10028862                         GGAGAAAAGAGAGGAGCCGCCTCCTCAACAACAACAACAACAACAACAACAACAACCAGC
Textile-hemp-csa_locus_8286_iso_2_len_2019_ver_2    TGA---------------------------------------------------ACCT--
H.lupulus-HL.SW.v1.0.G043506.1                      TGA---------------------------------------------------ACCT--
Marijuana-scaffold109107                            TGA---------------------------------------------------ACCT--
                                                     **                                                    ***:

L.usitatissimum-Lus10028862                         AGTTCAGAAGCAGCCTGAACCAACCGAGAAGCCCAAGAGGGTGTTGCCTGAGATGAAGAG
Textile-hemp-csa_locus_8286_iso_2_len_2019_ver_2    -G---TGAAGGATCCA---CAATCCCCGAAAACCGAC---AAGTCCCCACATATGAAGAG
H.lupulus-HL.SW.v1.0.G043506.1                      -A---CAAAGGATCCA---CAATCCCCGAAAGCCGAG---AAGTCTCCACATATGAAGAG
Marijuana-scaffold109107                            -G---TGAAGGATCCA---CAATCCCCGAAAACCGAC---AAGTCCCCACATATGAAGAG
                                                     .    .*** * **:   *.*:** .***. **.*    .:**  **: * ********

L.usitatissimum-Lus10028862                         AGTTGGCAGCGCTGGTCTTCGAGTTGGTTCGGTTTTGCAAACAAGGACTGGAAATTTGAA
Textile-hemp-csa_locus_8286_iso_2_len_2019_ver_2    GGTGTCCAGTGCTGGACTTCGAGCTGGTTCGGTGTTGCAAACAAAAACTGGTAAATTGAA
H.lupulus-HL.SW.v1.0.G043506.1                      GGTGTCCAGTGCTGGACTTCGGGCTGGTTCGGTGTTGCAAACAAAAACTGGTAAATTAAA
Marijuana-scaffold109107                            GGTGTCCAGTGCTGGACTTCGAGCTGGTTCGGTGTTGCAAACAAAAACTGGTAAATTaAA
                                                    .**   *** *****:*****.* ********* **********..*****:**:**.**

L.usitatissimum-Lus10028862                         GGAGTTCTTCACTATGGGGAAGAAGCTTGGACAAGGGCAGTTCGGAACCACGTTTCTATG
Textile-hemp-csa_locus_8286_iso_2_len_2019_ver_2    AGAGTTCTACACTTTTGGGAAGAAACTAGGCCAAGGGCAATTCGGGACAACATTTCTGTG
H.lupulus-HL.SW.v1.0.G043506.1                      AGAGTTCTACACTTTTGGGAAGAAACTAGGCCAAGGGCAATTCGGGACAACATTTCTGTG
Marijuana-scaffold109107                            AGAGTTCTACACTTTTGGGAAGAAACTAGGCCAAGGGCAATTCGGGACAACATTTCTGTG
                                                    .*******:****:* ********.**:**.********.*****.**.**.*****.**

L.usitatissimum-Lus10028862                         CGTGGAGAAAGCTACTGGGAACAAATTTGCTTGTAAGTCGATTGCCAAGAGGAAGTTGTT
Textile-hemp-csa_locus_8286_iso_2_len_2019_ver_2    TGTGGAGAAGGCAACAGGGAAAGAGTATGCTTGCAAATCCATTGCGAAAAGGAAGTTAGT
H.lupulus-HL.SW.v1.0.G043506.1                      TGTAGAGAAGGCAACAGGAAAAGAGTATGCTTGCAAATCAATTGCAAAGAGGAAGTTAGT
Marijuana-scaffold109107                            TGTGGAGAAGGCAACAGGGAAAGAGTATGCTTGCAAATCCATTGCGAAAAGGAAGTTAGT
                                                     **.*****.**:**:**.**..*.*:****** **.** ***** **.********. *

L.usitatissimum-Lus10028862                         GACGGATGATGATGTGGAGGATGTGAGAAGGGAGATTCAGATAATGCATCATTTGGCTGG
Textile-hemp-csa_locus_8286_iso_2_len_2019_ver_2    CACAGATGATGATGTGGAAGATGTAAGGAGGGAGATTCAGATAATGCACCATTTGGCTGG
H.lupulus-HL.SW.v1.0.G043506.1                      CACAGATGACGATGTGGAGGATGTAAGGAGGGAGATTCAGATAATGCACCATTTGGCGGG
Marijuana-scaffold109107                            CACAGATGATGATGTGGAAGATGTAAGGAGGGAGATTCAGATAATGCACCATTTGGCTGG
                                                     **.***** ********.*****.**.******************* ******** **

L.usitatissimum-Lus10028862                         CCATCCGAATGTTATATCCATTAAGGGGGCTTATGAGGACGCTGTGGCGGTACATGTCGT
Textile-hemp-csa_locus_8286_iso_2_len_2019_ver_2    GCACCCCAATGTGATATCTATTAAAGGTGCATATGAGGATGCTGTGGCAGTCCATGTTGT
H.lupulus-HL.SW.v1.0.G043506.1                      CCACCCCAATGTGATATCCATTAAAGGGGCGTATGAGGATGCTGTAGCAGTCCATGTTGT
Marijuana-scaffold109107                            GCACCCCAATGTGATATCTATTAAAGGTGCATATGAGGATGCTGTGGCAGTCCATGTTGT
                                                     ** ** ***** ***** *****.** ** ******** *****.**.**.***** **

L.usitatissimum-Lus10028862                         TATGGAGCTGTGCGCTGGGGGCGAACTTTTCGACAGGATTGTTCAACGTGGTCATTATAC
Textile-hemp-csa_locus_8286_iso_2_len_2019_ver_2    GATGGAGTTATGCGCTGGTGGCGAGTTGTTCGACAGGATTATCCAACGCGGGCATTACAC
H.lupulus-HL.SW.v1.0.G043506.1                      AATGGAGTTATGTGCAGGTGGCGAGTTGTTCGACAGGATTATCCAACGCGGGCATTACAC
Marijuana-scaffold109107                            GATGGAGTTATGCGCTGGTGGCGAGTTGTTCGACAGGATTATCCAACGCGGGCATTACAC
                                                     ****** *.** **:** *****. * ************.* ***** ** ***** **
```

```
L.usitatissimum-Lus10028862                      GGAGAGAAAAGCTGCACAGCTTACCAGGACGATAGTTGGAGTTGTTGAAGCTTGTCACTC
Textile-hemp-csa_locus_8286_iso_2_len_2019_ver_2 AGAGAGGAAAGCTGCTGACCTTACTAGGACTATTGTTGGAGTTCTGGAAGCCTGCCATTC
H.lupulus-HL.SW.v1.0.G043506.1                   AGAGAGGAAAGCGGCCGAGCTTACTAGGACTATAGTTGGAGTGCTGGAAGCCTGCCATTC
Marijuana-scaffold109107                         AGAGAGGAAAGCTGCTGACCTTACTAGGACTATTGTTGGAGTTCTGGAAGCCTGCCATTC
                                                 .*****.***** **  * ***** ***** **:********  * ***** ** ** **

L.usitatissimum-Lus10028862                      GCTTGGTGTGATGCATCGGGACCTTAAGCCGGAGAATTTTCTGTTTGTGAGTCAACAAGA
Textile-hemp-csa_locus_8286_iso_2_len_2019_ver_2 ATTGGGGGTTATGCATCGCGACCTTAAGCCTGAGAATTTTCTTTTTGTCAACCAGCAGGA
H.lupulus-HL.SW.v1.0.G043506.1                   ATTGGGGGTTATGCATCGCGACCTTAAGCCTGAAAATTTTCTTTTTGTCAACCAGCAGGA
Marijuana-scaffold109107                         ATTGGGGGTTATGCATCGCGACCTTAAGCCTGAGAATTTTCTTTTTGTCAACCAGCAGGA
                                                 . * ** ** ******** *********** **.******** ***** *. **.**.**

L.usitatissimum-Lus10028862                      GGATTCGTTGCTCAAAACTATTGATTTTGGACTATCAATCTTCTTCAAGCCAGGAGAAAG
Textile-hemp-csa_locus_8286_iso_2_len_2019_ver_2 GGATGCACTTCTCAAAACTATTGACTTTGGTTTATCTGTTTTCTTCAAGCCAGGAGAGAG
H.lupulus-HL.SW.v1.0.G043506.1                   GGATGCACTTCTCAAAACTATTGACTTTGGTTTATCTGTTTTTTTCAAGCCAGG------
Marijuana-scaffold109107                         GGATGCACTTCTCAAAACTATTGACTTTGGTTTATCTGTTTTCTTCAAGCCAGGAGAGAG
                                                 **** *. * ************** *****: ****:.* ** ***********

L.usitatissimum-Lus10028862                      GTATAGTGATGTAGTTGGCAGTCCATACTATGTGGCTCCTGAGGTTTTAAAGAAGCGTTA
Textile-hemp-csa_locus_8286_iso_2_len_2019_ver_2 GTTTAGTGATGTGGTTGGCAGCCCATATTACGTTGCACCAGAAGTCTTGCGCAAGCGGTA
H.lupulus-HL.SW.v1.0.G043506.1                   ------------------------------------------------------------
Marijuana-scaffold109107                         aTTTAGTGATGTGGTTGGCAGCCCATATTACGTTGCACCAGAAGTCTTGCGCAAGCGGTA

L.usitatissimum-Lus10028862                      TGGTGCCGAAGCTGATGTATGGAGTGCTGGTGTTATTCTCTACATTCTGTTAAGTGGAGT
Textile-hemp-csa_locus_8286_iso_2_len_2019_ver_2 TGGTCCAGAAGCAGATGTTTGGAGTGCTGGAGTGATTCTTTACATTCTGTTAAGTGGAGT
H.lupulus-HL.SW.v1.0.G043506.1                   ------------------------------------------------------------
Marijuana-scaffold109107                         TGGTCCAGAAGCAGATGTTTGGAGTGCTGGAGTGATTCTTTACATTCTGTTAAGTGGAGT

L.usitatissimum-Lus10028862                      GCCACCCTTCTGGGCAGAGACCGAGCAGGGAATATTTGAGCAGGTTCTCCAGGGTGATCT
Textile-hemp-csa_locus_8286_iso_2_len_2019_ver_2 GCCTCCATTTTGGGCTGAAAGTGAGCAAGGGATATTTGAAGAAGTGTTGCATGGTGACCT
H.lupulus-HL.SW.v1.0.G043506.1                   ------------------------------------------------------------
Marijuana-scaffold109107                         GCCTCCATTcTGGGCTGAAAGTGAGCAAGGGATATTTGAAGAAGTGTTGCATGGTGACCT

L.usitatissimum-Lus10028862                      TGACTTTGAATCTGAACCTTGGCCTAACATCTCCGAGAGTGCTAAGGATTTAGTTAGGAA
Textile-hemp-csa_locus_8286_iso_2_len_2019_ver_2 TGATTTTTCAACAGAACCGTGGCCTAGTATTTCTGAAGGTGCCAAAGATTTAGTGAGGAA
H.lupulus-HL.SW.v1.0.G043506.1                   ------------------------------------------------------------
Marijuana-scaffold109107                         cGATTTTTCAACAGAACCGTGGCCTAGTATTTCTGAAGGTGCCAAAGAcTTAGTaAGGAA

L.usitatissimum-Lus10028862                      AATGCTTGTCCGAGACCCCAGAAGGCGACTAACTGCACATGAAGTTCTCTGCCATCCATG
Textile-hemp-csa_locus_8286_iso_2_len_2019_ver_2 AATGCTTATTCGAGACCCTAGGAGGCGGATATCTGCACATGATGTTTTGTGCCATCCCTG
H.lupulus-HL.SW.v1.0.G043506.1                   ------------------------------------------------------------
Marijuana-scaffold109107                         AATGCTTATTCGAGACCCTAGGAGGCGGATATCTGCACATGATGTTTTGTGCCATCCCTG

L.usitatissimum-Lus10028862                      GGTACAAGAAGAAGGTGTAGCTCCTGACAAGCCTCTGGATTCTGCCGTACTAAGTCGTTT
Textile-hemp-csa_locus_8286_iso_2_len_2019_ver_2 GGTTCAAGTTGGTGGTGTGGCTCCTGACAAGCCTCTCGATTCTGCAATATTAAGTCGGTT
H.lupulus-HL.SW.v1.0.G043506.1                   ------------------------------------------------------------
Marijuana-scaffold109107                         GGTTCAAGTTGGTGGTGTGGCTCCTGACAAGCCTCTCGATTCTGCAATATTAAGTCGGTT

L.usitatissimum-Lus10028862                      GAAGCAATTCTCGGCTATGAACAAGCTCAAGAAAATGGCTCTTATTATCATTGCTGAGAG
Textile-hemp-csa_locus_8286_iso_2_len_2019_ver_2 GAAACAATTCTCTGCAATGAACAAGCTTAAGAAAATGGCTATTAGAGTTATTGCAGAGAG
H.lupulus-HL.SW.v1.0.G043506.1                   ------------------------------------------------------------
Marijuana-scaffold109107                         GAAACAATTCTCTGCAATGAACAAGCTTAAGAAAATGGCTATTAGAGTTATTGCAGAGAG

L.usitatissimum-Lus10028862                      TTTATCCGAAGAGGAAATTGCAGGCCTGAAGGAGATGTTCAAGATGATAGATGCTGATGG
Textile-hemp-csa_locus_8286_iso_2_len_2019_ver_2 CTTATCTGAAGAAGAAATTGCTGGCCTAAAAGAAATGTTCAAAATGATAGACACTGACAA
H.lupulus-HL.SW.v1.0.G043506.1                   ------------------------------------------------------------
Marijuana-scaffold109107                         CTTATCTGAAGAAGAAATTGCTGGCCTAAAAGAAATGTTCAAAATGATAGACACTGACAA

L.usitatissimum-Lus10028862                      AAGCGGCCAGATCACATTCGAAGAACTCAAGGCTGGACTAAAAAGAGTTGGTGCTACTCT
Textile-hemp-csa_locus_8286_iso_2_len_2019_ver_2 CAGTGGTTCAATCACTTTTGAAGAACTTAAGGCTGGTCTGAAAAGATTTGGAGCTAATCT
H.lupulus-HL.SW.v1.0.G043506.1                   ------------------------------------------------------------
Marijuana-scaffold109107                         CAGTGGTTCAATCACTTTTGAAGAACTTAAGGCTGGTCTGAAAAGATTTGGAGCTAATCT

L.usitatissimum-Lus10028862                      CAAGGAATCAGAAATCTACGATTTAATGCAAGCGGCAGATATAGACAACAGTGGCACCAT
Textile-hemp-csa_locus_8286_iso_2_len_2019_ver_2 TAAGGAATCCGAAATTTATGATTTAATGCAAGCAGCAGATGTAGATAACAGTGGAACAAT
H.lupulus-HL.SW.v1.0.G043506.1                   ------------------------------------------------------------
Marijuana-scaffold109107                         TAAGGAATCCGAAATTTATGATTTAATGCAAGCAGCAGATGTAGATAACAGTGGAACAAT

L.usitatissimum-Lus10028862                      TGATTACGCCGAGTTCATCGCTGCAACGTTGCACCTGAACAAAATCGAGAAAGAGGATCA
Textile-hemp-csa_locus_8286_iso_2_len_2019_ver_2 TGATTACGGAGAGTTTGTAGCAGCCACTTTGCATTTAAACAAAATTGAGAGAGAAGATCA
H.lupulus-HL.SW.v1.0.G043506.1                   ------------------------------------------------------------
Marijuana-scaffold109107                         TGATTACGGAGAGTTTGTAGCAGCCACTTTGCATTTAAACAAAATTGAGAGAGAAGATCA

L.usitatissimum-Lus10028862                      TCTATTTGCAGCCTTTTCGTACTTCGACAAGGATGGAAGTGGTTACATCACTCAAGACGA
Textile-hemp-csa_locus_8286_iso_2_len_2019_ver_2 TTTGTTTGCGGCATTTTCCTACTTTGATAAAGACGGAAGTGGCTACATTACTCAAGATGA
H.lupulus-HL.SW.v1.0.G043506.1                   ------------------------------------------------------------
Marijuana-scaffold109107                         TTTGTTTGCGGCATTTTCCTACTTTGATAAAGACGGAAGTGGCTACATTACTCAAGATGA

L.usitatissimum-Lus10028862                      GCTTCAACAAGCTTGCGAGGAGTTTGGCCTACAAGACGTCCGCTTGGAAGAGATGATCAA
Textile-hemp-csa_locus_8286_iso_2_len_2019_ver_2 GCTTCAACATGCTTGCGAGGAGTTTGGGGTAGAGGACGTCCGCCTAGAAGAGATGATGCA
H.lupulus-HL.SW.v1.0.G043506.1                   ------------------------------------------------------------
Marijuana-scaffold109107                         GCTTCAACATGCTTGCGAGGAGTTTGGGGTAGAGGACGTCCGCCTAGAAGAGATGATGCA

L.usitatissimum-Lus10028862                      CGAAGCTGATCAGGACAATGACGGGCGCATAGATTACAACGAATTTGTGGCGATGATGCA
Textile-hemp-csa_locus_8286_iso_2_len_2019_ver_2 AGAAGTTGATCAGGATAATGATGGACGCATAGATTATAACGAGTTTGTGGCCATGATGCA
H.lupulus-HL.SW.v1.0.G043506.1                   ------------------------------------------------------------
Marijuana-scaffold109107                         AGAAGTTGATCAGGATAATGATGGACGCATAGATTATAACGAGTTTGTGGCCATGATGCA

L.usitatissimum-Lus10028862                      GAAGGGAAACGTAGCACCAACCCCGGGAAGGAAAGGACTAGAGACTAGCTTCAGCATGGC
Textile-hemp-csa_locus_8286_iso_2_len_2019_ver_2 GAAAGGAACTATTGGT---GGCCCTGCTAAGAAGGGCCTACAAAATTCCTTCAGCATTGC
H.lupulus-HL.SW.v1.0.G043506.1                   ------------------------------------------------------------
```

```
Marijuana-scaffold109107                          GAAAGGAACTATTGGT---GGCCCTGCTAAGAAGGGCCTACAAAATTCCTTCAGCATTGC


L.usitatissimum-Lus10028862                       GTTCAAAGATGCGCGGAAAATCAGCACCAGCAAGTAG
Textile-hemp-csa_locus_8286_iso_2_len_2019_ver_2  TTTTAGAGAGGGTCGCAAACTT---------------
H.lupulus-HL.SW.v1.0.G043506.1                    -------------------------------------
Marijuana-scaffold109107                          TTTTAGAGAGGGTCGCAAACTT---------------
```

RAN

```
L.usitatissimum-Lus10025738                          ------------------------------------------------------------
Marijuana-scaffold6813                               --AAAGTATAGCTTTCACAACAGCAAAATATTTAACACTTTGGAGATCTGGAAAGGATTC
Textile-hemp-csa_locus_1455_iso_4_len_1015_ver_2     CAAAAGTATAGCTTTCACAACAGCAAAATATTTAACACTTTGGAGATCTGGAAAGGATTC
H.lupulus-HL.SW.v1.0.G039301.1                       ------------------------------------------------------------


L.usitatissimum-Lus10025738                          ------------------------------------------------------------
Marijuana-scaffold6813                               TCAATCTCTCTCTCTCTctATATATTCTCCGGTAAGTTCTAGAGAGAGAAAGGTGAAGTG
Textile-hemp-csa_locus_1455_iso_4_len_1015_ver_2     TCAATCTCTCTC--TCTCTATATATTCTCCGGTAAGTTCTAGAGAGAGAAAGGTGAAGTG
H.lupulus-HL.SW.v1.0.G039301.1                       ------------------------------------------------------------


L.usitatissimum-Lus10025738                          -----ATGGC---------TGGGTATGGAGATCCTAGTCAGAAAGTGGATTATGTCTTTA
Marijuana-scaffold6813                               TGAAAATGGCGAGCGGTGGAGGCTACGGCGATACGAGCCAGAAGATAGATTACGTGTTCA
Textile-hemp-csa_locus_1455_iso_4_len_1015_ver_2     TGAAAATGGCGAGCGGTGGAGGCTACGGCGATACGAGCCAGAAGATAGATTACGTGTTCA
H.lupulus-HL.SW.v1.0.G039301.1                       ------------------------------------------------------------


L.usitatissimum-Lus10025738                          AGGTGGTCCTGATTGGAGACTCGGCGGTGGGGAAGTCTCAGATACTTGCTAGATTTTCGA
Marijuana-scaffold6813                               AGGTTGTGtTGATCGGAGATTCGGCGGTGGGGAAGTCACAGATTCTGGCTCGGTTTGCTA
Textile-hemp-csa_locus_1455_iso_4_len_1015_ver_2     AGGTTGTGCTGATCGGAGATTCGGCGGTGGGGAAGTCACAGATTCTGGCTCGGTTTGCTA
H.lupulus-HL.SW.v1.0.G039301.1                       ------------------------------------------------------------


L.usitatissimum-Lus10025738                          GGAATGAGTTCAGTCTCGACTCCAAGGCCACCATCGGCGTCGAGTTCCAGACACGGACTA
Marijuana-scaffold6813                               GGAATGAGTTCAGCTTGGACTCGAAAGCCACCATCGGCGTTGAGTTCCAGACTAGGACTC
Textile-hemp-csa_locus_1455_iso_4_len_1015_ver_2     GGAATGAGTTCAGCTTGGACTCGAAAGCCACCATCGGCGTTGAGTTCCAGACTAGGACTC
H.lupulus-HL.SW.v1.0.G039301.1                       ------------------------------------------------------------


L.usitatissimum-Lus10025738                          TGGTTATACAGCACAAGAGCGTCAAGGCTCAGATCTGGGATACCGCCGGCCAAGAACGAT
Marijuana-scaffold6813                               TCGTCATCGAACACAAGAGTGTTAAGGCTCAGATCTGGGATACTGCAGGCCAGGAACGAT
Textile-hemp-csa_locus_1455_iso_4_len_1015_ver_2     TCGTCATCGAACACAAGAGTGTTAAGGCTCAGATCTGGGATACTGCAGGCCAGGAACGAT
H.lupulus-HL.SW.v1.0.G039301.1                       ---------------------------------------------------------GAT
                                                                                                              ***

L.usitatissimum-Lus10025738                          ACAGAGCGGTTACAAGTGCTTATTACAGAGGTGCGGTAGGGGCGATGTTGGTGTACGACA
Marijuana-scaffold6813                               ACAGAGCGGTGACGAGTGCATACTATAGGGGAGCTGTTGGGGCAATGCTTGTTTATGATA
Textile-hemp-csa_locus_1455_iso_4_len_1015_ver_2     ACAGAGCGGTGACGAGTGCATACTATAGGGGAGCTGTTGGGGCAATGCTTGTTTATGATA
H.lupulus-HL.SW.v1.0.G039301.1                       ACAGGGCAGTTACGAGTGCATATTATAGGGGAGCTGTTGGGGCAATGCTTGTTTATGACA
                                                     ****.**.** **.*****:** ** **.**:** **:*****.*** * ** ** ** *

L.usitatissimum-Lus10025738                          TAACCAAGCGCCAGACCTTTGATCACATTCCGCGCTGGCTGGAAGAGTTACGTGGTCATG
Marijuana-scaffold6813                               TAACCAAACGCCAGACCTTCGATCACATACCACGTTGGCTGGAAGAGCTACGTAACCATG
Textile-hemp-csa_locus_1455_iso_4_len_1015_ver_2     TAACCAAACGCCAGACCTTCGATCACATACCACGTTGGCTGGAAGAGCTACGTAACCATG
H.lupulus-HL.SW.v1.0.G039301.1                       TAACCAAACGGCAGACCTTCGATCACATACCGCGTTGGCTGGAAGAGCTACGTAACCACG
                                                     *******.** ******** ********:**.** ************ *****.. ** *

L.usitatissimum-Lus10025738                          CTGACAAGAATATTGTTATCATTCTGGTAGGGAACAAGTGTGATCTTGAGGAACAGAGAG
Marijuana-scaffold6813                               CTGACAAGAACATAGTCATCATTCTGATCGGAAACAAAACTGATtTAGAGAACCAGCGTG
Textile-hemp-csa_locus_1455_iso_4_len_1015_ver_2     CTGACAAGAACATAGTCATCATTCTGATCGGAAACAAAACTGATCTAGAGAACCAGCGTG
H.lupulus-HL.SW.v1.0.G039301.1                       CTGACAAGAACATAGTCATCATTCTGATTGGAAACAAAACTGATCTAGAGAACCAGCGTG
                                                     ********** **:** *********.* **.*****.: **** *:***.*.***.*:*

L.usitatissimum-Lus10025738                          GAGTACCCACAGAGGATGCCAAGGAATTTGCAGAGAAGGAGGGTTTGTTCTTCCTAGAAA
Marijuana-scaffold6813                               CAGTCCCCACTGAAGACGCCAAAGAATTTGCCCAGAAAGAAGGGCTTTTCTTTTTGGAGA
Textile-hemp-csa_locus_1455_iso_4_len_1015_ver_2     CAGTCCCCACTGAAGACGCCAAAGAATTTGCCCAGAAAGAAGGGCTTTTCTTTTTGGAGA
H.lupulus-HL.SW.v1.0.G039301.1                       CAGTCCCCACTGAAGACGCTAAAGAATTTGCTCAGAAAGAAGGGCTTTTCTTCTTGGAGA
                                                      ***.*****:**.** ** **.********  ****.**.**  * *****  *.**.*

L.usitatissimum-Lus10025738                          CCTCCGCATTGAACGCCATCAATGTGGAGAGTGCCTTTTCAACGGTATTGACGGAGATAT
Marijuana-scaffold6813                               CTTCAGCACTGGAATCAACTAATGTCGAGAACGCCTTCTTGACCGTGCTCACAGAGATTT
Textile-hemp-csa_locus_1455_iso_4_len_1015_ver_2     CTTCAGCACTGGAATCAACTAATGTCGAGAACGCCTTCTTGACCGTGCTCACAGAGATTT
H.lupulus-HL.SW.v1.0.G039301.1                       CTTCAGCACTGGAATCAACTAATGTCGAGAATGCCTTCTTGACCGTGCTGACAGAGATTT
                                                     * **.*** **.*. *.*  ***** ****. ***** * .** **. * **.*****:*

L.usitatissimum-Lus10025738                          TCAACATTGTAAACAAAAAGAGCTTGACAGCTGATGAGAATCAAGGCAACGGTAACACTG
Marijuana-scaffold6813                               TCAACATTGTGAACAAGAAGAACCTTGCGGCTGATGAAAACCAAGGAAATGGTAACCCTG
Textile-hemp-csa_locus_1455_iso_4_len_1015_ver_2     TCAACATTGTGAACAAGAAGAACCTTGCGGCTGATGAAAACCAAGGAAATGGTAACCCTG
H.lupulus-HL.SW.v1.0.G039301.1                       TCAACATTGTGAACAAGAAAAACCTTGCTGCTGATGAAAACCAAGGAAATGGTAACCCTG
                                                     **********.*****.**.*.* * .* ********.** *****.** ******.***

L.usitatissimum-Lus10025738                          CGTCATTAGCTGGTAAGAAAATCCTGGTGCCAGGCCCTGCACAAGTAATTCCAGCAAAGA
Marijuana-scaffold6813                               CATCCCTTACTGGCAAGAAGATCATCGTGCCGGGCCCTGCACAAGTTATCCCTGAAAAGA
Textile-hemp-csa_locus_1455_iso_4_len_1015_ver_2     CATCCCTTACTGGCAAGAAGATCATCGTGCCGGGCCCTGCACAAGTTATCCCTGAAAAGA
H.lupulus-HL.SW.v1.0.G039301.1                       CATCCCTTACTGGCAAGAAGATCATCGTCCCAGGTCCTGCACAGGTTATCCCAGAAAAGA
                                                     *.**. *:.**** *****.***.* ** **.** ********.**:** **:*.*****

L.usitatissimum-Lus10025738                          GAAACATGTGTTGCACCTCATCGTGA----------------------------------
Marijuana-scaffold6813                               ACAAGATGTGTTGTACATCATCGTGATCTAATCTTTTGCACAAAGTGG-TCTCAAATACA
Textile-hemp-csa_locus_1455_iso_4_len_1015_ver_2     ACAAGATGTGTTGTACATCATCGTGATCTAATCTTTTGCACAAAGTGG-TCTCAAATACA
H.lupulus-HL.SW.v1.0.G039301.1                       ACAAGATGTGTTGTACATCATCGTGATCTAATC-TTTGCACAAAGTGGATCTCAAATACA
                                                     ..** ******** **.*********

L.usitatissimum-Lus10025738                          ------------------------------------------------------------
Marijuana-scaffold6813                               AGATTCTTGTTTTCAAAATCTCTAAAATAGCAATTTTTTGATGGGTGGTGTTTTTTCATT
Textile-hemp-csa_locus_1455_iso_4_len_1015_ver_2     AGATTCTTGTTTTCAAAATCTCTAAAATAGCAATTTTTTGATGGGTGGTGTTTTTTCATT
H.lupulus-HL.SW.v1.0.G039301.1                       AGATTTATGTTTTCATAATCTCCAAAATAGCAATTTTTTGATGGGTGGTGCTTTTTCATT


L.usitatissimum-Lus10025738                          ------------------------------------------------------------
Marijuana-scaffold6813                               TGTATATTTTTACATTGTTTTAGCCTTTAGTTTGCATGGGCGGCCGAACTtCTTGCTCaT
Textile-hemp-csa_locus_1455_iso_4_len_1015_ver_2     TGTATATTTTTACATTGTTTTAGCCTTTAGTTTGCATGGGCGGCCGAACTACTTGCTCGT
H.lupulus-HL.SW.v1.0.G039301.1                       TGTATATGTTTACATTGTTTTAGCCTTTAGTTTGGATGGGCAGCTGAACTACTTGCCCGT


L.usitatissimum-Lus10025738                          -----------------------------------------------------------
Marijuana-scaffold6813                               TATGATAAGGAAAGAAAGAACTA-AGTTGAAGTTGAGTTGCA-----------------
Textile-hemp-csa_locus_1455_iso_4_len_1015_ver_2     TATGATAAGGAAAGAAAGAACTA-AGTTGAAGTTGAGTTGCAAGATCGGAAGAGCGGTT
H.lupulus-HL.SW.v1.0.G039301.1                       TATGACAAGGGAAGAAAGAACTAAAGTTGAAGCTGAGTTGCA-----------------
```

Clathrin

```
L.usitatissimum-Lus10040086                         ------------------------------------------------------------
Marijuana-scaffold38061                             ACAAAACacaatACAACTCAActcttCTCTTCTCTTCCATTTCTGCTTTGTGAAATTCTT
Textile-Hemp-csa_locus_5464_iso_6_len_1880_ver_2    ----------ACAAAACACAACTCAACTCTTCTCTTCCATTTCTGCTTTGTGAAATTCTT
H.lupulus-HL.SW.v1.0.G042778.1                      ------------------------------------------------------------

L.usitatissimum-Lus10040086                         ------------------------------------------------------------
Marijuana-scaffold38061                             CCATTTCTCCCTTACCAAGaAAAGCAGAGATCAAATCAAATAGATAGGCCATCCATCTTT
Textile-Hemp-csa_locus_5464_iso_6_len_1880_ver_2    CCATTTCTCCCTTACCAAGGAAAGCAGAGATCAAATCAAATAGATAGGCCATCCATCTTT
H.lupulus-HL.SW.v1.0.G042778.1                      ------------------------------------------------------------

L.usitatissimum-Lus10040086                         ------------------------------------------------------------
Marijuana-scaffold38061                             CAAATCTTACGTCCCAGATCGCAACTACTCCGCCTAGATCTGATTCcGGACGCCACCCAC
Textile-Hemp-csa_locus_5464_iso_6_len_1880_ver_2    CAAATCTTACGTCCCAGATCGCAACTACTCCGCCTAGATCTGATT-CGGACGCCACCCAC
H.lupulus-HL.SW.v1.0.G042778.1                      ------------------------------------------------------------

L.usitatissimum-Lus10040086                         ----------ATGCCGGTAGCTGCTTCAGCTCTCTATTTCTTGAACCTCCGCGGCGATGT
Marijuana-scaffold38061                             TGGATCCGTCATGCCGGTGGCTGCTTCCGCCATTTACTTCTTGAACCTCCGAGGCGATGT
Textile-Hemp-csa_locus_5464_iso_6_len_1880_ver_2    TGGATCCGTCATGCCGGTGGCTGCTTCCGCCATTTACTTCTTGAACCTCCGAGGCGATGT
H.lupulus-HL.SW.v1.0.G042778.1                      ------------------------------------------------------------

L.usitatissimum-Lus10040086                         CCTCATCAATCGTCTCTACCGCGACGATGTTGGGGGAAATATGGTTGATGCGTTCCGGAT
Marijuana-scaffold38061                             TCTCATCAATCGTCTCTATCGCGACGATGTTGGGGGAAATATGGTGGATGCTTTTCGAAC
Textile-Hemp-csa_locus_5464_iso_6_len_1880_ver_2    TCTCATCAATCGTCTCTATCGCGACGATGTTGGGGGAAATATGGTGGATGCTTTTCGAAC
H.lupulus-HL.SW.v1.0.G042778.1                      ------------------------------------------------------------

L.usitatissimum-Lus10040086                         GCATATTATGCAGACGAAGGAACTTGGGACATGCCCTGTGCGTCAAATTGGAGGCTGTTC
Marijuana-scaffold38061                             GCATATAATGCAAACTAAAGAACTtGGTACATGTCCTGTACGACAAATAGGGGGATGTTC
Textile-Hemp-csa_locus_5464_iso_6_len_1880_ver_2    GCATATAATGCAAACTAAAGAACTCGGTACATGTCCTGTACGACAAATAGGGGGATGTTC
H.lupulus-HL.SW.v1.0.G042778.1                      ------------------------------------------------------------

L.usitatissimum-Lus10040086                         ATTCTTTTATATGAGGATCAGCAACGTCTACATTGTGGTTGTAGTGAGCAGCAATGCTAA
Marijuana-scaffold38061                             TTTCTTCTACATGAGAATAAGCAACGTgTACATTGTGATTGTTGTCAGCAGCAATGCAAA
Textile-Hemp-csa_locus_5464_iso_6_len_1880_ver_2    TTTCTTCTACATGAGAATAAGCAACGTTTACATTGTGATTGTTGTCAGCAGCAATGCAAA
H.lupulus-HL.SW.v1.0.G042778.1                      ------------------------------------------------------------

L.usitatissimum-Lus10040086                         TGTTGCTTGTGCATTCAAATTTGTGGTAGAGGCTGTTGCACTATTCAAGTCATACTTCGG
Marijuana-scaffold38061                             TGTAGCTTGTGCGTTCAAGTTTGTTGTTGAGGCGGTTGCGTTGTTCAAATCATATTTTGG
Textile-Hemp-csa_locus_5464_iso_6_len_1880_ver_2    TGTAGCTTGTGCGTTCAAGTTTGTTGTTGAGGCGGTTGCGTTGTTCAAATCATATTTTGG
H.lupulus-HL.SW.v1.0.G042778.1                      ------------------------------------------------------------

L.usitatissimum-Lus10040086                         TGGTGCATTTGATGAAGATGCTATCAGAAATAATTTTGTTTTGATATACGAGTTGCTTGA
Marijuana-scaffold38061                             TGGGGCTTTTGATGAAGATGCAATTCGTAATAATTTTGTTCTGATTTATGAACTGTTAGA
Textile-Hemp-csa_locus_5464_iso_6_len_1880_ver_2    TGGGGCTTTTGATGAAGATGCAATTCGTAATAATTTTGTTCTGATTTATGAACTGTTAGA
H.lupulus-HL.SW.v1.0.G042778.1                      ------------------------------------------------------------

L.usitatissimum-Lus10040086                         TGAGATCATGGACTTTGGATATCCACAGAATTTGTCTCCTGAGATATTAAAGCTATACAT
Marijuana-scaffold38061                             TGAAATTATGGATTTTGGTTACCCcCAAAATCTTTCACCAGAAATTTTGAAGTTGTACAT
Textile-Hemp-csa_locus_5464_iso_6_len_1880_ver_2    TGAAATTATGGATTTTGGTTACCCTCAAAATCTTTCACCAGAAATTTTGAAGTTGTACAT
H.lupulus-HL.SW.v1.0.G042778.1                      ------------------------------------------------------------

L.usitatissimum-Lus10040086                         TACTCAAGAAGGAGTGCGGTCACCATTTTCATCCAAGCAAGCTTTGGATAAGCCTGTACC
Marijuana-scaffold38061                             TACTCAAGAAGGGGTTCGTTCGCCATTTTCTTCTAAGCCC---ACGGATAAGCCTGTTCC
Textile-Hemp-csa_locus_5464_iso_6_len_1880_ver_2    TACTCAAGAAGGGGTTCGTTCGCCATTTTCTTCTAAGCCC---ACGGATAAGCCTGTTCC
H.lupulus-HL.SW.v1.0.G042778.1                      ------------------------------------------------------------

L.usitatissimum-Lus10040086                         AAATGCCACATTGCAAGTTACAGGGGCTGTTGGTTGGCGAAGGGAAGGACTCGTATACAA
Marijuana-scaffold38061                             TAATGCAACTCTACAAGTTACGGGTGCTGTTGGTTGGCGGAGAGAAGGCCTTGTTTATAA
Textile-Hemp-csa_locus_5464_iso_6_len_1880_ver_2    TAATGCAACTCTACAAGTTACGGGTGCTGTTGGTTGGCGGAGAGAAGGCCTTGTTTATAA
H.lupulus-HL.SW.v1.0.G042778.1                      ------------------------------------------------------------

L.usitatissimum-Lus10040086                         AAAGAACGAGGTTTTCTTGGATATTGTGGAAAGCGTTAATCTTCTTATGTCCTCCAAAGG
Marijuana-scaffold38061                             AAAGAATGAGGTGTTTTTGGATATTGTGGAAAGTGTAAATCTTCTTATGTCTTCAAAAGG
Textile-Hemp-csa_locus_5464_iso_6_len_1880_ver_2    AAAGAATGAGGTGTTTTTGGATATTGTGGAAAGTGTAAATCTTCTTATGTCTTCAAAAGG
H.lupulus-HL.SW.v1.0.G042778.1                      ------------------------------------------------------------

L.usitatissimum-Lus10040086                         TAGTGTACTACGTTGTGATGTAACTGGGAAGATTCTGATGAAGTGCTTCCTCTCCGGAAT
Marijuana-scaffold38061                             TAGTGTTTTGCGTTGTGATGTAACTGGAAAGATCCTAATGAAGTGCTTTCTCTCTGGAAT
Textile-Hemp-csa_locus_5464_iso_6_len_1880_ver_2    TAGTGTTTTGCGTTGTGATGTAACTGGAAAGATCCTAATGAAGTGCTTTCTCTCTGGAAT
H.lupulus-HL.SW.v1.0.G042778.1                      ------------------------------------------------------------

L.usitatissimum-Lus10040086                         GCCTGATTTGAAATTGGGTTTAAACGATAAAATTGGCCTTGAGAAAGAGTCACAACTCAA
Marijuana-scaffold38061                             GCCTGATTTGAAGTTGGGTTTAAATGATAAGATTGGCCTTGAAAAAGAGTCGCAACTTAA
Textile-Hemp-csa_locus_5464_iso_6_len_1880_ver_2    GCCTGATTTGAAGTTGGGTTTAAATGATAAGATTGGCCTTGAAAAAGAGTCGCAACTTAA
H.lupulus-HL.SW.v1.0.G042778.1                      ------------------------------------------------------------

L.usitatissimum-Lus10040086                         GAACCGTCCCACTAAAAGTGGTAAAACAATTGAGCTTGATGATGTTACTTTCCATCAATG
Marijuana-scaffold38061                             ATCTCGTCCAACAAAAAGCGGTAAAACTATTGAGCTTGACGATGTTACCTTTCATCAATG
Textile-Hemp-csa_locus_5464_iso_6_len_1880_ver_2    ATCTCGTCCAACAAAAAGCGGTAAAACTATTGAGCTTGACGATGTTACCTTTCATCAATG
H.lupulus-HL.SW.v1.0.G042778.1                      ------------------------------------------------------------

L.usitatissimum-Lus10040086                         CGTGAACTTAACAAGGTTCAACTCGGAGAAGACAGTTAGTTTTGTTCCACCTGATGGTGA
Marijuana-scaffold38061                             TGTGAACTTGACGAGGTTTAATTCGGAGAAAACTGTCAGTTTTGTGCCACCAGATGGTGA
Textile-Hemp-csa_locus_5464_iso_6_len_1880_ver_2    TGTGAACTTGACGAGGTTTAATTCGGAGAAAACTGTCAGTTTTGTGCCACCAGATGGTGA
H.lupulus-HL.SW.v1.0.G042778.1                      ------------------------------------------------------------
```

```
L.usitatissimum-Lus10040086                        ATTTGAATTGATGAAGTACCGCATCACAGAGGGGGTTAATCTACCATTCAGGGTATTACC
Marijuana-scaffold38061                            ATTTGAATTGATGAAGT---------ATGAAGGTGTGAATCTTCCATTCCGAGTGtTGCC
Textile-Hemp-csa_locus_5464_iso_6_len_1880_ver_2   ATTTGAATTGATGAAGTACCGTATTACTGAAGGTGTGAATCTTCCATTCCGAGTGCTGCC
H.lupulus-HL.SW.v1.0.G042778.1                     ------------------------------------------------------------

L.usitatissimum-Lus10040086                        AACCATTAAGGAACATGGCCGAACACGTATGGAAGTGAATGTCAAGGTTAAGAGCGTTTT
Marijuana-scaffold38061                            TACTATCAAGGAACTTGGTAGAACACGCATGGAAGTAAATGTCAAGGTGAAGAGTGTCTT
Textile-Hemp-csa_locus_5464_iso_6_len_1880_ver_2   TACTATCAAGGAACTTGGTAGAACACGCATGGAAGTAAATGTCAAGGTGAAGAGTGTCTT
H.lupulus-HL.SW.v1.0.G042778.1                     ------------------------------------------------------------

L.usitatissimum-Lus10040086                        TGGGGCGAAAATGTTTGCTCTTGGTGTGGTAATCAAAATCCCTGTACCAAAACAAACAGC
Marijuana-scaffold38061                            TGGTGCAAAAATGTTTGCACTTGGGGTTGTCATTAAAATCCCCGTACCAAAACAAACAGC
Textile-Hemp-csa_locus_5464_iso_6_len_1880_ver_2   TGGTGCAAAAATGTTTGCACTTGGGGTTGTCATTAAAATCCCCGTACCAAAACAAACAGC
H.lupulus-HL.SW.v1.0.G042778.1                     ------------------------------------------------------------

L.usitatissimum-Lus10040086                        TAAAACAAGCTTCCAAGTGACGTCAGGACGGGCAAAGTACAATGCGTCAATTGATTGCTT
Marijuana-scaffold38061                            TAAAACGAGTTTTCAAGTTACATCTGGTCGATGTAAATATAATGCAGCTATTGATTGCTT
Textile-Hemp-csa_locus_5464_iso_6_len_1880_ver_2   TAAAACGAGTTTTCAAGTTACATCTGGTCGATGTAAATATAATGCAGCTATTGATTGCTT
H.lupulus-HL.SW.v1.0.G042778.1                     ------------------------------------------------------------

L.usitatissimum-Lus10040086                        GGTCTGGAAGATAAAGAAGTTTCCAGGTCAGACTGAGCCAACCTTGAGTGCAGAAGTTGA
Marijuana-scaffold38061                            AGTCTGGAAGATAAGAAAATTTCCTGGGCAAACTGAGCCAACAATGAGTGCAGAGGTTGA
Textile-Hemp-csa_locus_5464_iso_6_len_1880_ver_2   AGTCTGGAAGATAAGAAAATTTCCTGGGCAAACTGAGCCAACAATGAGTGCAGAGGTTGA
H.lupulus-HL.SW.v1.0.G042778.1                     ------------------------------------------------------------

L.usitatissimum-Lus10040086                        GTTGATTTCTACAATTGCAGATAAGAAGTCTTGGACACGACCGCCAATTCAAATGGAGTT
Marijuana-scaffold38061                            GCTGATTTCGACAATGACAGAAAAGAAGTCCTGGACCAGGCCACCAATTCAGATGGAATT
Textile-Hemp-csa_locus_5464_iso_6_len_1880_ver_2   GCTGATTTCGACAATGACAGAAAAGAAGTCCTGGACCAGGCCACCAATTCAGATGGAATT
H.lupulus-HL.SW.v1.0.G042778.1                     ------------------------------------------------------------

L.usitatissimum-Lus10040086                        CCAGGTCCCCATGTTTACAGCATCTGGATTGCGTGTCAGATTTCTTAAGGTGTGGGAGAA
Marijuana-scaffold38061                            CCAGGT-----------------------------------------AGGTaTGGGAGAA
Textile-Hemp-csa_locus_5464_iso_6_len_1880_ver_2   CCAGGTTCCAATGTTTACAGCATCTGGTCTTCGAGTACGATTTCTTAAGGTGTGGGAGAA
H.lupulus-HL.SW.v1.0.G042778.1                     -----------------------------------------------AGGTATGGGAGAA
                                                                                                  ****.********

L.usitatissimum-Lus10040086                        GAGTGGTTACAACACTGTCGAGTGGGTTCGCTACATTACAAAAGCAGGATCTTACGAGAT
Marijuana-scaffold38061                            GAGTGGATACAACACAGTTGAGTGGGTTCGCTATATCACGAAAGCTGGTTCATACGAAAT
Textile-Hemp-csa_locus_5464_iso_6_len_1880_ver_2   GAGTGGATACAACACAGTTGAGTGGGTTCGCTATATCACGAAAGCTGGTTCATACGAAAT
H.lupulus-HL.SW.v1.0.G042778.1                     GAGTGGATACAACACAGTTGAGTGGGTTCGCTATATCACGAAAGCTGGCTCATACGAAAT
                                                   ******:********:** ************** ** **.*****:** **:*****.**

L.usitatissimum-Lus10040086                        TAGGTGCTAG--------------------------------------------------
Marijuana-scaffold38061                            TAGGTGCTAAAAACATTCGCGGTAATGGTGCAAGAATTGAGATATTTGAGTTTGGAGATC
Textile-Hemp-csa_locus_5464_iso_6_len_1880_ver_2   TAGGTGCTAAAAACATTCGCGGTAATGGTGCAAGAATTGAGATATTTGAGTTTGGAGATC
H.lupulus-HL.SW.v1.0.G042778.1                     TAGGTGCTAAAAAACTTCGGGGTATTGACGCAAGAATTGAGATATTTGAATTTGGAGATT
                                                   *********.

L.usitatissimum-Lus10040086                        ------------------------------------------------------------
Marijuana-scaffold38061                            TACAAATTTGGTTATTGGATCAGAAAATTGTTGCTAAGACATTAGGAATATTTTTTTCTT
Textile-Hemp-csa_locus_5464_iso_6_len_1880_ver_2   TACAAATTTGGTTATTGGATCAGAAAATTGTTGCTAAGACATTAGGAATATTTTTTTCTT
H.lupulus-HL.SW.v1.0.G042778.1                     TATAAATT-TGTTATTCGATCAGAAAGTTGTTGCTTAGCAATAAATCAT---TAGGGCA-

L.usitatissimum-Lus10040086                        ------------------------------------------------------------
Marijuana-scaffold38061                            TTTGGGTTTGTTGTTGTTGGTTATAGTGTTATGTCGATATGATT----GTGTTCATCACT
Textile-Hemp-csa_locus_5464_iso_6_len_1880_ver_2   TTTGGGTTTGTTGTTGTTGGTTATAGTGTTATGTCGATATGATT----GTGTTCATCACT
H.lupulus-HL.SW.v1.0.G042778.1                     ---TTTTTTGTTGTTGTTGGTTATAGTGTTATGTCGATATGATTGTTTGTGTTAATTACT

L.usitatissimum-Lus10040086                        ------------------------------------------------------------
Marijuana-scaffold38061                            TTTtGTTTGATATTTTGTTGTAAATGCTAGCTATTTTTTTaCCCATATCCTTTTACAACT
Textile-Hemp-csa_locus_5464_iso_6_len_1880_ver_2   T-TTGTTTGATATTTTGTTGTAAATGCTAGCTATTTTTTTCCCCATATCCTTTTACAACT
H.lupulus-HL.SW.v1.0.G042778.1                     T-TTGTTTGATATCTTGTTGTAAATGCTAGCTATTTTTTCCC----ATCCTCTTACAACT

L.usitatissimum-Lus10040086                        ------------------------------------------------------------
Marijuana-scaffold38061                            GgCAATaTGAGAATATCTTTGATCTGTTcTTTTCTAGAGATGGATTTGTTCATTCATGGT
Textile-Hemp-csa_locus_5464_iso_6_len_1880_ver_2   GCCAATGTGAGAATATCTTTGATCTGTTATTTTCTAGAGATGGATTTGTTCATTCATGGT
H.lupulus-HL.SW.v1.0.G042778.1                     GGCCATGTGAGAATTTCTTTGATCTGCCCATTTT-------GGAGA-CCAACTTCTTGGT

L.usitatissimum-Lus10040086                        ------------------------------------------------------------
Marijuana-scaffold38061                            TTGCATTTGGCAAAAATT---CAGTTGTAGTGACAAAAcTTGTAAGTGAAGAATACTTGA
Textile-Hemp-csa_locus_5464_iso_6_len_1880_ver_2   TTGCATTTGGCAAAAATT---CAGTTGTAGTGACAAAAGTTGTAAGTGAAGAATACTTGA
H.lupulus-HL.SW.v1.0.G042778.1                     TTGAATTTTGCCAAAATTCTGCTGTTGTAGTGACAAAAATTGCGAGTGAAGAATACTTGA

L.usitatissimum-Lus10040086                        ------------------------------------------
Marijuana-scaffold38061                            CTTGAGATATATAGTTTGCTTTGCCTTTA-------------
Textile-Hemp-csa_locus_5464_iso_6_len_1880_ver_2   CTTGAGATATATAGTTTGCTTTGCCTTTAAAAAAAAAAAAAA
H.lupulus-HL.SW.v1.0.G042778.1                     CTTGAGATAT--AGTTTGCCTTG-------------------
```

F-box

```
H.lupulus-HL.SW.v1.0.G001325.1                    ------------------------------------------------------------
L.usitatissimum-LUS10031955                       ------------------------------------------------------------
Textile-hemp-csa_locus_16122_iso_2_len_2075_ver_2 ------------------------------------------------------------
Marijuana-scaffold20047                           TGAAAACTTTCTTTGTCTATTTGTCGAATAGTGACAAAAATAAATCAAACGCATCTACAG

H.lupulus-HL.SW.v1.0.G001325.1                    ------------------------------------------------------------
L.usitatissimum-LUS10031955                       ------------------------------------------------------------
Textile-hemp-csa_locus_16122_iso_2_len_2075_ver_2 ----------------------------------------TCACCAAGAGCCAATTCAGA
Marijuana-scaffold20047                           GAGAGAGAGAGAGAGAGAGAGAGAGAGAGAGAGTTGAAGTTCACCAAGAGCCAATTCAGA

H.lupulus-HL.SW.v1.0.G001325.1                    ------------------------------------------------------------
L.usitatissimum-LUS10031955                       ------------------------------------------------------------
Textile-hemp-csa_locus_16122_iso_2_len_2075_ver_2 AAATTTTATGATTCTTTCTACCCACAAAGCCTACAGGTTTGTAATCCTTGGATAAATTTT
Marijuana-scaffold20047                           AAATTTTATGATTCTTTCTACCCACAAAGCCTACAGGTTTGTAATCCTTGGATAAATTTT

H.lupulus-HL.SW.v1.0.G001325.1                    ------------------------------------------------------------
L.usitatissimum-LUS10031955                       ------------------------------------------------------------
Textile-hemp-csa_locus_16122_iso_2_len_2075_ver_2 TGGTGAATTTTTTTTCGTCCTTTTTTGCAAAAAATTCAAGCAAATCCTTGATTTATTGAG
Marijuana-scaffold20047                           TGGTGAATTTTTTTTCGTCCTTTTTTGCAAAAAATTCAAGCAAATCCTTGATTTATTGAG

H.lupulus-HL.SW.v1.0.G001325.1                    ------------------------------------------------------------
L.usitatissimum-LUS10031955                       ------------------------------------------------------------
Textile-hemp-csa_locus_16122_iso_2_len_2075_ver_2 CTTGGATCTGAGAAAAAGATTAGGGTTTTGTTGTTTCTTTCATCCCATTGGGTATGGATT
Marijuana-scaffold20047                           CTTGGATCTGAGAAAAAGATTAGGGTTTTGTTGTTTCTTTCATCCCATTGGGTATGGATT

H.lupulus-HL.SW.v1.0.G001325.1                    ------------------------------------------------------------
L.usitatissimum-LUS10031955                       ------------------------------------------------------------
Textile-hemp-csa_locus_16122_iso_2_len_2075_ver_2 TCTTGTTCCTGTGAATCACGGTTGGGAGTAAGGTTATTTGATTTGTGGGGGTGCATGGGG
Marijuana-scaffold20047                           TCTTGTTCCTGTGAATCACGGTTGGGAGTAAGGTTATTTGATTTGTGGGGGTGCATGGGG

H.lupulus-HL.SW.v1.0.G001325.1                    ------------------------------------------------------------
L.usitatissimum-LUS10031955                       ------------------------------------------------------------
Textile-hemp-csa_locus_16122_iso_2_len_2075_ver_2 GACATGTATTTTGAGTTGAGCATTGGTAAATGGTAATTAGCTCAATGTAGGAACTGGGTC
Marijuana-scaffold20047                           GACATGTATTTTGAGTTGAGCATTGGTAAATGGTAATTAGCTCAATGTAGGAACTGGGTC

H.lupulus-HL.SW.v1.0.G001325.1                    ------------------------------------ATGGGTGATATTGGAGAATCTTCT
L.usitatissimum-LUS10031955                       ------------------------------------ATGGATGAAATTGGAGAATCATCG
Textile-hemp-csa_locus_16122_iso_2_len_2075_ver_2 ATTAGGATTTTTCTTGTTTGTAGGAAATTGATCAAAATGGGTGATATTGGAGAATCTTCT
Marijuana-scaffold20047                           ATTAGGATTTTTCTTGTTTGTAGGAAATTGATCAAAATGGGTGATATTGGAGAATCTTCT
                                                                                      ****.***:***********:**

H.lupulus-HL.SW.v1.0.G001325.1                    GAATCTGGGTCTGTAGTGT---GTGCGAGAAATGGAT---------TTTGCCGAGAAGAA
L.usitatissimum-LUS10031955                       GAATCTGCGTCAGCTTTAGCTAGTCCTAGATCGGTGATAACATATGGATACCGCGATGAA
Textile-hemp-csa_locus_16122_iso_2_len_2075_ver_2 GAATTTGGGTCTGCAGTGAAT---ACGAGAAATGGGT---------TTTGTCATGAAGAA
Marijuana-scaffold20047                           GAATTTGGGTCTGCAGTGAAT---ACGAGAAATGGGT---------TTTGTCATGAAGAA
                                                  **** ** ***:* : *.       * ***:. * .:          :*. *. **:***

H.lupulus-HL.SW.v1.0.G001325.1                    GAGAGATGTCCGAAGCAAGTTTCCCCTGTTAGAGGTGGTGGGTCGAGGAATACAAGTCCA
L.usitatissimum-LUS10031955                       GGTTGTTGCCCTAGGCAAGTTTCACCGATTAGGATTGGCGGGTCGAGGAACACCAGCCCC
Textile-hemp-csa_locus_16122_iso_2_len_2075_ver_2 GTGAGATGTCCGAAGCAAATTTCCCCTGTTAGAGGTGGTGGGTCGAGGAATACGAGTCCA
Marijuana-scaffold20047                           GTGAGATGTCCGAAGCAAATTTCCCCTGTTAGAGGTGGTGGGTCGAGGAATACGAGTCCA
                                                  *  :*:** ** *.****.****.** .****.. *** *********** ** ** **.

H.lupulus-HL.SW.v1.0.G001325.1                    TTAGGGCGTGTCGGATCGAGAAACACTAGTCCGTCTAGGCAGAAGGTGATCAAGACAAAA
L.usitatissimum-LUS10031955                       ATGGGTCGAGTTGGGTCTAGAAACACCTGCCCTTTGAAGCAGAAAGTAGTCATGACTAAA
Textile-hemp-csa_locus_16122_iso_2_len_2075_ver_2 TTAGGTCGTGTCGGATCGAGAAACACTAGTCCATCTAGGCAGAAGGTGATCAAGACAAAG
Marijuana-scaffold20047                           TTAGGTCGTGTCGGATCGAGAAACACTAGTCCATCTAGGCAGAAGGTGATCAAGACAAAG
                                                  :*.** **:** **.** ******** :* ** *  *.******.**..***:***:**.

H.lupulus-HL.SW.v1.0.G001325.1                    CCTCGAGGATTAGATGAGGAAACGGCTGCC---ACATTTGGTAAAGCAATCCACC---CG
L.usitatissimum-LUS10031955                       CCCCGTGGTTTGGATGAAGAGACTGCGACCACCACATTTGGCAAGGCTATTTACTATTGT
Textile-hemp-csa_locus_16122_iso_2_len_2075_ver_2 CCTCGAGGATTGGATGAGGAAACGGCTGCC---ACATTTGCTAAAGCAGTTCACC---CG
Marijuana-scaffold20047                           CCTCGAGGATTGGATGAGGAAACGGCTGCC---ACATTTGCTAAAGCAGTTCACC---CG
                                                  ** **:**:**.*****.**.** ** .**   *******  **.**:.*  **

H.lupulus-HL.SW.v1.0.G001325.1                    GATGTTCAGATGGAAGATAATATCTGGGCAATGTTGCCTGAGGATTTGCTGAATGAGATC
L.usitatissimum-LUS10031955                       GATGTTCAGATGGAGGACAACATATGGGCAATGCTGCCCGAAGATCTCTTGAACGAGATC
Textile-hemp-csa_locus_16122_iso_2_len_2075_ver_2 GATGTTCAGATGGAAGATAATATCTGGGCAATGTTGCCTGAGGATTTGCTGAATGAGATT
Marijuana-scaffold20047                           GATGTTCAGATGGAAGATAATATCTGGGCAATGTTGCCTGAGGATTTGCTGAATGAGATT
                                                  **************.** ** **.********* **** **.*** *  **** *****

H.lupulus-HL.SW.v1.0.G001325.1                    TTAGCTAGGGTTCCTCCATTTATGATATTTCGACTCCGCTGTGTTTGCAGAAGATGGAAT
L.usitatissimum-LUS10031955                       TTAGCTCGAGTTCCGCCGTTCATGATCTTTCGCCTTCGATCGGTTTGCAAGCGGTGGAAT
Textile-hemp-csa_locus_16122_iso_2_len_2075_ver_2 TTAGCTAGGGTTCCTCCATTTATGATATTTCGGCTTCGTTGTGTTTGTAGAAGGTGGAAT
Marijuana-scaffold20047                           TTAGCTAGGGTTCCTCCATTTATGATATTTCGGCTTCGTTGTGTTTGTAGAAGGTGGAAT
                                                  ******.*.***** **.** *****.***** ** ** *  ***** *...*.******

H.lupulus-HL.SW.v1.0.G001325.1                    TTAATTCTTCAAGATAGTAGCTTTCTCAAATTCCATTCCCAAGTACCTTCCCACGGGCCT
L.usitatissimum-LUS10031955                       TCGATCTTGCAAGACATCAGCTTTCTCAAATTCCACTCGCAGGTGCCTTCTCACGGACCG
Textile-hemp-csa_locus_16122_iso_2_len_2075_ver_2 TTAATCCTTCAAGATAGTAGCTTTCTCAAATTCCATTCCCAAGCACCTTCTCACGGGCCT
Marijuana-scaffold20047                           TTAATCCTTCAAGATAGTAGCTTTCTCAAATTCCATTCCCAAGCGCCTTCTCACGGGCCT
                                                  * .**  * ***** *  ***************** ** **.* .***** *****.**

H.lupulus-HL.SW.v1.0.G001325.1                    TGTCTTCTCACATTTTGGAAGAATTCCCAGACTCCACAATGCTCGGTTTTCAGCTTGCCA
L.usitatissimum-LUS10031955                       TGTCTCTTTGCATTCTGGAAAAACTCGCAGACTCCGCAGTGTTCTGTTCTCAGCTTGCCG
Textile-hemp-csa_locus_16122_iso_2_len_2075_ver_2 TGTCTTCTCACATTCTGGAAGAATTCACAGACTCCGCAATGTTCGGTATTCAGCTTGCCA
Marijuana-scaffold20047                           TGTCTTCTTACATTCTGGAAGAATTCACAGACTCCGCAATGTTCGGTATTCAGCTTGCCA
                                                  *****  * .**** *****.** ** ********.**.** ** **: **********.

H.lupulus-HL.SW.v1.0.G001325.1                    TTGAAGACATGGTATAAGATTCCTTTCACATTTTTGCCACAGTGGGCATTCTGGTTGGTT
L.usitatissimum-LUS10031955                       TTGAAGACATGGTATCGAATTCCTTTCACGTTCTTACCGCAGTGGGCGGTTTGGTTGGTT
Textile-hemp-csa_locus_16122_iso_2_len_2075_ver_2 TTAAAGACATGGTATAGGATTCCTTTCACATTTTTGCCTCAATGGGCATTCTGGTTAGTT
Marijuana-scaffold20047                           TTAAAGACATGGTATAGGATTCCTTTCACATTTTTGCCTCAATGGGCATTCTGGTTAGTT
                                                  **.************...***********.** **.** **.*****. * *****.***
```

```
H.lupulus-HL.SW.v1.0.G001325.1                             GGTTCTTCTGGTGGTCTTGTTTGCTTTTCTGGGCTTGATGGCCTAACGTTCAAAACTTTA
L.usitatissimum-LUS10031955                                GGTTCTTCGGGCGGACTCGTTTGCTTTTCCGGACTCGATGGGTTGACTTTCAAGTCATTG
Textile-hemp-csa_locus_16122_iso_2_len_2075_ver_2          GGTTCTTCTGGTGGTCTAATTTGCTTTTCTGGGCTAGATGGGCTAACTTTCAGAACTTTA
Marijuana-scaffold20047                                    GGTTCTTCTGGTGGTCTAATTTGCTTTTCTGGGCTAGATGGGCTAACTTTCAGAACTTTA
                                                           ******** ** **:** .********** **.** *****  *.** ****..:*:**.

H.lupulus-HL.SW.v1.0.G001325.1                             GTTTGCAATCCACTCACACAAACCTGGAGATCTCTACCAAATATGCATTACAATCAGCAA
L.usitatissimum-LUS10031955                                GTTTGCAATCCTTTGACTCAGACATGGAGGACTCTTCCCAGCATGCACAATAATCAGCAG
Textile-hemp-csa_locus_16122_iso_2_len_2075_ver_2          GTTTGTAATCCACTTACACAAACGTGGAGGGCTCTACCAACTATGCATTACAACCAGCAA
Marijuana-scaffold20047                                    GTTTGTAATCCACTTACACAAACGTGGAGGGCTCTACCAACTATGCATTACAACCAGCAA
                                                           ***** *****: * **:**.** *****. ****:**.*  ***** :* ** *****.

H.lupulus-HL.SW.v1.0.G001325.1                             AGACAGCTGATCATGGTTGTTGATCGTAAACATCGGTCATTTAAAGTTATAGCCACAAGT
L.usitatissimum-LUS10031955                                AGACAGTTGATATTGGTGGTCGATCGTTCGGACCGGTCGTTCAAAGTGATAGCTACCAGC
Textile-hemp-csa_locus_16122_iso_2_len_2075_ver_2          AGACAGCTGATCATGGTTGTTGATCGAAAACAACGGTCATTCAAAGTTATAGCAACAAGT
Marijuana-scaffold20047                                    AGACAGCTGATCATGGTTGTTGATCGAAAACAACGGTCATTCAAAGTTATAGCAACAAGT
                                                           ****** ****.:**** ** *****::.. * *****.** ***** ***** **.**

H.lupulus-HL.SW.v1.0.G001325.1                             GACATTTATGGTGACAAATCATTACCCACCGAAGTGTATGATTCAAAGCTAAATAGTTGG
L.usitatissimum-LUS10031955                                GATGTTTATGGCGATAAATCGTTGCCTACCGAAGTGTACGATTCGAAACTCGACAGATGG
Textile-hemp-csa_locus_16122_iso_2_len_2075_ver_2          GATATTTACGGTGACAAATCATTACCCACCGAAGTGTATGATTCTAGGCTGAATAGTTGG
Marijuana-scaffold20047                                    GATATTTACGGTGACAAATCATTACCCACCGAAGTGTATGATTCTAGGCTGAATAGTTGG
                                                           ** .**** ** ** *****.**.** *********** ***** *..** .* **:***

H.lupulus-HL.SW.v1.0.G001325.1                             TCCCTTCACCAGATAATGCCAGCAGTTAATCTTTGCTCCTCAAAGATGGCTTATTGTGAC
L.usitatissimum-LUS10031955                                GTTGTTCATCAGATTATGCCTGCCGTTAATCTATGCTCCTCGAAGATGGCGTATTGCGAC
Textile-hemp-csa_locus_16122_iso_2_len_2075_ver_2          TTCCTTCACCAGATAATGCCAGCAGTTAATCTTTGCTCCTCGAAGATGGCCTATTGTGAT
Marijuana-scaffold20047                                    TTCCTTCACCAGATAATGCCAGCAGTTAATCTTTGCTCCTCGAAGATGGCCTATTGTGAT
                                                               **** *****:*****:**.********:********.******** ***** **

H.lupulus-HL.SW.v1.0.G001325.1                             TCCAGGTTGTATTTAGAATCTCTTTCGCCACTCGGGTTGATGATGTATAGACTGGACACT
L.usitatissimum-LUS10031955                                TCGAGATTATACCTCGAGACTCTTTCTCCGCTCGGTTTGATGATGTATAAGCTCGACACG
Textile-hemp-csa_locus_16122_iso_2_len_2075_ver_2          TCCAGATTGTATTTAGAAACCCTTTCTCCACTAGGATTGATGATGTATAAACTGGACACT
Marijuana-scaffold20047                                    TCCAGATTGTATTTAGAAACCCTTTCTCCACTAGGATTGATGATGTATAAACTGGACACT
                                                           ** **.**.**  *.**.:* ***** **.**.** *************..** *****

H.lupulus-HL.SW.v1.0.G001325.1                             GGTTACTGGGAACACATTCATGCTAAATTCCCACGATCTTTACTGGATGGCTACTTGGTT
L.usitatissimum-LUS10031955                                GGTTACTGGGAGCATATTCCTGCGAAGTTTCCACGGTCTCTGTTGGATGGATACTTGGTG
Textile-hemp-csa_locus_16122_iso_2_len_2075_ver_2          GGATATTGGGAACACATTCCTGCTAAATTCCCACGATCTCTACTGGATGGGTACTTGGTT
Marijuana-scaffold20047                            GGATATTGGGAACACATTCCTGCTAAATTCCCACGATCTCTACTGGATGGGTACTTGGTT
                                                           **:** *****.** ****.*** **.** *****.*** *. ******* ********

H.lupulus-HL.SW.v1.0.G001325.1                             GCTGGCACACAGAAGCGTCTATTTTTAGTTGGACGCATTGGTCTTTACAGTACTCTTCAA
L.usitatissimum-LUS10031955                                GCTGGAACCCAGAAGCGGTTGTTTTTGGTGGGGAGAATCGGCCTATATAGTACGCTTCAG
Textile-hemp-csa_locus_16122_iso_2_len_2075_ver_2          GCTGGCACACAGAAGCGTCTGTTTTTAGTTGGACGCATTGGTCTCTATAGTACTCTTCAA
Marijuana-scaffold20047                                    GCTGGCACACAGAAGCGTCTGTTTTTAGTTGGACGCATTGGTCTCTATAGTACTCTTCAA
                                                           *****.**.********  *.*****.** **..*.** ** ** ***** *****.

H.lupulus-HL.SW.v1.0.G001325.1                             AGTATGAGAATTTGGGAATTGGATCATGCGAAAACTATGTGGGTGGAGATCAGTAGAATG
L.usitatissimum-LUS10031955                                AGTATGAGGATTTGGGAGTTGGATCATGCGAAGAACTTGTGGCTAGAGATTAGTAGAATG
Textile-hemp-csa_locus_16122_iso_2_len_2075_ver_2          AGTATGAGAATTTGGGAATTGGATCATGCAAAAGTTATGTGGGTGGAGATTAGTAGGATG
Marijuana-scaffold20047                                    AGTATGAGAATTTGGGAATTGGATCATGCAAAAGTTATGTGGGTGGAGATTAGTAGGATG
                                                           ********.********.***********.**..   :***** *.***** *****.***

H.lupulus-HL.SW.v1.0.G001325.1                             CCACCAAAGTATTTTCGAGCACTGTTAAGATTATCTGCTGAGAGATTTGAGTGTTTTGGA
L.usitatissimum-LUS10031955                                CCACCAAAGTATTTCAGGACGTTGCTGAGGCTGTCGGCTGATCGGTTCGAGTGCTTTGGA
Textile-hemp-csa_locus_16122_iso_2_len_2075_ver_2          CCACCGAAGTATTTTCGAGCACTGTTGAGATTATCGGCGGAGAGATTTGAGTGCTTTGGA
Marijuana-scaffold20047                                    CCACCGAAGTATTTTCGAGCACTGTTGAGATTATCGGCGGAGAGATTTGAGTGCTTTGGA
                                                           *****.******** .*..*. ** *.**. *.** ** ** .*.** ***** ******

H.lupulus-HL.SW.v1.0.G001325.1                             CAGGATAACTTAATCTGCTTTACCTCTTGGAATCAAGGGAAGGGCTTATTGTATGATGTG
L.usitatissimum-LUS10031955                                CAGGATAACTTGATATGTTTCACATCTAGGAACCAAGGGAAGGGTTTGTTGTATGATGTG
Textile-hemp-csa_locus_16122_iso_2_len_2075_ver_2          CAGGATAACTTAATCTGCTTTACATCTTGGAATCAAGGGAAGGGCTTATTGTATGATGTT
Marijuana-scaffold20047                                    CAGGATAACTTAATCTGCTTTACATCTTGGAATCAAGGGAAGGGCTTATTGTATGATGTT
                                                           ***********.**.** ** **.***:**** *********** **.***********

H.lupulus-HL.SW.v1.0.G001325.1                             GATAAAAAGGCTTGGTCTTGGATAGGTGGATGTGCTCTGCAGTCTTACAACAGCCAGGTT
L.usitatissimum-LUS10031955                                GATAAGAAGGTGTTGTCGTGGATCGGAGGATGTCCTCTTGAATCATATAACAGCCAGGTG
Textile-hemp-csa_locus_16122_iso_2_len_2075_ver_2          GATAAAAAGTCTTGGTCTTGGATAGGTGGATGTGCTCTGCAGTCTTACAACAGCCAGGTT
Marijuana-scaffold20047                                    GATAAAAAGTCTTGGTCTTGGATAGGTGGATGTGCTCTGCAGTCTTACAACAGCCAGGTT
                                                           *****.***   * *** *****.**:****** ****   *.**:** ***********

H.lupulus-HL.SW.v1.0.G001325.1                             TGTTTTTATGAGCCAAGATTTGATGCTTCCATCTTTTAATTAGGAAATTTTGCTCTGCGG
L.usitatissimum-LUS10031955                                TGCTTCTATGAGCCTAGATTTGATGCTTCAATCTATTGA---------------------
Textile-hemp-csa_locus_16122_iso_2_len_2075_ver_2          TGCTTTTACGAACCAAGATTTGATGCTTCCATTTGTTAATTAATATTTAATG-------G
Marijuana-scaffold20047                                    TGCTTTTACGAACCAAGATTTGATGCTTCCATTTGTTAATTAATATTTAATG-------G
                                                           ** ** ** **.**:**************.** * **.*

H.lupulus-HL.SW.v1.0.G001325.1                             CTTCGAGTTCTATTTATTAGTCAGAGAATTTATGAGAAGATATCATTTTTCTCATGGCAT
L.usitatissimum-LUS10031955                                ------------------------------------------------------------
Textile-hemp-csa_locus_16122_iso_2_len_2075_ver_2          C--------------------CACAG-AACTATAAGAAGATATCAT-TTTCACCTGGCAT
Marijuana-scaffold20047                                    C--------------------CACAG-AACTATAAGAAGATATCAT-TTTCACCTGGCAT

H.lupulus-HL.SW.v1.0.G001325.1                             ATTTT--TTTTGGTATCAATCCTTCTTTCCCCACTTCTGCACATTGACATCTAAGTTTTC
L.usitatissimum-LUS10031955                                ------------------------------------------------------------
Textile-hemp-csa_locus_16122_iso_2_len_2075_ver_2          ATTTTTGTTTTTGTCTCAATCTTTCTTTCCCCAGTTTTACACATTGACATCTAAGTCTTC
Marijuana-scaffold20047                                    ATTTTTGTTTTTGTCTCAATCTTTCTTTCCCCAGTTTTACACATTGACATCTAAGTCTTC

H.lupulus-HL.SW.v1.0.G001325.1                             TTCATATTGAAATTGTTAGCTAACAATTCAGATTCTT-TTCACTGTGAATCTGCTCTATG
L.usitatissimum-LUS10031955                                ------------------------------------------------------------
Textile-hemp-csa_locus_16122_iso_2_len_2075_ver_2          TTCATATTGAATTTGTTAGCTAACAATTCAGATTCTTTTTCACTGTGAATCTGTTACATG
Marijuana-scaffold20047                                    TTCATATTGAATTTGTTAGCTAACAATTCAGATTCTTTTTCACTGTGAATCTGTTACATG

H.lupulus-HL.SW.v1.0.G001325.1                             AGTGTATCCGAGTAAGACCTTT-TTGTTAGCAGAATTTATGTACATTTACATTTGGTGGT
L.usitatissimum-LUS10031955                                ------------------------------------------------------------
Textile-hemp-csa_locus_16122_iso_2_len_2075_ver_2          GGTGTATCCGAGTTAGACCTTTTTGTTAGCTAAATTTATTTGCGTTCACAATTGGTGGT
Marijuana-scaffold20047                                    GGTGTATCCGAGTTAGACCTTTTTGTTAGCTAAATTTATTTGCGTTCACAATTGGTGGT

H.lupulus-HL.SW.v1.0.G001325.1                             GCATCAGCTTTGTACCGGGGCTGTGCGAGGAATATGACAAGCAATGTTGTAAG-----TT
L.usitatissimum-LUS10031955                                ------------------------------------------------------------
```

```
Textile-hemp-csa_locus_16122_iso_2_len_2075_ver_2      GCATCAACTTTGTACCAGGGCTGTGCCAGGAATATGACAAGCAATGTATTGAGTTGAGTT
Marijuana-scaffold20047                                GCATCAACTTTGTACCAGGGCTGTGCCAGGAATATGACAAGCAATGTATTGAGTTGAGTT

H.lupulus-HL.SW.v1.0.G001325.1                         GAGTTGGGACCAAATTTGGGTCCTTTGGGTTTGTAATAAGAATGTAGGTTGGCTGGGGAG
L.usitatissimum-LUS10031955                            ------------------------------------------------------------
Textile-hemp-csa_locus_16122_iso_2_len_2075_ver_2      GAGTTGAGACCAAATTTGGGTCCTTTGGGCTTGTGTAATAATTT----------------
Marijuana-scaffold20047                                GAGTTGAGACCAAATTTGGGTCCTTTGGGCTTGTGTAATAATTT----------------

H.lupulus-HL.SW.v1.0.G001325.1                         GAAATGTGAGAAGGCTCTTT-CTTGAACTGTTGTACACTATTTAAAACAATTTGCACATT
L.usitatissimum-LUS10031955                            ------------------------------------------------------------
Textile-hemp-csa_locus_16122_iso_2_len_2075_ver_2      -AAATTTAAGAAGGCTCTTTTTTTGAACTGTTGTACA------------ATCTGCACATT
Marijuana-scaffold20047                                -AAATTTAAGAAGGCTCTTTTTTTGAACTGTTGTACA------------ATCTGCACATT

H.lupulus-HL.SW.v1.0.G001325.1                         AATATGTAAATTATAAAGTTTTGCTTTT--------------------------------
L.usitatissimum-LUS10031955                            ------------------------------------------------------------
Textile-hemp-csa_locus_16122_iso_2_len_2075_ver_2      AATTTGTATATCATAAAGTTTTACTTTTAAA-----------------------------
Marijuana-scaffold20047                                AATTTGTATATCATAAAGTTTTACTTTTATCACTATAGTAATTTTTAAACTTAGTTTATG

H.lupulus-HL.SW.v1.0.G001325.1                         ------------------------------------------------------------
L.usitatissimum-LUS10031955                            ------------------------------------------------------------
Textile-hemp-csa_locus_16122_iso_2_len_2075_ver_2      ------------------------------------------------------------
Marijuana-scaffold20047                                TATTGCAAGTAAACTAGAGTTGATTTTTTTTTTTTTTTTTTTTTGATTAACAACTAGAGT

H.lupulus-HL.SW.v1.0.G001325.1                         ---------
L.usitatissimum-LUS10031955                            ---------
Textile-hemp-csa_locus_16122_iso_2_len_2075_ver_2      ---------
Marijuana-scaffold20047                                TGATTTAAA
```

Figure S3. Alignment of the hemp reference genes and the orthologs from flax, hop and marijuana. The primers described in the present work are underlined and the amplified region is highlighted in yellow.
